# Mining Alzheimer's Interactomes, Macromolecular Complexes and Pathways for Drug Discovery

**DOI:** 10.1002/pmic.70018

**Published:** 2025-08-25

**Authors:** Kalpana Panneerselvam, Krishna Kumar Tiwari, Luana Licata, Simona Panni, Sylvie Ricard‐Blum, Sucharitha Balu, Susie Huget, Juan Jose Medina Reyes, Eliot Ragueneau, Livia Perfetto, Birgit Meldal, Sandra Orchard, Henning Hermjakob

**Affiliations:** ^1^ European Bioinformatics Institute (EMBL‐EBI), European Molecular Biology Laboratory, Hinxton Cambridge UK; ^2^ University of Rome Tor Vergata Macro Area of Mathematical Physical and Natural Sciences Roma Lazio Italy; ^3^ University of Calabria Department of Biology Ecology and Earth Sciences Arcavacata di Rende Calabria Italy; ^4^ Universite Lyon 1 IUT Lyon 1 Villeurbanne France; ^5^ University of Rome La Sapienza Faculty of Mathematical Physical and Natural Sciences Roma Italy; ^6^ Pfizer Institute for Pharmaceutical Materials Science Cambridge UK

## Abstract

Alzheimer's disease (AD) is a progressive neurodegenerative disorder that leads to dementia. Many cases are diagnosed annually and there is no currently available cure. Understanding the underlying disease biology of AD through the study of molecular networks, particularly by mapping clinical variants to tissue‐specific interactomes and regulatory macromolecular assemblies, offers a promising avenue to elucidate altered disease pathways. In this study, we applied differential interactome analysis using a manually curated AD dataset to identify how disease‐associated mutations alter both transient and stable protein interactions. By focussing on variant‐specific associations detected in brain‐relevant tissues, we mapped disruptions in stable macromolecular assemblies and performed Reactome enrichment analysis to uncover perturbed pathways unique to each variant. Additionally, we explored therapeutic insights through the analysis of amyloid precursor protein (APP) physical interactors, identifying potential intervention points that influence amyloidogenic processing. Complementing protein‐level data, we integrated microRNA (miRNA)‐mediated regulatory interactions, revealing an additional layer of posttranscriptional control over key AD genes. Together, this multilayered strategy provides a framework for precision therapeutics in AD.

## 1 Introduction

Alzheimer's disease (AD) remains a formidable adversary in the realm of neurodegenerative disorders, challenging both the scientific community and medical practitioners alike. A definitive cure for this devastating ailment remains elusive, accentuating the urgency to explore novel avenues in understanding its clinical pathogenesis and identifying potential therapeutic options.

The emerging concepts of network biology and pathway analysis have reshaped our understanding of complex diseases like Alzheimer's (Wu et al. 2014). Instead of viewing individual genes or proteins in isolation, these approaches interrogate the intricate web of interactions that govern biological systems. This shift in perspective has opened the door to more meaningful insights into the underlying pathogenesis of AD, offering a renewed sense of hope in our quest for effective treatments.

The IMEx Consortium [1] has evolved into a vital partnership of open resources dedicated to curating molecular interaction (MI) data from the scientific literature. This consortium, a collaborative effort including IntAct [2], MINT [3], MatrixDB [4], UniProtKB [5] and DIP [6], is driven by the central mission of aggregating fine‐grained experimental interaction evidence in a machine‐readable format, supported by controlled vocabularies and standard ontologies. Members of the IMEx Consortium have been active in supporting research into neurodegenerative disease for many years [7, 8, 9] through both targeted curation and standards development.

Interactions in biological systems can involve two or more molecules, and the number of observable interactors can vary depending on biological and experimental factors. For example, technologies like yeast‐two‐hybrid arrays typically identify only pairs of interactors (binary interactions), while methods such as tandem affinity purification (TAP) combined with mass spectrometry (MS) [10] and BioID [11] may capture two or more interacting molecules (n‐ary interactions). In most data resources, these n‐ary interactions are expanded into multiple binary interactions for visualisation and data analysis.

Although binary interactions offer detailed insights into interactions such as binding domains, affinity tags, interaction kinetics and mutations affecting the interaction, n‐ary interactions provide a broader view of complex MIs within biological systems. Sequence changes in proteins, resulting from genetic mutations or modifications, can significantly impact the nature and outcome of interactions within biological networks. The IMEx Consortium has been instrumental in gathering data on mutations and their effects on MIs, employing controlled vocabularies to ensure consistency and organisation of this information [12].

Pathway enrichment analysis is a fundamental tool in systems biology, enabling the interpretation of interaction networks by identifying overrepresented biological processes and disease‐relevant pathways [8]. In AD, such analysis is crucial for elucidating the molecular mechanisms involving two hallmark proteins: amyloid precursor protein (APP, UniProtKB: P05067) and tau (MAPT, UniProtKB: P10636). APP cleavage generates neurotoxic amyloid‐beta (Aβ) peptides, while tau hyperphosphorylation contributes to neurofibrillary tangle formation—both key features of AD pathology.

In this study, we systematically examined the IMEx Alzheimer's dataset, focussing on variant‐ and context‐specific protein–protein interactions. We identified disruptions in stable macromolecular complexes and applied Reactome pathway enrichment to reveal variant‐specific perturbations. To complement this protein‐level perspective, we integrated microRNA (miRNA)‐mediated regulatory interactions, providing an additional layer of posttranscriptional regulation. This integrative, multi‐layered strategy offers a comprehensive framework for dissecting AD mechanisms and informing precision therapeutic approaches.

## 2 IMEx Alzheimer's Dataset Summary

The AD dataset curated by the IMEx Consortium comprises a comprehensive collection of MIs relevant to AD research, that is, interactions involving proteins with a known link to the disease or that have been identified in disease tissue, as well as interactions that are affected by clinical variants. The dataset currently includes more than 63,000 interactions (Table S1), which encompasses both true‐binary interactions and spoke‐expanded binaries. The dataset can be accessed at https://www.ebi.ac.uk/intact/search?query=annot:%22dataset:alzheimers%22 and can be downloaded in tab and xml formats at https://www.ebi.ac.uk/intact/download/datasets.

- *Interactions affected by mutation*: There are 10,021 interactions that are affected by mutations. These interactions can be easily accessed via a dedicated ‘Mutation’ filter, enabling targeted exploration of mutation‐driven network perturbations. The query results can be viewed at the following link https://www.ebi.ac.uk/intact/search?query=annot:%22dataset:alzheimers%22&mutationFilter=true.
- *Types of interactions*:
  ○ 53,000+ Physical Associations: these represent molecules believed to be in close physical contact and include over 1030 direct interactions and more than 100 enzymatic reactions.
  ○ 8831 Associations: these represent indirect or complex interactions that are predominantly detected by affinity purification (AP) combined with MS (AP‐MS). This method allows for the identification of protein complexes in more physiological settings, capturing the complexity and context of protein networks in a more native environment.
  ○ Colocalisation and proximity: this set includes over 1330 interactions that are detected predominantly by proximity‐dependent BioID assays, and there are more than 130 colocalisations.
- *Detection and context*:
  ○ Physical associations have mostly been detected using yeast two‐hybrid assays, highlighting their utility in mapping protein–protein interactions.
  ○ Associations are largely detected in human neurons and other brain‐related tissues, providing contextually relevant data for AD.
- *Human‐human interactions*: The dataset includes 62,263 interactions that have been experimentally demonstrated with human proteins and genes.

## 3 Network Analysis and Enrichment Study

Interactions in brain tissues were extracted from the Alzheimer's dataset (available via the IntAct portal or in miTAB 2.7 format) by filtering for ‘host organism’ annotations corresponding to human brain regions—specifically neurons, hippocampus and the cerebral cortex. Interaction types were restricted to ‘association’ and ‘proximity’, and a mutation filter was applied to isolate mutation‐affected interactions. The resulting data were further stratified into three categories: Tau‐F reference interactors, and those specific to the P301L (rs63751273) and V337M (rs63750570) Tau variants.

Using the IntAct Cytoscape app (Ragueneau, Shrivastava, and Morris 2021), we retrieved interactions among these partners within the AD dataset, excluding first neighbors ‐ to construct a network. This network was subsequently refined using a MIscore threshold of 0.5 to retain only high‐confidence interactions for downstream analysis.

### 3.1 Pathway Enrichment Analysis

High‐confidence interactors (MI score > 0.5) for each Tau variant network were subjected to pathway enrichment analysis using the Reactome Pathway Browser tool (https://reactome.org/PathwayBrowser/#TOOL=AT). The tool applies a binomial test (via the COLT library) to identify overrepresented pathways relative to the full Reactome database. Separate analyses were conducted for Tau‐F, Tau‐F V337M and Tau‐F P301L networks using their respective gene lists (Table S4). Resulting enrichment outputs and summary reports are available in the supplementary folder (https://drive.google.com/drive/folders/1GRfs1MFKACLMNKiXFVb3ql8zkI0LCyRn). For the ease of analysis and better statistical relevance, we have ignored all the enriched pathways with *p* value > 0.05 for each analysis.

### 3.2 Functional Complex Enrichment

To identify enriched macromolecular complexes, we employed ontology files from the Complex Portal [13] using the ClueGO Cytoscape plugin [14]. The Complex Portal (www.ebi.ac.uk/complexportal) is a curated database cataloguing biologically validated protein complexes. In ClueGO (v2.5.8), these complexes are represented as leaf nodes within the Gene Ontology (GO) Cellular Component hierarchy, allowing focussed enrichment analysis on complex composition. These ontology files are updated with every second release of the Complex Portal, ensuring current data is used for interpretation.

### 3.3 APP Interactome

To systematically explore the interaction landscape of the APP, we employed a targeted network analysis strategy using the IMEx Alzheimer's dataset from the IntAct database. We designed a specific Molecular Interaction Query Language (MIQL) query—annot: ‘dataset:alzheimers’ AND id:P05067*—to retrieve all interaction partners of canonical APP (UniProtKB: P05067), its isoforms and postprocessed chains.

This query yielded a robust set of over 1900 true‐binary interactions, predominantly identified through two‐hybrid assays. Remarkably, more than 900 interactions are annotated as being affected by mutations, emphasising their potential relevance to AD mechanisms.

To assess the functional landscape of high‐confidence APP interactors, we performed GO Molecular Function enrichment analysis using ClueGO (Cytoscape plugin) and the latest GO annotations (as of 7 June 2024).

## 4 Alzheimer Dataset: Interactions in Brain Tissues

In the case of AD, the relevance of tissue specificity is obvious. The IMEx Alzheimer's dataset has documented over 6000 interactions detected in brain‐associated tissues—including neurons, the hippocampus and the cerebral cortex [15, 16, 17]. In addition, several hundred interactions are mapped to neuro‐cancer cell lines, offering broader insights into molecular mechanisms relevant to AD pathogenesis. These context‐specific networks help predict the composition of regulatory macromolecular assemblies critical to disease pathophysiology.

Using advanced filtering options on the IntAct platform, we specifically queried interactions detected in human brain tissues. Filters were applied for interaction types (‘association’ and ‘proximity’) and host organism sources, including neurons, brain cortex and hippocampus (Table S2; Query url).

The majority of the resulting interactions involved the Microtubule‐Associated Protein Tau (MAPT), including the Tau‐F reference sequence (UniProtKB: P10636‐8), its mutant variants and phosphorylated forms. Most interactions were identified using AP or proximity‐based methods. We categorised and analysed the prey molecules associated with each Tau form, aiming to uncover corresponding stable macromolecular complexes and evaluate their potential roles in Alzheimer's pathology.

Two additional contextual interactomes—the phosphorylated Tau interactome identified in the human brain [16] and the APP network from the human hippocampus [18]—were excluded from our analysis due to their limited network sizes, which were insufficient for robust complex enrichment analysis. The final list of interactions used in our study is provided in Table S3.

### 4.1 MAPT in Alzheimer's Disease

The MAPT plays a crucial role in the assembly and stabilisation of microtubules (MTs) by associating with them through its repeat domain (RD). The higher molecular mass isoforms, often referred to as ‘big tau’, are predominantly expressed in the peripheral nervous system. Alternative splicing of exon 10 gives rise to tau isoforms with three or four MT‐binding repeats in the MT‐binding domain (3R or 4R) [19]. This alternative splicing contributes to the diversity of tau isoforms, allowing for tissue‐specific expression patterns. Within the RD, two hexapeptide motifs are of particular importance as they are critical for tau aggregation [20]. The RD forms the structural core of disease‐associated aggregates and mutations within this domain underlie familial tauopathies [19, 21]. These tauopathies are characterised by the presence of abundant hyperphosphorylated filamentous tau inclusions [15, 22], particularly observed in neurodegenerative disorders such as AD.

It is noteworthy that distinct splice isoforms and mutant proteins often bind different partners to their canonical counterparts. This nuanced selectivity in interactions contributes to the intricate landscape of protein networks associated with AD.

The detailed curation model adopted by the International Molecular Exchange Consortium (IMEx) enables a comprehensive analysis of these differences. By providing a meticulous catalogue of interactions for splice isoforms, mutated proteins and their associated posttranslational modifications (PTMs), the IMEx datasets facilitate a deeper understanding of the dynamic interplay within the tissue‐specific interactome.

#### 4.1.1 Comparative Analysis of MAPT Interactomes

In our investigation, the interactomes of Tau‐F isoform (containing four MT‐binding repeats) and its two mutant variants (P301L rs63751273 and V337M rs63750570), known to increase tau aggregation in vitro, decrease MT assembly and decrease MT binding [19], were subjected to comparative analysis using Cytoscape.

**FIGURE 1:**
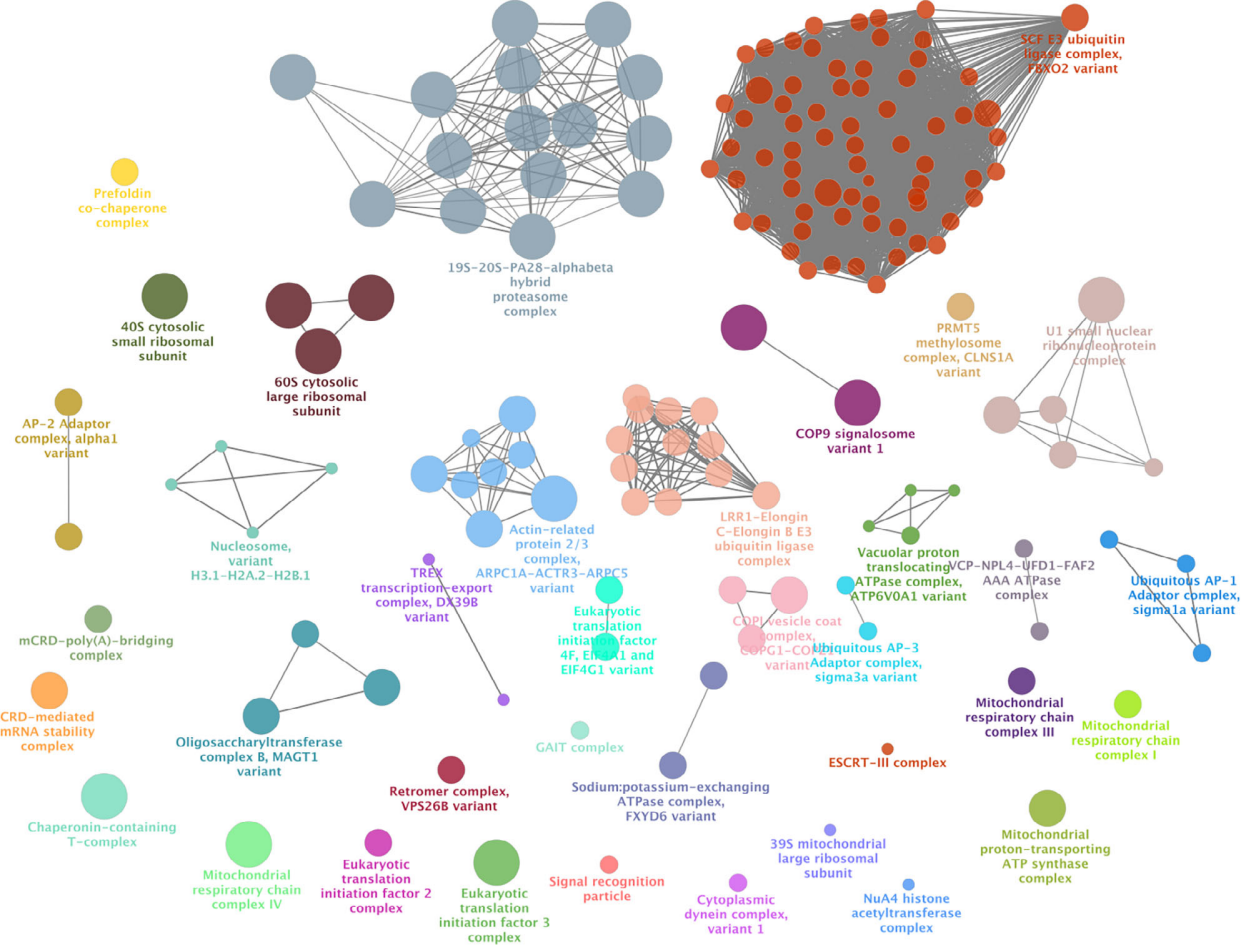
Enrichment analysis of high‐confident human Tau‐F proteins for Complex Portal complexes using ClueGO. SCF E3 ubiquitin ligases and the proteasomes are the prominent groups of complexes enriched, along with Elongin B E3 ubiquitin ligase complexes.

The normal Tau‐F network is characterised by the presence of a diverse array of complexes that play crucial roles in various cellular functions (Figure 1, Table 1). These include:

- *SCF E3 ubiquitin ligase complexes*: Involved in tagging proteins for degradation via the ubiquitin‐proteasome system, thus regulating various cellular processes, including cell cycle progression and signal transduction.
- *Elongin C‐elongin B E3 ubiquitin ligase complexes*: E3 ubiquitin ligase complexes that play a role in transcription elongation and protein degradation, crucial for maintaining protein quality control.
- *Proteasome family complexes*: Responsible for degrading unneeded or damaged proteins by proteolysis, a key component of cellular homeostasis and regulation.
- *Actin‐related protein 2/3 complex family complexes*: Critical for the regulation of the actin cytoskeleton, which is involved in various cellular processes, including cell motility, intracellular transport and cell division.
- *Sm complex and small nuclear ribonucleoprotein (snRNP) complex*: Involved in RNA splicing and the processing of pre‐mRNA, essential for the maturation and stability of mRNA.
- *Nucleosome complexes*: Fundamental units of chromatin, involved in DNA packaging and regulation of gene expression through epigenetic modifications.
- *Oligosaccharyltransferase complexes*: Key players in protein glycosylation, which is crucial for protein folding, stability and signalling.
- *VCP‐NPL4 AAA ATPases*: Involved in protein degradation, particularly in the dislocation of misfolded proteins from the endoplasmic reticulum (ER) to the cytosol for degradation.
- *Eukaryotic translation initiation factor complexes*: Essential for the initiation of translation, playing a critical role in the control of protein synthesis.
- *Neuronal AP‐3 adaptor complex*: Involved in the formation of vesicles that transport synaptic vesicle proteins, critical for synaptic function and neurotransmission.
- *Mitochondrial respiratory chain complexes I, III, IV*: Integral components of the electron transport chain, crucial for ATP production and overall cellular energy metabolism.
- *mRNA stabilisation complexes*: Play a role in regulating the stability and turnover of mRNA molecules, impacting gene expression and cellular response to environmental changes.

**TABLE 1:** Complex Portal complexes enriched for high‐confidence human Tau‐F interacting proteins.

ID	Term	Ontology source	Term *p* value	Term *p* value corrected with Bonferroni step down	Group *p* value	Group *p* value corrected with Bonferroni step down	GOLevels	GOGroups	% Associated genes	Nr. genes	Associated genes found
CPX:1076	mCRD‐poly(A)‐bridging complex	COMPLEXPORTAL_Complexes_07.06.2024	0.00	0.03	0.00	0.02	[−1]	Group00	80.00	4.00	[CSDE1, HNRNPD, PABPC1, SYNCRIP]
CPX:1080	CRD‐mediated mRNA stability complex	COMPLEXPORTAL_Complexes_07.06.2024	0.00	0.00	0.00	0.00	[−1]	Group01	100.00	5.00	[DHX9, HNRNPU, IGF2BP1, SYNCRIP, YBX1]
CPX:2476	GAIT complex	COMPLEXPORTAL_Complexes_07.06.2024	0.01	0.09	0.01	0.09	[−1]	Group02	75.00	3.00	[GAPDH, RPL13A, SYNCRIP]
CPX:2652	Signal recognition particle	COMPLEXPORTAL_Complexes_07.06.2024	0.01	0.09	0.01	0.09	[−1]	Group03	57.14	4.00	[SRP14, SRP68, SRP72, SRP9]
CPX:2716	Eukaryotic translation initiation Factor 2 complex	COMPLEXPORTAL_Complexes_07.06.2024	0.00	0.04	0.00	0.03	[−1]	Group04	100.00	3.00	[EIF2S1, EIF2S2, EIF2S3]
CPX:329	ESCRT‐III complex	COMPLEXPORTAL_Complexes_07.06.2024	0.07	0.48	0.07	0.48	[−1]	Group05	33.33	4.00	[CHMP1A, CHMP4B, CHMP5, CHMP6]
CPX:5025	Cytoplasmic dynein complex, Variant 1	COMPLEXPORTAL_Complexes_07.06.2024	0.00	0.05	0.00	0.05	[−1]	Group06	66.67	4.00	[DYNC1H1, DYNC1I2, DYNLL1, DYNLRB1]
CPX:7843	Retromer complex, VPS26B variant	COMPLEXPORTAL_Complexes_07.06.2024	0.00	0.04	0.00	0.03	[−1]	Group07	100.00	3.00	[VPS26B, VPS29, VPS35]
CPX:5223	40S cytosolic small ribosomal subunit	COMPLEXPORTAL_Complexes_07.06.2024	0.00	0.00	0.00	0.00	[−1]	Group08	100.00	30.00	[RACK1, RPS10, RPS11, RPS12, RPS13, RPS14, RPS15, RPS15A, RPS16, RPS17, RPS18, RPS19, RPS2, RPS20, RPS21, RPS23, RPS24, RPS25, RPS26, RPS27A, RPS28, RPS29, RPS3, RPS3A, RPS5, RPS6, RPS7, RPS8, RPS9, RPSA]
CPX:5226	39S mitochondrial large ribosomal subunit	COMPLEXPORTAL_Complexes_07.06.2024	0.84	1.00	0.84	0.84	[−1]	Group09	11.32	6.00	[MRPL16, MRPL34, MRPL40, MRPL46, MRPL48, MRPL9]
CPX:560	Mitochondrial respiratory chain Complex III	COMPLEXPORTAL_Complexes_07.06.2024	0.00	0.02	0.00	0.02	[−1]	Group10	54.55	6.00	[CYC1, UQCR10, UQCRB, UQCRC1, UQCRC2, UQCRH]
CPX:577	Mitochondrial respiratory chain Complex I	COMPLEXPORTAL_Complexes_07.06.2024	0.00	0.03	0.00	0.02	[−1]	Group11	31.82	14.00	[NDUFA10, NDUFA13, NDUFA5, NDUFA8, NDUFA9, NDUFAB1, NDUFB10, NDUFB3, NDUFB8, NDUFB9, NDUFS1, NDUFS3, NDUFS4, NDUFS5]
CPX:6030	Chaperonin‐containing T‐complex	COMPLEXPORTAL_Complexes_07.06.2024	0.00	0.00	0.00	0.00	[−1]	Group12	100.00	8.00	[CCT2, CCT3, CCT4, CCT5, CCT6A, CCT7, CCT8, TCP1]
CPX:6036	Eukaryotic translation initiation Factor 3 complex	COMPLEXPORTAL_Complexes_07.06.2024	0.00	0.00	0.00	0.00	[−1]	Group13	100.00	13.00	[EIF3A, EIF3B, EIF3C, EIF3D, EIF3E, EIF3F, EIF3G, EIF3H, EIF3I, EIF3J, EIF3K, EIF3L, EIF3M]
CPX:6123	Mitochondrial respiratory chain Complex IV	COMPLEXPORTAL_Complexes_07.06.2024	0.00	0.00	0.00	0.00	[−1]	Group14	78.57	11.00	[COX1, COX2, COX4I1, COX5A, COX5B, COX6A1, COX6B1, COX6C, COX7A2, COX7C, NDUFA4]
CPX:6149	Prefoldin co‐chaperone complex	COMPLEXPORTAL_Complexes_07.06.2024	0.00	0.01	0.00	0.01	[−1]	Group15	83.33	5.00	[PFDN1, PFDN2, PFDN5, PFDN6, VBP1]
CPX:6151	Mitochondrial proton‐transporting ATP synthase complex	COMPLEXPORTAL_Complexes_07.06.2024	0.00	0.00	0.00	0.00	[−1]	Group16	50.00	10.00	[ATP5F1A, ATP5F1B, ATP5F1C, ATP5F1D, ATP5ME, ATP5MG, ATP5PB, ATP5PD, ATP5PF, ATP5PO]
CPX:696	PRMT5 methylosome complex, CLNS1A variant	COMPLEXPORTAL_Complexes_07.06.2024	0.00	0.04	0.00	0.03	[−1]	Group17	100.00	3.00	[CLNS1A, PRMT5, WDR77]
CPX:978	NuA4 histone acetyltransferase complex	COMPLEXPORTAL_Complexes_07.06.2024	0.31	1.00	0.31	1.00	[−1]	Group18	21.05	4.00	[ACTB, MORF4L2, RUVBL1, RUVBL2]
CPX:1870	COP9 signalosome Variant 1	COMPLEXPORTAL_Complexes_07.06.2024	0.00	0.00	0.00	0.00	[−1]	Group19	100.00	8.00	[COPS2, COPS3, COPS4, COPS5, COPS6, COPS7A, COPS8, GPS1]
CPX:1871	COP9 signalosome Variant 2	COMPLEXPORTAL_Complexes_07.06.2024	0.00	0.00	0.00	0.00	[−1]	Group19	100.00	8.00	[COPS2, COPS3, COPS4, COPS5, COPS6, COPS7B, COPS8, GPS1]
CPX:5051	Ubiquitous AP‐3 adaptor complex, sigma3a variant	COMPLEXPORTAL_Complexes_07.06.2024	0.01	0.09	0.00	0.05	[−1]	Group20	75.00	3.00	[AP3D1, AP3M1, AP3S1]
CPX:5055	Neuronal AP‐3 adaptor complex, sigma3a variant	COMPLEXPORTAL_Complexes_07.06.2024	0.01	0.09	0.00	0.05	[−1]	Group20	75.00	3.00	[AP3B2, AP3D1, AP3S1]
CPX:5149	AP‐2 adaptor complex, alpha1 variant	COMPLEXPORTAL_Complexes_07.06.2024	0.00	0.01	0.00	0.00	[−1]	Group21	100.00	4.00	[AP2A1, AP2B1, AP2M1, AP2S1]
CPX:5150	AP‐2 adaptor complex, alpha2 variant	COMPLEXPORTAL_Complexes_07.06.2024	0.00	0.01	0.00	0.00	[−1]	Group21	100.00	4.00	[AP2A2, AP2B1, AP2M1, AP2S1]
CPX:2666	Eukaryotic translation initiation Factor 4F, EIF4A1 and EIF4G1 variant	COMPLEXPORTAL_Complexes_07.06.2024	0.00	0.04	0.00	0.01	[−1]	Group22	100.00	3.00	[EIF4A1, EIF4E, EIF4G1]
CPX:5634	Eukaryotic translation initiation Factor 4F, EIF4A2 and EIF4G1 variant	COMPLEXPORTAL_Complexes_07.06.2024	0.00	0.04	0.00	0.01	[−1]	Group22	100.00	3.00	[EIF4A2, EIF4E, EIF4G1]
CPX:2488	TREX transcription‐export complex, DX39B variant	COMPLEXPORTAL_Complexes_07.06.2024	0.47	1.00	0.50	1.00	[−1]	Group23	18.75	3.00	[ALYREF, CHTOP, ERH]
CPX:7261	TREX transcription‐export complex, DX39A variant	COMPLEXPORTAL_Complexes_07.06.2024	0.47	1.00	0.50	1.00	[−1]	Group23	18.75	3.00	[ALYREF, CHTOP, ERH]
CPX:8104	VCP‐NPL4‐UFD1‐FAF2 AAA ATPase complex	COMPLEXPORTAL_Complexes_07.06.2024	0.01	0.09	0.00	0.02	[−1]	Group24	75.00	3.00	[FAF2, UFD1, VCP]
CPX:8105	VCP‐NPL4‐UFD1‐UBXN1 AAA ATPase complex	COMPLEXPORTAL_Complexes_07.06.2024	0.01	0.09	0.00	0.02	[−1]	Group24	75.00	3.00	[UBXN1, UFD1, VCP]
CPX:8144	Sodium:potassium‐exchanging ATPase complex, FXYD6 variant	COMPLEXPORTAL_Complexes_07.06.2024	0.00	0.04	0.00	0.01	[−1]	Group25	100.00	3.00	[ATP1A1, ATP1B1, FXYD6]
CPX:8146	Sodium:potassium‐exchanging ATPase complex, FXYD7 variant	COMPLEXPORTAL_Complexes_07.06.2024	0.00	0.04	0.00	0.01	[−1]	Group25	100.00	3.00	[ATP1A1, ATP1B1, FXYD7]
CPX:5047	Ubiquitous AP‐1 adaptor complex, sigma1a variant	COMPLEXPORTAL_Complexes_07.06.2024	0.01	0.09	0.04	0.29	[−1]	Group26	75.00	3.00	[AP1B1, AP1G1, AP1M1]
CPX:5048	Ubiquitous AP‐1 adaptor complex, sigma1b variant	COMPLEXPORTAL_Complexes_07.06.2024	0.01	0.09	0.04	0.29	[−1]	Group26	75.00	3.00	[AP1B1, AP1G1, AP1M1]
CPX:5049	Ubiquitous AP‐1 adaptor complex, sigma1c variant	COMPLEXPORTAL_Complexes_07.06.2024	0.01	0.09	0.04	0.29	[−1]	Group26	75.00	3.00	[AP1B1, AP1G1, AP1M1]
CPX:7803	COPI vesicle coat complex, COPG1‐COPZ1 variant	COMPLEXPORTAL_Complexes_07.06.2024	0.00	0.00	0.00	0.01	[−1]	Group27	85.71	6.00	[ARCN1, COPA, COPB1, COPB2, COPG1, COPZ1]
CPX:7969	COPI vesicle coat complex, COPG2‐COPZ1 variant	COMPLEXPORTAL_Complexes_07.06.2024	0.00	0.02	0.00	0.01	[−1]	Group27	71.43	5.00	[ARCN1, COPA, COPB1, COPB2, COPZ1]
CPX:7970	COPI vesicle coat complex, COPG1‐COPZ2 variant	COMPLEXPORTAL_Complexes_07.06.2024	0.00	0.02	0.00	0.01	[−1]	Group27	71.43	5.00	[ARCN1, COPA, COPB1, COPB2, COPG1]
CPX:5183	60S cytosolic large ribosomal subunit	COMPLEXPORTAL_Complexes_07.06.2024	0.00	0.00	0.00	0.00	[−1]	Group28	84.78	39.00	[RPL10A, RPL11, RPL12, RPL13, RPL13A, RPL14, RPL15, RPL17, RPL18, RPL18A, RPL19, RPL21, RPL23, RPL23A, RPL24, RPL27, RPL27A, RPL28, RPL29, RPL30, RPL31, RPL32, RPL34, RPL35, RPL35A, RPL36, RPL37A, RPL38, RPL4, RPL5, RPL6, RPL7, RPL7A, RPL8, RPL9, RPLP0, RPLP1, RPLP2, UBA52]
CPX:7664	60S cytosolic large ribosomal subunit, testis‐specific variant	COMPLEXPORTAL_Complexes_07.06.2024	0.00	0.00	0.00	0.00	[−1]	Group28	84.78	39.00	[RPL10A, RPL11, RPL12, RPL13, RPL13A, RPL14, RPL15, RPL17, RPL18, RPL18A, RPL19, RPL21, RPL23, RPL23A, RPL24, RPL27, RPL27A, RPL28, RPL29, RPL30, RPL31, RPL32, RPL34, RPL35, RPL35A, RPL36, RPL37A, RPL38, RPL4, RPL5, RPL6, RPL7, RPL7A, RPL8, RPL9, RPLP0, RPLP1, RPLP2, UBA52]
CPX:7665	60S cytosolic large ribosomal subunit, striated muscle variant	COMPLEXPORTAL_Complexes_07.06.2024	0.00	0.00	0.00	0.00	[−1]	Group28	82.98	39.00	[RPL10A, RPL11, RPL12, RPL13, RPL13A, RPL14, RPL15, RPL17, RPL18, RPL18A, RPL19, RPL21, RPL23, RPL23A, RPL24, RPL27, RPL27A, RPL28, RPL29, RPL30, RPL31, RPL32, RPL34, RPL35, RPL35A, RPL36, RPL37A, RPL38, RPL4, RPL5, RPL6, RPL7, RPL7A, RPL8, RPL9, RPLP0, RPLP1, RPLP2, UBA52]
CPX:5621	Oligosaccharyltransferase Complex A	COMPLEXPORTAL_Complexes_07.06.2024	0.00	0.01	0.00	0.01	[−1]	Group29	66.67	6.00	[DAD1, DDOST, RPN1, RPN2, STT3A, TMEM258]
CPX:5622	Oligosaccharyltransferase Complex B, MAGT1 variant	COMPLEXPORTAL_Complexes_07.06.2024	0.00	0.00	0.00	0.01	[−1]	Group29	75.00	6.00	[DAD1, DDOST, RPN1, RPN2, STT3B, TMEM258]
CPX:8738	Oligosaccharyltransferase Complex B, TUCS3 variant	COMPLEXPORTAL_Complexes_07.06.2024	0.00	0.00	0.00	0.01	[−1]	Group29	75.00	6.00	[DAD1, DDOST, RPN1, RPN2, STT3B, TMEM258]
CPX:2556	Nucleosome, variant H3.1‐H2A.2‐H2B.1	COMPLEXPORTAL_Complexes_07.06.2024	1.00	1.00	0.61	1.00	[−1]	Group30	11.11	3.00	[H2AC4, H2BC11, H4‐16]
CPX:2564	Nucleosome, variant H3.1t‐H2A.2‐H2B.1	COMPLEXPORTAL_Complexes_07.06.2024	0.73	1.00	0.61	1.00	[−1]	Group30	16.67	3.00	[H2AC4, H2BC11, H4‐16]
CPX:5647	CENP‐A nucleosome complex	COMPLEXPORTAL_Complexes_07.06.2024	0.73	1.00	0.61	1.00	[−1]	Group30	16.67	3.00	[H2AC4, H2BC11, H4‐16]
CPX:5668	Nucleosome, variant H3.2‐H2A.2‐H2B.1	COMPLEXPORTAL_Complexes_07.06.2024	0.75	1.00	0.61	1.00	[−1]	Group30	15.00	3.00	[H2AC4, H2BC11, H4‐16]
CPX:2470	Vacuolar proton translocating ATPase complex, ATP6V0A1 variant	COMPLEXPORTAL_Complexes_07.06.2024	0.01	0.08	0.02	0.21	[−1]	Group31	50.00	5.00	[ATP6V0A1, ATP6V0C, ATP6V1A, ATP6V1D, ATP6V1H]
CPX:6904	Vacuolar proton translocating ATPase complex, ATP6V0A2 variant	COMPLEXPORTAL_Complexes_07.06.2024	0.04	0.29	0.02	0.21	[−1]	Group31	40.00	4.00	[ATP6V0C, ATP6V1A, ATP6V1D, ATP6V1H]
CPX:6905	Vacuolar proton translocating ATPase complex, ATP6V0A3 variant	COMPLEXPORTAL_Complexes_07.06.2024	0.04	0.29	0.02	0.21	[−1]	Group31	40.00	4.00	[ATP6V0C, ATP6V1A, ATP6V1D, ATP6V1H]
CPX:6912	Vacuolar proton translocating ATPase complex, ATP6V0A4 variant	COMPLEXPORTAL_Complexes_07.06.2024	0.04	0.29	0.02	0.21	[−1]	Group31	40.00	4.00	[ATP6V0C, ATP6V1A, ATP6V1D, ATP6V1H]
CPX:2391	U4/U6.U5 small nuclear ribonucleoprotein complex	COMPLEXPORTAL_Complexes_07.06.2024	0.00	0.03	0.00	0.01	[−1]	Group32	34.38	11.00	[LSM3, PRPF31, SART1, SNRPA, SNRPD1, SNRPD2, SNRPD3, SNRPE, SNRPF, SNRPG, SNU13]
CPX:2392	U1 small nuclear ribonucleoprotein complex	COMPLEXPORTAL_Complexes_07.06.2024	0.00	0.00	0.00	0.01	[−1]	Group32	90.00	9.00	[SNRNP70, SNRPA, SNRPC, SNRPD1, SNRPD2, SNRPD3, SNRPE, SNRPF, SNRPG]
CPX:2539	U2 small nuclear ribonucleoprotein complex	COMPLEXPORTAL_Complexes_07.06.2024	0.00	0.02	0.00	0.01	[−1]	Group32	40.00	10.00	[HTATSF1, SF3A3, SF3B3, SF3B6, SNRPD1, SNRPD2, SNRPD3, SNRPE, SNRPF, SNRPG]
CPX:2705	U7 small nuclear ribonucleoprotein complex	COMPLEXPORTAL_Complexes_07.06.2024	0.01	0.09	0.00	0.01	[−1]	Group32	57.14	4.00	[SNRPD3, SNRPE, SNRPF, SNRPG]
CPX:6033	Sm complex	COMPLEXPORTAL_Complexes_07.06.2024	0.00	0.00	0.00	0.01	[−1]	Group32	85.71	6.00	[SNRPD1, SNRPD2, SNRPD3, SNRPE, SNRPF, SNRPG]
CPX:2490	Actin‐related protein 2/3 complex, ARPC1A‐ACTR3B‐ARPC5 variant	COMPLEXPORTAL_Complexes_07.06.2024	0.00	0.00	0.00	0.00	[−1]	Group33	85.71	6.00	[ACTR2, ARPC1A, ARPC2, ARPC3, ARPC4, ARPC5]
CPX:2579	Actin‐related protein 2/3 complex, ARPC1B‐ACTR3‐ARPC5 variant	COMPLEXPORTAL_Complexes_07.06.2024	0.00	0.00	0.00	0.00	[−1]	Group33	85.71	6.00	[ACTR2, ACTR3, ARPC2, ARPC3, ARPC4, ARPC5]
CPX:2580	Actin‐related protein 2/3 complex, ARPC1B‐ACTR3B‐ARPC5L variant	COMPLEXPORTAL_Complexes_07.06.2024	0.01	0.09	0.00	0.00	[−1]	Group33	57.14	4.00	[ACTR2, ARPC2, ARPC3, ARPC4]
CPX:2583	Actin‐related protein 2/3 complex, ARPC1B‐ACTR3B‐ARPC5 variant	COMPLEXPORTAL_Complexes_07.06.2024	0.00	0.02	0.00	0.00	[−1]	Group33	71.43	5.00	[ACTR2, ARPC2, ARPC3, ARPC4, ARPC5]
CPX:2586	Actin‐related protein 2/3 complex, ARPC1A‐ACTR3‐ARPC5 variant	COMPLEXPORTAL_Complexes_07.06.2024	0.00	0.00	0.00	0.00	[−1]	Group33	100.00	7.00	[ACTR2, ACTR3, ARPC1A, ARPC2, ARPC3, ARPC4, ARPC5]
CPX:2592	Actin‐related protein 2/3 complex, ARPC1A‐ACTR3‐ARPC5L variant	COMPLEXPORTAL_Complexes_07.06.2024	0.00	0.00	0.00	0.00	[−1]	Group33	85.71	6.00	[ACTR2, ACTR3, ARPC1A, ARPC2, ARPC3, ARPC4]
CPX:2663	Actin‐related protein 2/3 complex, ARPC1B‐ACTR3‐ARPC5L variant	COMPLEXPORTAL_Complexes_07.06.2024	0.00	0.02	0.00	0.00	[−1]	Group33	71.43	5.00	[ACTR2, ACTR3, ARPC2, ARPC3, ARPC4]
CPX:2668	Actin‐related protein 2/3 complex, ARPC1B‐ACTR3B‐ARPC5L variant	COMPLEXPORTAL_Complexes_07.06.2024	0.00	0.02	0.00	0.00	[−1]	Group33	71.43	5.00	[ACTR2, ARPC1A, ARPC2, ARPC3, ARPC4]
CPX:2214	LRR1‐Elongin C‐Elongin B E3 ubiquitin ligase complex	COMPLEXPORTAL_Complexes_07.06.2024	0.00	0.03	0.26	1.00	[−1]	Group34	80.00	4.00	[CUL2, ELOB, ELOC, RBX1]
CPX:2217	FEM1A‐Elongin C‐Elongin B E3 ubiquitin ligase complex	COMPLEXPORTAL_Complexes_07.06.2024	0.00	0.03	0.26	1.00	[−1]	Group34	80.00	4.00	[CUL2, ELOB, ELOC, RBX1]
CPX:2218	FEB1B‐Elongin C‐Elongin B E3 ubiquitin ligase complex	COMPLEXPORTAL_Complexes_07.06.2024	0.00	0.03	0.26	1.00	[−1]	Group34	80.00	4.00	[CUL2, ELOB, ELOC, RBX1]
CPX:2219	FEB1C‐Elongin C‐Elongin B E3 ubiquitin ligase complex	COMPLEXPORTAL_Complexes_07.06.2024	0.00	0.03	0.26	1.00	[−1]	Group34	80.00	4.00	[CUL2, ELOB, ELOC, RBX1]
CPX:2220	ZYG11B‐Elongin C‐Elongin B E3 ubiquitin ligase complex	COMPLEXPORTAL_Complexes_07.06.2024	0.00	0.03	0.26	1.00	[−1]	Group34	80.00	4.00	[CUL2, ELOB, ELOC, RBX1]
CPX:2221	APPBP2‐Elongin C‐Elongin B E3 ubiquitin ligase complex	COMPLEXPORTAL_Complexes_07.06.2024	0.00	0.03	0.26	1.00	[−1]	Group34	80.00	4.00	[CUL2, ELOB, ELOC, RBX1]
CPX:2222	ZER1‐Elongin C‐Elongin B E3 ubiquitin ligase complex	COMPLEXPORTAL_Complexes_07.06.2024	0.00	0.03	0.26	1.00	[−1]	Group34	80.00	4.00	[CUL2, ELOB, ELOC, RBX1]
CPX:2223	KLHDC10‐Elongin C‐Elongin B E3 ubiquitin ligase complex	COMPLEXPORTAL_Complexes_07.06.2024	0.00	0.03	0.26	1.00	[−1]	Group34	80.00	4.00	[CUL2, ELOB, ELOC, RBX1]
CPX:2226	KLHDC2‐Elongin C‐Elongin B E3 ubiquitin ligase complex	COMPLEXPORTAL_Complexes_07.06.2024	0.00	0.03	0.26	1.00	[−1]	Group34	80.00	4.00	[CUL2, ELOB, ELOC, RBX1]
CPX:2228	KLHDC3‐Elongin C‐Elongin B E3 ubiquitin ligase complex	COMPLEXPORTAL_Complexes_07.06.2024	0.00	0.03	0.26	1.00	[−1]	Group34	80.00	4.00	[CUL2, ELOB, ELOC, RBX1]
CPX:2229	PRAME‐Elongin C‐Elongin B E3 ubiquitin ligase complex	COMPLEXPORTAL_Complexes_07.06.2024	0.00	0.03	0.26	1.00	[−1]	Group34	80.00	4.00	[CUL2, ELOB, ELOC, RBX1]
CPX:2250	VHL‐Elongin C‐Elongin B E3 ubiquitin ligase complex	COMPLEXPORTAL_Complexes_07.06.2024	0.00	0.03	0.26	1.00	[−1]	Group34	80.00	4.00	[CUL2, ELOB, ELOC, RBX1]
CPX:5993	26S proteasome complex	COMPLEXPORTAL_Complexes_07.06.2024	0.00	0.00	0.00	0.00	[−1]	Group35	96.97	32.00	[ADRM1, PSMA1, PSMA2, PSMA3, PSMA4, PSMA5, PSMA6, PSMA7, PSMB1, PSMB2, PSMB3, PSMB4, PSMB5, PSMB6, PSMB7, PSMC1, PSMC2, PSMC3, PSMC4, PSMC5, PSMC6, PSMD1, PSMD11, PSMD12, PSMD13, PSMD14, PSMD2, PSMD3, PSMD4, PSMD6, PSMD7, PSMD8]
CPX:8806	20S proteasome complex	COMPLEXPORTAL_Complexes_07.06.2024	0.00	0.00	0.00	0.00	[−1]	Group35	100.00	14.00	[PSMA1, PSMA2, PSMA3, PSMA4, PSMA5, PSMA6, PSMA7, PSMB1, PSMB2, PSMB3, PSMB4, PSMB5, PSMB6, PSMB7]
CPX:8841	PA200‐20S single‐capped proteasome	COMPLEXPORTAL_Complexes_07.06.2024	0.00	0.00	0.00	0.00	[−1]	Group35	93.33	14.00	[PSMA1, PSMA2, PSMA3, PSMA4, PSMA5, PSMA6, PSMA7, PSMB1, PSMB2, PSMB3, PSMB4, PSMB5, PSMB6, PSMB7]
CPX:8842	PA28‐alphabeta double‐capped 20S proteasome complex	COMPLEXPORTAL_Complexes_07.06.2024	0.00	0.00	0.00	0.00	[−1]	Group35	100.00	16.00	[PSMA1, PSMA2, PSMA3, PSMA4, PSMA5, PSMA6, PSMA7, PSMB1, PSMB2, PSMB3, PSMB4, PSMB5, PSMB6, PSMB7, PSME1, PSME2]
CPX:8964	19S proteasome regulatory complex	COMPLEXPORTAL_Complexes_07.06.2024	0.00	0.00	0.00	0.00	[−1]	Group35	94.74	18.00	[ADRM1, PSMC1, PSMC2, PSMC3, PSMC4, PSMC5, PSMC6, PSMD1, PSMD11, PSMD12, PSMD13, PSMD14, PSMD2, PSMD3, PSMD4, PSMD6, PSMD7, PSMD8]
CPX:9001	PA28‐gamma single‐capped 20S proteasome complex	COMPLEXPORTAL_Complexes_07.06.2024	0.00	0.00	0.00	0.00	[−1]	Group35	93.33	14.00	[PSMA1, PSMA2, PSMA3, PSMA4, PSMA5, PSMA6, PSMA7, PSMB1, PSMB2, PSMB3, PSMB4, PSMB5, PSMB6, PSMB7]
CPX:9002	PA28‐alphabeta single‐capped 20S proteasome complex	COMPLEXPORTAL_Complexes_07.06.2024	0.00	0.00	0.00	0.00	[−1]	Group35	100.00	16.00	[PSMA1, PSMA2, PSMA3, PSMA4, PSMA5, PSMA6, PSMA7, PSMB1, PSMB2, PSMB3, PSMB4, PSMB5, PSMB6, PSMB7, PSME1, PSME2]
CPX:9003	20S immunoproteasome complex	COMPLEXPORTAL_Complexes_07.06.2024	0.00	0.00	0.00	0.00	[−1]	Group35	78.57	11.00	[PSMA1, PSMA2, PSMA3, PSMA4, PSMA5, PSMA6, PSMA7, PSMB1, PSMB2, PSMB3, PSMB4]
CPX:9004	20S thymoproteasome complex	COMPLEXPORTAL_Complexes_07.06.2024	0.00	0.00	0.00	0.00	[−1]	Group35	78.57	11.00	[PSMA1, PSMA2, PSMA3, PSMA4, PSMA5, PSMA6, PSMA7, PSMB1, PSMB2, PSMB3, PSMB4]
CPX:9021	20S spermatoproteasome complex	COMPLEXPORTAL_Complexes_07.06.2024	0.00	0.00	0.00	0.00	[−1]	Group35	92.86	13.00	[PSMA1, PSMA2, PSMA3, PSMA4, PSMA5, PSMA6, PSMB1, PSMB2, PSMB3, PSMB4, PSMB5, PSMB6, PSMB7]
CPX:9022	PA28‐gamma double‐capped 20S proteasome complex	COMPLEXPORTAL_Complexes_07.06.2024	0.00	0.00	0.00	0.00	[−1]	Group35	93.33	14.00	[PSMA1, PSMA2, PSMA3, PSMA4, PSMA5, PSMA6, PSMA7, PSMB1, PSMB2, PSMB3, PSMB4, PSMB5, PSMB6, PSMB7]
CPX:9063	PA200‐20S‐PA200 double‐capped proteasome complex	COMPLEXPORTAL_Complexes_07.06.2024	0.00	0.00	0.00	0.00	[−1]	Group35	93.33	14.00	[PSMA1, PSMA2, PSMA3, PSMA4, PSMA5, PSMA6, PSMA7, PSMB1, PSMB2, PSMB3, PSMB4, PSMB5, PSMB6, PSMB7]
CPX:9082	19S‐20S‐PA28‐alphabeta hybrid proteasome complex	COMPLEXPORTAL_Complexes_07.06.2024	0.00	0.00	0.00	0.00	[−1]	Group35	97.14	34.00	[ADRM1, PSMA1, PSMA2, PSMA3, PSMA4, PSMA5, PSMA6, PSMA7, PSMB1, PSMB2, PSMB3, PSMB4, PSMB5, PSMB6, PSMB7, PSMC1, PSMC2, PSMC3, PSMC4, PSMC5, PSMC6, PSMD1, PSMD11, PSMD12, PSMD13, PSMD14, PSMD2, PSMD3, PSMD4, PSMD6, PSMD7, PSMD8, PSME1, PSME2]
CPX:9085	19S‐20S‐PA28‐gamma hybrid proteasome complex	COMPLEXPORTAL_Complexes_07.06.2024	0.00	0.00	0.00	0.00	[−1]	Group35	94.12	32.00	[ADRM1, PSMA1, PSMA2, PSMA3, PSMA4, PSMA5, PSMA6, PSMA7, PSMB1, PSMB2, PSMB3, PSMB4, PSMB5, PSMB6, PSMB7, PSMC1, PSMC2, PSMC3, PSMC4, PSMC5, PSMC6, PSMD1, PSMD11, PSMD12, PSMD13, PSMD14, PSMD2, PSMD3, PSMD4, PSMD6, PSMD7, PSMD8]
CPX:9086	30S proteasome complex	COMPLEXPORTAL_Complexes_07.06.2024	0.00	0.00	0.00	0.00	[−1]	Group35	96.97	32.00	[ADRM1, PSMA1, PSMA2, PSMA3, PSMA4, PSMA5, PSMA6, PSMA7, PSMB1, PSMB2, PSMB3, PSMB4, PSMB5, PSMB6, PSMB7, PSMC1, PSMC2, PSMC3, PSMC4, PSMC5, PSMC6, PSMD1, PSMD11, PSMD12, PSMD13, PSMD14, PSMD2, PSMD3, PSMD4, PSMD6, PSMD7, PSMD8]
CPX:2239	SCF E3 ubiquitin ligase complex, FBXL5 variant	COMPLEXPORTAL_Complexes_07.06.2024	0.01	0.09	0.12	0.69	[−1]	Group36	75.00	3.00	[CUL1, RBX1, SKP1]
CPX:2241	SCF E3 ubiquitin ligase complex, FBXL13 variant	COMPLEXPORTAL_Complexes_07.06.2024	0.01	0.09	0.12	0.69	[−1]	Group36	75.00	3.00	[CUL1, RBX1, SKP1]
CPX:2319	SCF E3 ubiquitin ligase complex, FBXL14 variant	COMPLEXPORTAL_Complexes_07.06.2024	0.01	0.09	0.12	0.69	[−1]	Group36	75.00	3.00	[CUL1, RBX1, SKP1]
CPX:2343	SCF E3 ubiquitin ligase complex, FBXL16 variant	COMPLEXPORTAL_Complexes_07.06.2024	0.01	0.09	0.12	0.69	[−1]	Group36	75.00	3.00	[CUL1, RBX1, SKP1]
CPX:2365	SCF E3 ubiquitin ligase complex, BTRC variant	COMPLEXPORTAL_Complexes_07.06.2024	0.01	0.09	0.12	0.69	[−1]	Group36	75.00	3.00	[CUL1, RBX1, SKP1]
CPX:2438	SCF E3 ubiquitin ligase complex, FBXL8 variant	COMPLEXPORTAL_Complexes_07.06.2024	0.01	0.09	0.12	0.69	[−1]	Group36	75.00	3.00	[CUL1, RBX1, SKP1]
CPX:2489	SCF E3 ubiquitin ligase complex, FBXL22 variant	COMPLEXPORTAL_Complexes_07.06.2024	0.01	0.09	0.12	0.69	[−1]	Group36	75.00	3.00	[CUL1, RBX1, SKP1]
CPX:2492	SCF E3 ubiquitin ligase complex, FBXL15 variant	COMPLEXPORTAL_Complexes_07.06.2024	0.01	0.09	0.12	0.69	[−1]	Group36	75.00	3.00	[CUL1, RBX1, SKP1]
CPX:2512	SCF E3 ubiquitin ligase complex, FBXL4 variant	COMPLEXPORTAL_Complexes_07.06.2024	0.01	0.09	0.12	0.69	[−1]	Group36	75.00	3.00	[CUL1, RBX1, SKP1]
CPX:2516	SCF E3 ubiquitin ligase complex, FBXL6 variant	COMPLEXPORTAL_Complexes_07.06.2024	0.01	0.09	0.12	0.69	[−1]	Group36	75.00	3.00	[CUL1, RBX1, SKP1]
CPX:2538	SCF E3 ubiquitin ligase complex, KDM2A variant	COMPLEXPORTAL_Complexes_07.06.2024	0.01	0.09	0.12	0.69	[−1]	Group36	75.00	3.00	[CUL1, RBX1, SKP1]
CPX:2553	SCF E3 ubiquitin ligase complex, FBXL18 variant	COMPLEXPORTAL_Complexes_07.06.2024	0.00	0.04	0.12	0.69	[−1]	Group36	100.00	3.00	[CUL1, RBX1, SKP1]
CPX:2554	SCF E3 ubiquitin ligase complex, FBXL19 variant	COMPLEXPORTAL_Complexes_07.06.2024	0.01	0.09	0.12	0.69	[−1]	Group36	75.00	3.00	[CUL1, RBX1, SKP1]
CPX:2658	SCF E3 ubiquitin ligase complex, FBXL12 variant	COMPLEXPORTAL_Complexes_07.06.2024	0.01	0.09	0.12	0.69	[−1]	Group36	75.00	3.00	[CUL1, RBX1, SKP1]
CPX:2683	SCF E3 ubiquitin ligase complex, FBXL7 variant	COMPLEXPORTAL_Complexes_07.06.2024	0.01	0.09	0.12	0.69	[−1]	Group36	75.00	3.00	[CUL1, RBX1, SKP1]
CPX:2748	SCF E3 ubiquitin ligase complex, FBXL21 variant	COMPLEXPORTAL_Complexes_07.06.2024	0.00	0.04	0.12	0.69	[−1]	Group36	100.00	3.00	[CUL1, RBX1, SKP1]
CPX:2773	SCF E3 ubiquitin ligase complex, FBXL17 variant	COMPLEXPORTAL_Complexes_07.06.2024	0.01	0.09	0.12	0.69	[−1]	Group36	75.00	3.00	[CUL1, RBX1, SKP1]
CPX:2832	SCF E3 ubiquitin ligase complex, KDM2B variant	COMPLEXPORTAL_Complexes_07.06.2024	0.01	0.09	0.12	0.69	[−1]	Group36	75.00	3.00	[CUL1, RBX1, SKP1]
CPX:2873	SCF E3 ubiquitin ligase complex, FBXL20 variant	COMPLEXPORTAL_Complexes_07.06.2024	0.01	0.09	0.12	0.69	[−1]	Group36	75.00	3.00	[CUL1, RBX1, SKP1]
CPX:3291	SCF E3 ubiquitin ligase complex, FBXL3 variant	COMPLEXPORTAL_Complexes_07.06.2024	0.01	0.09	0.12	0.69	[−1]	Group36	75.00	3.00	[CUL1, RBX1, SKP1]
CPX:3292	SCF E3 ubiquitin ligase complex, FBXL2 variant	COMPLEXPORTAL_Complexes_07.06.2024	0.01	0.09	0.12	0.69	[−1]	Group36	75.00	3.00	[CUL1, RBX1, SKP1]
CPX:3295	SCF E3 ubiquitin ligase complex, SKP2 variant	COMPLEXPORTAL_Complexes_07.06.2024	0.01	0.09	0.12	0.69	[−1]	Group36	75.00	3.00	[CUL1, RBX1, SKP1]
CPX:7747	SCF E3 ubiquitin ligase complex, FBXW2 variant	COMPLEXPORTAL_Complexes_07.06.2024	0.01	0.09	0.12	0.69	[−1]	Group36	75.00	3.00	[CUL1, RBX1, SKP1]
CPX:7761	SCF E3 ubiquitin ligase complex, FBXW4 variant	COMPLEXPORTAL_Complexes_07.06.2024	0.01	0.09	0.12	0.69	[−1]	Group36	75.00	3.00	[CUL1, RBX1, SKP1]
CPX:7762	SCF E3 ubiquitin ligase complex, FBXW5 variant	COMPLEXPORTAL_Complexes_07.06.2024	0.01	0.09	0.12	0.69	[−1]	Group36	75.00	3.00	[CUL1, RBX1, SKP1]
CPX:7763	SCF E3 ubiquitin ligase complex, FBXW7 variant	COMPLEXPORTAL_Complexes_07.06.2024	0.01	0.09	0.12	0.69	[−1]	Group36	75.00	3.00	[CUL1, RBX1, SKP1]
CPX:7784	SCF E3 ubiquitin ligase complex, FBXW8‐CUL7 variant	COMPLEXPORTAL_Complexes_07.06.2024	0.02	0.18	0.12	0.69	[−1]	Group36	60.00	3.00	[CUL1, RBX1, SKP1]
CPX:7785	SCF E3 ubiquitin ligase complex, FBXW9 variant	COMPLEXPORTAL_Complexes_07.06.2024	0.01	0.09	0.12	0.69	[−1]	Group36	75.00	3.00	[CUL1, RBX1, SKP1]
CPX:7786	SCF E3 ubiquitin ligase complex, FBXW10 variant	COMPLEXPORTAL_Complexes_07.06.2024	0.01	0.09	0.12	0.69	[−1]	Group36	75.00	3.00	[CUL1, RBX1, SKP1]
CPX:7821	SCF E3 ubiquitin ligase complex, FBXW11 variant	COMPLEXPORTAL_Complexes_07.06.2024	0.01	0.09	0.12	0.69	[−1]	Group36	75.00	3.00	[CUL1, RBX1, SKP1]
CPX:7822	SCF E3 ubiquitin ligase complex, FBXW12 variant	COMPLEXPORTAL_Complexes_07.06.2024	0.01	0.09	0.12	0.69	[−1]	Group36	75.00	3.00	[CUL1, RBX1, SKP1]
CPX:7846	SCF E3 ubiquitin ligase complex, CCNF variant	COMPLEXPORTAL_Complexes_07.06.2024	0.01	0.09	0.12	0.69	[−1]	Group36	75.00	3.00	[CUL1, RBX1, SKP1]
CPX:7847	SCF E3 ubiquitin ligase complex, FBXO2 variant	COMPLEXPORTAL_Complexes_07.06.2024	0.00	0.01	0.12	0.69	[−1]	Group36	100.00	4.00	[CUL1, FBXO2, RBX1, SKP1]
CPX:7881	SCF E3 ubiquitin ligase complex, FBXO3 variant	COMPLEXPORTAL_Complexes_07.06.2024	0.01	0.09	0.12	0.69	[−1]	Group36	75.00	3.00	[CUL1, RBX1, SKP1]
CPX:7882	SCF E3 ubiquitin ligase complex, FBXO4 variant	COMPLEXPORTAL_Complexes_07.06.2024	0.01	0.09	0.12	0.69	[−1]	Group36	75.00	3.00	[CUL1, RBX1, SKP1]
CPX:7904	SCF E3 ubiquitin ligase complex, FBXO5 variant	COMPLEXPORTAL_Complexes_07.06.2024	0.01	0.09	0.12	0.69	[−1]	Group36	75.00	3.00	[CUL1, RBX1, SKP1]
CPX:7905	SCF E3 ubiquitin ligase complex, FBXO6 variant	COMPLEXPORTAL_Complexes_07.06.2024	0.01	0.09	0.12	0.69	[−1]	Group36	75.00	3.00	[CUL1, RBX1, SKP1]
CPX:7906	SCF E3 ubiquitin ligase complex, FBXO7 variant	COMPLEXPORTAL_Complexes_07.06.2024	0.01	0.09	0.12	0.69	[−1]	Group36	75.00	3.00	[CUL1, RBX1, SKP1]
CPX:7921	SCF E3 ubiquitin ligase complex, FBXO8 variant	COMPLEXPORTAL_Complexes_07.06.2024	0.01	0.09	0.12	0.69	[−1]	Group36	75.00	3.00	[CUL1, RBX1, SKP1]
CPX:7922	SCF E3 ubiquitin ligase complex, FBXO9 variant	COMPLEXPORTAL_Complexes_07.06.2024	0.01	0.09	0.12	0.69	[−1]	Group36	75.00	3.00	[CUL1, RBX1, SKP1]
CPX:7923	SCF E3 ubiquitin ligase complex, FBXO10 variant	COMPLEXPORTAL_Complexes_07.06.2024	0.01	0.09	0.12	0.69	[−1]	Group36	75.00	3.00	[CUL1, RBX1, SKP1]
CPX:7924	SCF E3 ubiquitin ligase complex, FBXO11 variant	COMPLEXPORTAL_Complexes_07.06.2024	0.01	0.09	0.12	0.69	[−1]	Group36	75.00	3.00	[CUL1, RBX1, SKP1]
CPX:7925	SCF E3 ubiquitin ligase complex, FBXO15 variant	COMPLEXPORTAL_Complexes_07.06.2024	0.01	0.09	0.12	0.69	[−1]	Group36	75.00	3.00	[CUL1, RBX1, SKP1]
CPX:7926	SCF E3 ubiquitin ligase complex, FBXO16 variant	COMPLEXPORTAL_Complexes_07.06.2024	0.01	0.09	0.12	0.69	[−1]	Group36	75.00	3.00	[CUL1, RBX1, SKP1]
CPX:7927	SCF E3 ubiquitin ligase complex, FBXO17 variant	COMPLEXPORTAL_Complexes_07.06.2024	0.01	0.09	0.12	0.69	[−1]	Group36	75.00	3.00	[CUL1, RBX1, SKP1]
CPX:7928	SCF E3 ubiquitin ligase complex, FBH1 variant	COMPLEXPORTAL_Complexes_07.06.2024	0.01	0.09	0.12	0.69	[−1]	Group36	75.00	3.00	[CUL1, RBX1, SKP1]
CPX:7929	SCF E3 ubiquitin ligase complex, LMO7 variant	COMPLEXPORTAL_Complexes_07.06.2024	0.01	0.09	0.12	0.69	[−1]	Group36	75.00	3.00	[CUL1, RBX1, SKP1]
CPX:7930	SCF E3 ubiquitin ligase complex, FBXO21 variant	COMPLEXPORTAL_Complexes_07.06.2024	0.00	0.01	0.12	0.69	[−1]	Group36	100.00	4.00	[CUL1, FBXO21, RBX1, SKP1]
CPX:7962	SCF E3 ubiquitin ligase complex, FBXO22 variant	COMPLEXPORTAL_Complexes_07.06.2024	0.01	0.09	0.12	0.69	[−1]	Group36	75.00	3.00	[CUL1, RBX1, SKP1]
CPX:7963	SCF E3 ubiquitin ligase complex, FBXO24 variant	COMPLEXPORTAL_Complexes_07.06.2024	0.01	0.09	0.12	0.69	[−1]	Group36	75.00	3.00	[CUL1, RBX1, SKP1]
CPX:7965	SCF E3 ubiquitin ligase complex, FBXO25 variant	COMPLEXPORTAL_Complexes_07.06.2024	0.01	0.09	0.12	0.69	[−1]	Group36	75.00	3.00	[CUL1, RBX1, SKP1]
CPX:7966	SCF E3 ubiquitin ligase complex, FBXO27 variant	COMPLEXPORTAL_Complexes_07.06.2024	0.01	0.09	0.12	0.69	[−1]	Group36	75.00	3.00	[CUL1, RBX1, SKP1]
CPX:7967	SCF E3 ubiquitin ligase complex, FBXO28 variant	COMPLEXPORTAL_Complexes_07.06.2024	0.01	0.09	0.12	0.69	[−1]	Group36	75.00	3.00	[CUL1, RBX1, SKP1]
CPX:7968	SCF E3 ubiquitin ligase complex, FBXO30 variant	COMPLEXPORTAL_Complexes_07.06.2024	0.01	0.09	0.12	0.69	[−1]	Group36	75.00	3.00	[CUL1, RBX1, SKP1]
CPX:7971	SCF E3 ubiquitin ligase complex, FBXO31 variant	COMPLEXPORTAL_Complexes_07.06.2024	0.01	0.09	0.12	0.69	[−1]	Group36	75.00	3.00	[CUL1, RBX1, SKP1]
CPX:7972	SCF E3 ubiquitin ligase complex, FBXO32 variant	COMPLEXPORTAL_Complexes_07.06.2024	0.01	0.09	0.12	0.69	[−1]	Group36	75.00	3.00	[CUL1, RBX1, SKP1]
CPX:7973	SCF E3 ubiquitin ligase complex, FBXO33 variant	COMPLEXPORTAL_Complexes_07.06.2024	0.01	0.09	0.12	0.69	[−1]	Group36	75.00	3.00	[CUL1, RBX1, SKP1]
CPX:7975	SCF E3 ubiquitin ligase complex, FBXO34 variant	COMPLEXPORTAL_Complexes_07.06.2024	0.01	0.09	0.12	0.69	[−1]	Group36	75.00	3.00	[CUL1, RBX1, SKP1]
CPX:7976	SCF E3 ubiquitin ligase complex, FBXO36 variant	COMPLEXPORTAL_Complexes_07.06.2024	0.01	0.09	0.12	0.69	[−1]	Group36	75.00	3.00	[CUL1, RBX1, SKP1]
CPX:7977	SCF E3 ubiquitin ligase complex, FBXO38 variant	COMPLEXPORTAL_Complexes_07.06.2024	0.01	0.09	0.12	0.69	[−1]	Group36	75.00	3.00	[CUL1, RBX1, SKP1]
CPX:7979	SCF E3 ubiquitin ligase complex, FBXO39 variant	COMPLEXPORTAL_Complexes_07.06.2024	0.01	0.09	0.12	0.69	[−1]	Group36	75.00	3.00	[CUL1, RBX1, SKP1]
CPX:7981	SCF E3 ubiquitin ligase complex, FBXO40 variant	COMPLEXPORTAL_Complexes_07.06.2024	0.01	0.09	0.12	0.69	[−1]	Group36	75.00	3.00	[CUL1, RBX1, SKP1]
CPX:7982	SCF E3 ubiquitin ligase complex, FBXO41 variant	COMPLEXPORTAL_Complexes_07.06.2024	0.01	0.09	0.12	0.69	[−1]	Group36	75.00	3.00	[CUL1, RBX1, SKP1]
CPX:7983	SCF E3 ubiquitin ligase complex, FBXO42 variant	COMPLEXPORTAL_Complexes_07.06.2024	0.01	0.09	0.12	0.69	[−1]	Group36	75.00	3.00	[CUL1, RBX1, SKP1]
CPX:8002	SCF E3 ubiquitin ligase complex, FBXO43 variant	COMPLEXPORTAL_Complexes_07.06.2024	0.01	0.09	0.12	0.69	[−1]	Group36	75.00	3.00	[CUL1, RBX1, SKP1]
CPX:8003	SCF E3 ubiquitin ligase complex, FBXO44 variant	COMPLEXPORTAL_Complexes_07.06.2024	0.01	0.09	0.12	0.69	[−1]	Group36	75.00	3.00	[CUL1, RBX1, SKP1]
CPX:8005	SCF E3 ubiquitin ligase complex, FBXO46 variant	COMPLEXPORTAL_Complexes_07.06.2024	0.01	0.09	0.12	0.69	[−1]	Group36	75.00	3.00	[CUL1, RBX1, SKP1]
CPX:8006	SCF E3 ubiquitin ligase complex, FBXO47 variant	COMPLEXPORTAL_Complexes_07.06.2024	0.01	0.09	0.12	0.69	[−1]	Group36	75.00	3.00	[CUL1, RBX1, SKP1]
CPX:8108	SCF E3 ubiquitin ligase complex, TSPAN17 variant	COMPLEXPORTAL_Complexes_07.06.2024	0.01	0.09	0.12	0.69	[−1]	Group36	75.00	3.00	[CUL1, RBX1, SKP1]

##### 4.1.1.1 Functional Implications of Specific Complexes in MAPT Interactomes

Within the Tau‐F interactome, the presence of critical protein complexes is observed as shown in Table 2 **—**notably the *Nucleosome H3.1* (CPX‐2556, CPX‐2564), *H3.2* (CPX‐5668) variant, *CENP‐A nucleosome* (CPX‐5647) and *ESCRT* (CPX‐329) complexes. None of them were detected in either of the mutant Tau‐F networks. Complex Portal functional annotation shows that these complexes are integral to the compaction of DNA, a process that plays a pivotal role in regulating DNA accessibility to cellular machinery.

**TABLE 2:** Complexes which are only detected in the Tau‐F network.

Complex AC	Complex name	Biological process
CPX‐329	ESCRT‐III complex	Multivesicular body sorting pathway, autophagosome maturation, nuclear envelope reassembly, viral budding via host ESCRT complex
CPX‐696	PRMT5 methylosome complex, CLNS1A variant	Positive regulation of mRNA splicing, via spliceosome
CPX‐8104	VCP‐NPL4‐UFD1‐FAF2 AAA ATPase complex	Endoplasmic‐reticulum‐associated protein degradation (ERAD) pathway
CPX‐2556	Nucleosome, variant H3.1‐H2A.2‐H2B.1	Chromatin organisation
CPX‐2564	Nucleosome, variant H3.1t‐H2A.2‐H2B.1	Chromatin organisation
CPX‐5668	Nucleosome, variant H3.2‐H2A.2‐H2B.1	Chromatin organisation
CPX‐5647	CENP‐A nucleosome complex	Protein localisation to CENP‐A containing chromatin

Gil et al. [23] demonstrated that AD can be understood as a phenomenon of chromatin dysregulation that directly involves alterations in the epigenetic histone code. Their findings suggest that there is a progressive destabilisation of the tau–chromatin interaction in AD, which leads to subsequent dysregulation of gene expression. This destabilisation may contribute to the complex pathological processes underlying the disease by affecting the regulation of genes involved in neuronal function and maintenance.

The CENP‐A nucleosome complex replaces conventional H3 in the nucleosome core of centromeric chromatin at the inner plate of the kinetochore. It is required for recruitment and assembly of kinetochore proteins, and as a consequence required for progress through mitosis, chromosome segregation and cytokinesis. The *PRMT5 methylosome complex, CLNS1A variant* (CPX‐696) is also detected, which plays a pivotal role in the dimethylation of substrate proteins. CLNS1A complements its function by recruiting the complex to subunits of the spliceosome, thereby regulating cellular splicing activity and influencing the formation of RNA processing bodies. Suganuma et al. [24] have observed that the association of MPTAC with the CLNS1A variant of PRMT5 methylosome complex and snRNP splicing factors facilitates the sensing of metabolic states through their methylation. This intricate regulatory mechanism extends its influence to the splicing fidelity of APP pre‐mRNA. Disruptions in these processes can directly impact the processing of APP, leading to abnormalities in APP fragmentation

Table 2 summarises the complexes enriched exclusively in the Tau‐F network compared to the mutant Tau‐F networks. These include the nucleosome complexes involved in chromatin organisation, the VCP‐ATPase complex engaged in the ER‐associated degradation (ERAD) pathway, the PRMT5 methylosome complex playing a role in the regulation of mRNA splicing and the ESCRT complex, which is involved in multiple processes such as autophagosome maturation and nuclear envelope reassembly.

The Endosomal Sorting Complex Required for Transport *(ESCRT) complex* (CPX‐329) is crucial for autophagosome–lysosome fusion and subsequent degradation. Dysfunction in the ESCRT complex [25], as evidenced in the mutant interactome, could potentially contribute to the accumulation of dysfunctional cellular components. This accumulation might lead to disrupted autophagy and the consequent neurodegeneration observed in AD. The Reactome pathway R‐HSA‐162588 shows that the ESCRT complex has a role in the budding and maturation of HIV virions pathway raises intriguing questions about the potential viral hypothesis in AD. This association prompts a reconsideration of the idea that AD might have viral origins.

Research efforts, such as the study by Esiri and colleagues [26], investigated the prevalence of amyloid plaques in individuals with acquired immunodeficiency syndrome (AIDS). The study compared the frontal and temporal lobes of those who died from AIDS with age‐matched non‐HIV infected controls. The findings supported the theory that a stimulus triggering an inflammatory response in the brain could contribute to the formation of argyrophilic plaques. Further supporting the viral hypothesis, studies by Sathler et al. [27] demonstrated that viral proteins, such as HIV gp120 and FIV gp95, can significantly impact cellular tau pathology. These proteins were found to increase intracellular hyperphosphorylated tau and extracellular tau release. Vijayan et al. [28] provided the first in vivo evidence of a cognitive interaction between an HIV viral protein and Tau mice—in their work they show the HIV gp120 altered cognitive function in Tau mice, with evidence suggesting a cognitive decline in transgenic Tau (P301L) mice compared to the control (HIV gp120 and WT).

Collectively, these findings from various studies lend support to the hypothesis that viral infection may be linked to sporadic forms of AD, offering a new avenue for exploration in the quest to unravel the mysteries of AD.

Both variants of the VCP AAA ATPase complex, *VCP‐NPL4‐UFD1‐FAF2 AAA ATPase* complex (CPX‐8104) and *VCP‐NPL4‐UFD1‐UBXN1 AAA ATPase* complex (CPX‐8105), are enriched in the Tau‐F networks. These complexes are essential for the dislocation and extraction of substrate proteins from cellular structures, facilitating their transport into the cytosol, where they are primarily degraded by the proteasome.

The P301L mutant Tau‐F network failed to express both of these complexes, suggesting a disruption in the proteostasis network. In contrast, the V337M mutant network retained the VCP‐NPL4‐UFD1‐UBXN1 AAA ATPase complex (CPX‐8105), indicating a comparatively more stable protein quality control system in the V337M variant compared to the P301L variant.

The *VCP‐NPL4‐UFD1‐FAF2 AAA ATPase* complex specifically targets GTPase‐activating protein‐binding proteins, GBP1 and GBP2 (Q13283 and Q9UN86), facilitating their extraction from cellular structures and promoting the efficient clearance of heat‐induced stress granules. The *VCP‐NPL4‐UFD1‐UBXN1 AAA ATPase*‐variant of the complex is involved in targeting proteins ubiquitylated on Lys‐6 and Lys‐48 for degradation. This variant also plays a crucial role in the formation of aggresomes—membrane‐less, juxta‐nuclear structures encased in vimentin intermediate filament cages—which sequester misfolded, aggregation‐prone proteins, thereby alleviating proteotoxic cellular stress.

According to Mengus et al. [29], the removal of ubiquitinated substrates, such as the mitofusin MFN2, from the outer mitochondrial membrane by VCP is crucial for the accumulation of PRKN (Parkin) on mitochondria, which in turn drives mitophagy. In cells lacking UBXN1, the induction of mitophagy resulted in impaired mitochondrial translocation of both VCP and PRKN, leading to a reduction in mitophagic flux. As stated by Xie et al. [30], the removal of damaged mitochondria via mitophagy—a targeted macroautophagic process—is crucial for preserving mitochondrial integrity and preventing neurodegenerative and age‐related disorders such as AD.

##### 4.1.1.2 Complexes Not Detected in P301L Mutant But Enriched in Tau‐F Networks

**FIGURE 2:**
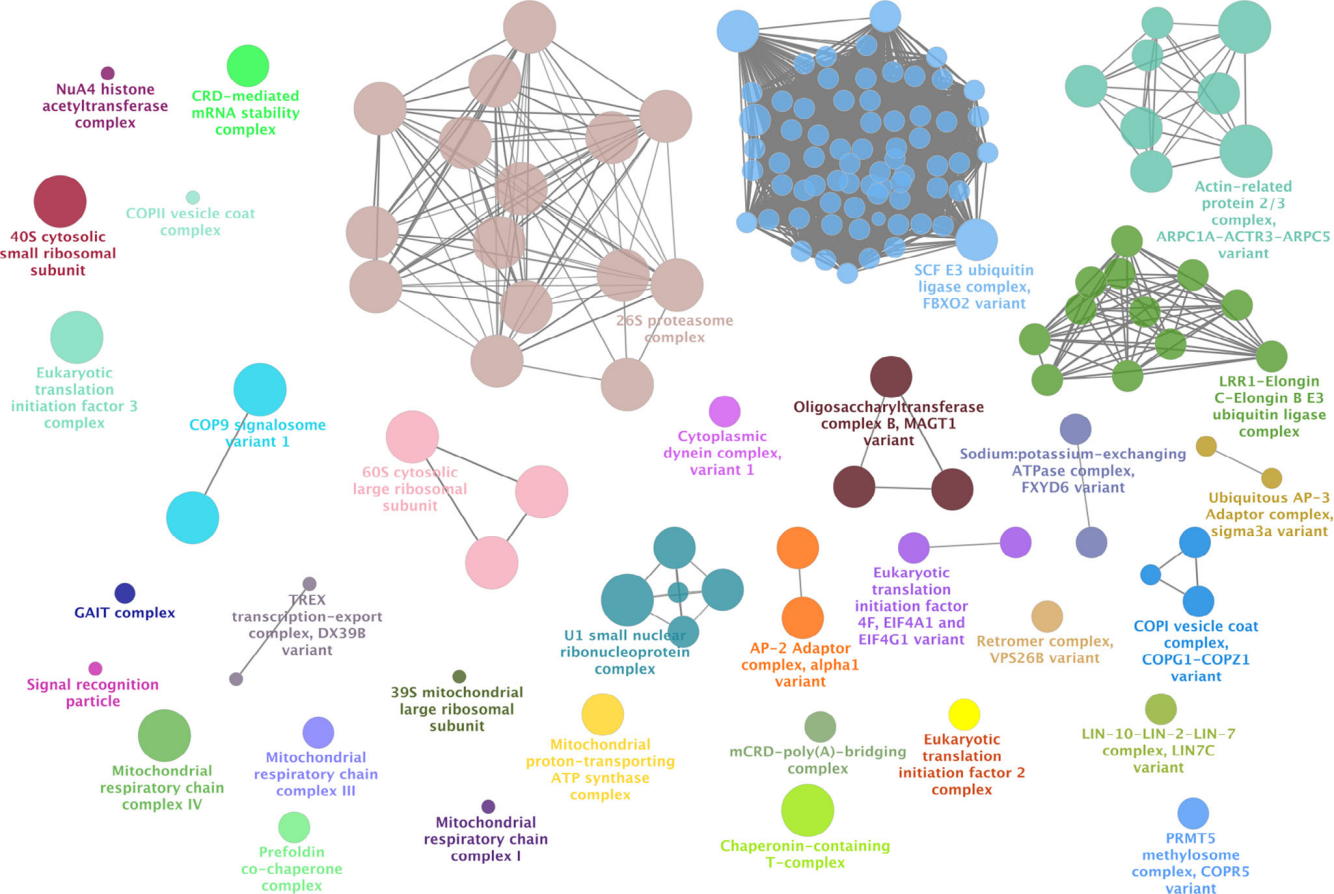
Enrichment analysis of high‐confident human proteins of P301L mutant Tau‐F for complex portal complexes.

Enrichment analysis for the P301L mutant Tau network was conducted similarly to the analysis performed for the Tau‐F network proteins shown in Figure 2. The results provide insights into the altered molecular landscape associated with the mutation in the context of AD.

The complete list of complexes generated from the P301L mutant Tau network enrichment analysis is presented in Table 3. These complexes reflect specific molecular assemblies and pathways that may be selectively enriched or disrupted due to the presence of the P301L mutation. The P301L mutant network does not contain the ESCRT‐III complex, nucleosome complexes, PRMT5 methylosome complex, CLNS1A variant and VCP‐NPL4‐UFD1‐FAF2 AAA ATPase complex, whose functional impact has been discussed in detail above.

**TABLE 3:** Complex Portal complexes enriched for high‐confidence interacting proteins of mutant P301L Tau‐F.

ID	Term	Ontology source	Term *p* value	Term *p* value corrected with Bonferroni step down	Group *p* value	Group *p* value corrected with Bonferroni step down	GOLevels	GOGroups	% Associated genes	Nr. genes	Associated genes found
CPX:1076	mCRD‐poly(A)‐bridging complex	COMPLEXPORTAL_Complexes_07.06.2024	0.00	0.02	0.00	0.01	[−1]	Group00	80.00	4.00	[CSDE1, HNRNPD, PABPC1, SYNCRIP]
CPX:1080	CRD‐mediated mRNA stability complex	COMPLEXPORTAL_Complexes_07.06.2024	0.00	0.00	0.00	0.00	[−1]	Group01	100.00	5.00	[DHX9, HNRNPU, IGF2BP1, SYNCRIP, YBX1]
CPX:2360	COPII vesicle coat complex	COMPLEXPORTAL_Complexes_07.06.2024	0.09	0.28	0.09	0.37	[−1]	Group02	33.33	3.00	[SEC13, SEC24C, SEC31A]
CPX:2476	GAIT complex	COMPLEXPORTAL_Complexes_07.06.2024	0.01	0.06	0.01	0.06	[−1]	Group03	75.00	3.00	[GAPDH, RPL13A, SYNCRIP]
CPX:2652	Signal recognition particle	COMPLEXPORTAL_Complexes_07.06.2024	0.05	0.23	0.05	0.32	[−1]	Group04	42.86	3.00	[SRP14, SRP72, SRP9]
CPX:2716	Eukaryotic translation initiation Factor 2 complex	COMPLEXPORTAL_Complexes_07.06.2024	0.00	0.03	0.00	0.02	[−1]	Group05	100.00	3.00	[EIF2S1, EIF2S2, EIF2S3]
CPX:5025	Cytoplasmic dynein complex, Variant 1	COMPLEXPORTAL_Complexes_07.06.2024	0.00	0.03	0.00	0.03	[−1]	Group06	66.67	4.00	[DYNC1H1, DYNC1I2, DYNLL1, DYNLRB1]
CPX:5223	40S cytosolic small ribosomal subunit	COMPLEXPORTAL_Complexes_07.06.2024	0.00	0.00	0.00	0.00	[−1]	Group07	100.00	30.00	[RACK1, RPS10, RPS11, RPS12, RPS13, RPS14, RPS15, RPS15A, RPS16, RPS17, RPS18, RPS19, RPS2, RPS20, RPS21, RPS23, RPS24, RPS25, RPS26, RPS27A, RPS28, RPS29, RPS3, RPS3A, RPS5, RPS6, RPS7, RPS8, RPS9, RPSA]
CPX:5226	39S mitochondrial large ribosomal subunit	COMPLEXPORTAL_Complexes_07.06.2024	1.00	1.00	1.00	1.00	[−1]	Group08	11.32	6.00	[MRPL16, MRPL33, MRPL34, MRPL46, MRPL48, MRPL9]
CPX:560	Mitochondrial respiratory chain Complex III	COMPLEXPORTAL_Complexes_07.06.2024	0.00	0.01	0.00	0.01	[−1]	Group09	54.55	6.00	[CYC1, UQCR10, UQCRB, UQCRC1, UQCRC2, UQCRH]
CPX:577	Mitochondrial respiratory chain Complex I	COMPLEXPORTAL_Complexes_07.06.2024	0.02	0.14	0.02	0.16	[−1]	Group10	25.00	11.00	[NDUFA13, NDUFA5, NDUFA9, NDUFAB1, NDUFB10, NDUFB3, NDUFB9, NDUFS1, NDUFS3, NDUFS4, NDUFS5]
CPX:6030	Chaperonin‐containing T‐complex	COMPLEXPORTAL_Complexes_07.06.2024	0.00	0.00	0.00	0.00	[−1]	Group11	100.00	8.00	[CCT2, CCT3, CCT4, CCT5, CCT6A, CCT7, CCT8, TCP1]
CPX:6036	Eukaryotic translation initiation Factor 3 complex	COMPLEXPORTAL_Complexes_07.06.2024	0.00	0.00	0.00	0.00	[−1]	Group12	100.00	13.00	[EIF3A, EIF3B, EIF3C, EIF3D, EIF3E, EIF3F, EIF3G, EIF3H, EIF3I, EIF3J, EIF3K, EIF3L, EIF3M]
CPX:6123	Mitochondrial respiratory chain Complex IV	COMPLEXPORTAL_Complexes_07.06.2024	0.00	0.00	0.00	0.00	[−1]	Group13	64.29	9.00	[COX2, COX4I1, COX5A, COX5B, COX6B1, COX6C, COX7A2, COX7C, NDUFA4]
CPX:6149	Prefoldin co‐chaperone complex	COMPLEXPORTAL_Complexes_07.06.2024	0.00	0.03	0.00	0.03	[−1]	Group14	66.67	4.00	[PFDN1, PFDN2, PFDN5, VBP1]
CPX:6151	Mitochondrial proton‐transporting ATP synthase complex	COMPLEXPORTAL_Complexes_07.06.2024	0.00	0.00	0.00	0.00	[−1]	Group15	50.00	10.00	[ATP5F1A, ATP5F1B, ATP5F1C, ATP5F1D, ATP5ME, ATP5MG, ATP5PB, ATP5PD, ATP5PF, ATP5PO]
CPX:7745	LIN‐10‐LIN‐2‐LIN‐7 complex, LIN7C variant	COMPLEXPORTAL_Complexes_07.06.2024	0.00	0.03	0.00	0.02	[−1]	Group16	100.00	3.00	[APBA1, CASK, LIN7C]
CPX:7843	Retromer complex, VPS26B variant	COMPLEXPORTAL_Complexes_07.06.2024	0.00	0.03	0.00	0.02	[−1]	Group17	100.00	3.00	[VPS26B, VPS29, VPS35]
CPX:8149	PRMT5 methylosome complex, COPR5 variant	COMPLEXPORTAL_Complexes_07.06.2024	0.00	0.03	0.00	0.02	[−1]	Group18	100.00	3.00	[COPRS, PRMT5, WDR77]
CPX:978	NuA4 histone acetyltransferase complex	COMPLEXPORTAL_Complexes_07.06.2024	0.08	0.32	0.08	0.39	[−1]	Group19	26.32	5.00	[ACTB, MORF4L1, MORF4L2, RUVBL1, RUVBL2]
CPX:1870	COP9 signalosome Variant 1	COMPLEXPORTAL_Complexes_07.06.2024	0.00	0.00	0.00	0.00	[−1]	Group20	100.00	8.00	[COPS2, COPS3, COPS4, COPS5, COPS6, COPS7A, COPS8, GPS1]
CPX:1871	COP9 signalosome Variant 2	COMPLEXPORTAL_Complexes_07.06.2024	0.00	0.00	0.00	0.00	[−1]	Group20	87.50	7.00	[COPS2, COPS3, COPS4, COPS5, COPS6, COPS8, GPS1]
CPX:5051	Ubiquitous AP‐3 adaptor complex, sigma3a variant	COMPLEXPORTAL_Complexes_07.06.2024	0.01	0.06	0.00	0.03	[−1]	Group21	75.00	3.00	[AP3D1, AP3M1, AP3S1]
CPX:5055	Neuronal AP‐3 adaptor complex, sigma3a variant	COMPLEXPORTAL_Complexes_07.06.2024	0.01	0.06	0.00	0.03	[−1]	Group21	75.00	3.00	[AP3B2, AP3D1, AP3S1]
CPX:5149	AP‐2 adaptor complex, alpha1 variant	COMPLEXPORTAL_Complexes_07.06.2024	0.00	0.00	0.00	0.00	[−1]	Group22	100.00	4.00	[AP2A1, AP2B1, AP2M1, AP2S1]
CPX:5150	AP‐2 adaptor complex, alpha2 variant	COMPLEXPORTAL_Complexes_07.06.2024	0.00	0.00	0.00	0.00	[−1]	Group22	100.00	4.00	[AP2A2, AP2B1, AP2M1, AP2S1]
CPX:2666	Eukaryotic translation initiation Factor 4F, EIF4A1 and EIF4G1 variant	COMPLEXPORTAL_Complexes_07.06.2024	0.00	0.03	0.00	0.00	[−1]	Group23	100.00	3.00	[EIF4A1, EIF4E, EIF4G1]
CPX:5634	Eukaryotic translation initiation Factor 4F, EIF4A2 and EIF4G1 variant	COMPLEXPORTAL_Complexes_07.06.2024	0.00	0.03	0.00	0.00	[−1]	Group23	100.00	3.00	[EIF4A2, EIF4E, EIF4G1]
CPX:2488	TREX transcription‐export complex, DX39B variant	COMPLEXPORTAL_Complexes_07.06.2024	0.04	0.24	0.05	0.31	[−1]	Group24	31.25	5.00	[ALYREF, CHTOP, DDX39B, ERH, SARNP]
CPX:7261	TREX transcription‐export complex, DX39A variant	COMPLEXPORTAL_Complexes_07.06.2024	0.13	0.26	0.05	0.31	[−1]	Group24	25.00	4.00	[ALYREF, CHTOP, ERH, SARNP]
CPX:8144	Sodium:potassium‐exchanging ATPase complex, FXYD6 variant	COMPLEXPORTAL_Complexes_07.06.2024	0.00	0.03	0.00	0.00	[−1]	Group25	100.00	3.00	[ATP1A1, ATP1B1, FXYD6]
CPX:8146	Sodium:potassium‐exchanging ATPase complex, FXYD7 variant	COMPLEXPORTAL_Complexes_07.06.2024	0.00	0.03	0.00	0.00	[−1]	Group25	100.00	3.00	[ATP1A1, ATP1B1, FXYD7]
CPX:7803	COPI vesicle coat complex, COPG1‐COPZ1 variant	COMPLEXPORTAL_Complexes_07.06.2024	0.00	0.01	0.00	0.03	[−1]	Group26	71.43	5.00	[ARCN1, COPA, COPB1, COPB2, COPG1]
CPX:7969	COPI vesicle coat complex, COPG2‐COPZ1 variant	COMPLEXPORTAL_Complexes_07.06.2024	0.01	0.06	0.00	0.03	[−1]	Group26	57.14	4.00	[ARCN1, COPA, COPB1, COPB2]
CPX:7970	COPI vesicle coat complex, COPG1‐COPZ2 variant	COMPLEXPORTAL_Complexes_07.06.2024	0.00	0.01	0.00	0.03	[−1]	Group26	71.43	5.00	[ARCN1, COPA, COPB1, COPB2, COPG1]
CPX:5183	60S cytosolic large ribosomal subunit	COMPLEXPORTAL_Complexes_07.06.2024	0.00	0.00	0.00	0.00	[−1]	Group27	80.43	37.00	[RPL10A, RPL11, RPL12, RPL13, RPL13A, RPL14, RPL15, RPL17, RPL18, RPL18A, RPL21, RPL23, RPL23A, RPL24, RPL27, RPL27A, RPL28, RPL29, RPL30, RPL31, RPL32, RPL34, RPL35, RPL35A, RPL36, RPL37A, RPL38, RPL4, RPL5, RPL7, RPL7A, RPL8, RPL9, RPLP0, RPLP1, RPLP2, UBA52]
CPX:7664	60S cytosolic large ribosomal subunit, testis‐specific variant	COMPLEXPORTAL_Complexes_07.06.2024	0.00	0.00	0.00	0.00	[−1]	Group27	80.43	37.00	[RPL10A, RPL11, RPL12, RPL13, RPL13A, RPL14, RPL15, RPL17, RPL18, RPL18A, RPL21, RPL23, RPL23A, RPL24, RPL27, RPL27A, RPL28, RPL29, RPL30, RPL31, RPL32, RPL34, RPL35, RPL35A, RPL36, RPL37A, RPL38, RPL4, RPL5, RPL7, RPL7A, RPL8, RPL9, RPLP0, RPLP1, RPLP2, UBA52]
CPX:7665	60S cytosolic large ribosomal subunit, striated muscle variant	COMPLEXPORTAL_Complexes_07.06.2024	0.00	0.00	0.00	0.00	[−1]	Group27	78.72	37.00	[RPL10A, RPL11, RPL12, RPL13, RPL13A, RPL14, RPL15, RPL17, RPL18, RPL18A, RPL21, RPL23, RPL23A, RPL24, RPL27, RPL27A, RPL28, RPL29, RPL30, RPL31, RPL32, RPL34, RPL35, RPL35A, RPL36, RPL37A, RPL38, RPL4, RPL5, RPL7, RPL7A, RPL8, RPL9, RPLP0, RPLP1, RPLP2, UBA52]
CPX:5621	Oligosaccharyltransferase Complex A	COMPLEXPORTAL_Complexes_07.06.2024	0.00	0.00	0.00	0.00	[−1]	Group28	66.67	6.00	[DAD1, DDOST, RPN1, RPN2, STT3A, TMEM258]
CPX:5622	Oligosaccharyltransferase Complex B, MAGT1 variant	COMPLEXPORTAL_Complexes_07.06.2024	0.00	0.00	0.00	0.00	[−1]	Group28	75.00	6.00	[DAD1, DDOST, RPN1, RPN2, STT3B, TMEM258]
CPX:8738	Oligosaccharyltransferase Complex B, TUCS3 variant	COMPLEXPORTAL_Complexes_07.06.2024	0.00	0.00	0.00	0.00	[−1]	Group28	75.00	6.00	[DAD1, DDOST, RPN1, RPN2, STT3B, TMEM258]
CPX:2391	U4/U6.U5 small nuclear ribonucleoprotein complex	COMPLEXPORTAL_Complexes_07.06.2024	0.00	0.00	0.00	0.00	[−1]	Group29	37.50	12.00	[LSM3, LSM4, PRPF31, SART1, SNRPA, SNRPD1, SNRPD2, SNRPD3, SNRPE, SNRPF, SNRPG, SNU13]
CPX:2392	U1 small nuclear ribonucleoprotein complex	COMPLEXPORTAL_Complexes_07.06.2024	0.00	0.00	0.00	0.00	[−1]	Group29	90.00	9.00	[SNRNP70, SNRPA, SNRPC, SNRPD1, SNRPD2, SNRPD3, SNRPE, SNRPF, SNRPG]
CPX:2539	U2 small nuclear ribonucleoprotein complex	COMPLEXPORTAL_Complexes_07.06.2024	0.00	0.03	0.00	0.00	[−1]	Group29	36.00	9.00	[HTATSF1, SF3A2, SF3B6, SNRPD1, SNRPD2, SNRPD3, SNRPE, SNRPF, SNRPG]
CPX:2705	U7 small nuclear ribonucleoprotein complex	COMPLEXPORTAL_Complexes_07.06.2024	0.01	0.06	0.00	0.00	[−1]	Group29	57.14	4.00	[SNRPD3, SNRPE, SNRPF, SNRPG]
CPX:6033	Sm complex	COMPLEXPORTAL_Complexes_07.06.2024	0.00	0.00	0.00	0.00	[−1]	Group29	85.71	6.00	[SNRPD1, SNRPD2, SNRPD3, SNRPE, SNRPF, SNRPG]
CPX:2490	Actin‐related protein 2/3 complex, ARPC1A‐ACTR3B‐ARPC5 variant	COMPLEXPORTAL_Complexes_07.06.2024	0.00	0.00	0.00	0.00	[−1]	Group30	85.71	6.00	[ACTR2, ARPC1A, ARPC2, ARPC3, ARPC4, ARPC5]
CPX:2579	Actin‐related protein 2/3 complex, ARPC1B‐ACTR3‐ARPC5 variant	COMPLEXPORTAL_Complexes_07.06.2024	0.00	0.00	0.00	0.00	[−1]	Group30	85.71	6.00	[ACTR2, ACTR3, ARPC2, ARPC3, ARPC4, ARPC5]
CPX:2580	Actin‐related protein 2/3 complex, ARPC1B‐ACTR3B‐ARPC5L variant	COMPLEXPORTAL_Complexes_07.06.2024	0.00	0.01	0.00	0.00	[−1]	Group30	71.43	5.00	[ACTR2, ARPC2, ARPC3, ARPC4, ARPC5L]
CPX:2583	Actin‐related protein 2/3 complex, ARPC1B‐ACTR3B‐ARPC5 variant	COMPLEXPORTAL_Complexes_07.06.2024	0.00	0.01	0.00	0.00	[−1]	Group30	71.43	5.00	[ACTR2, ARPC2, ARPC3, ARPC4, ARPC5]
CPX:2586	Actin‐related protein 2/3 complex, ARPC1A‐ACTR3‐ARPC5 variant	COMPLEXPORTAL_Complexes_07.06.2024	0.00	0.00	0.00	0.00	[−1]	Group30	100.00	7.00	[ACTR2, ACTR3, ARPC1A, ARPC2, ARPC3, ARPC4, ARPC5]
CPX:2592	Actin‐related protein 2/3 complex, ARPC1A‐ACTR3‐ARPC5L variant	COMPLEXPORTAL_Complexes_07.06.2024	0.00	0.00	0.00	0.00	[−1]	Group30	100.00	7.00	[ACTR2, ACTR3, ARPC1A, ARPC2, ARPC3, ARPC4, ARPC5L]
CPX:2663	Actin‐related protein 2/3 complex, ARPC1B‐ACTR3‐ARPC5L variant	COMPLEXPORTAL_Complexes_07.06.2024	0.00	0.00	0.00	0.00	[−1]	Group30	85.71	6.00	[ACTR2, ACTR3, ARPC2, ARPC3, ARPC4, ARPC5L]
CPX:2668	Actin‐related protein 2/3 complex, ARPC1B‐ACTR3B‐ARPC5L variant	COMPLEXPORTAL_Complexes_07.06.2024	0.00	0.00	0.00	0.00	[−1]	Group30	85.71	6.00	[ACTR2, ARPC1A, ARPC2, ARPC3, ARPC4, ARPC5L]
CPX:2214	LRR1‐Elongin C‐Elongin B E3 ubiquitin ligase complex	COMPLEXPORTAL_Complexes_07.06.2024	0.00	0.02	0.13	0.39	[−1]	Group31	80.00	4.00	[CUL2, ELOB, ELOC, RBX1]
CPX:2217	FEM1A‐Elongin C‐Elongin B E3 ubiquitin ligase complex	COMPLEXPORTAL_Complexes_07.06.2024	0.00	0.02	0.13	0.39	[−1]	Group31	80.00	4.00	[CUL2, ELOB, ELOC, RBX1]
CPX:2218	FEB1B‐Elongin C‐Elongin B E3 ubiquitin ligase complex	COMPLEXPORTAL_Complexes_07.06.2024	0.00	0.02	0.13	0.39	[−1]	Group31	80.00	4.00	[CUL2, ELOB, ELOC, RBX1]
CPX:2219	FEB1C‐Elongin C‐Elongin B E3 ubiquitin ligase complex	COMPLEXPORTAL_Complexes_07.06.2024	0.00	0.02	0.13	0.39	[−1]	Group31	80.00	4.00	[CUL2, ELOB, ELOC, RBX1]
CPX:2220	ZYG11B‐Elongin C‐Elongin B E3 ubiquitin ligase complex	COMPLEXPORTAL_Complexes_07.06.2024	0.00	0.02	0.13	0.39	[−1]	Group31	80.00	4.00	[CUL2, ELOB, ELOC, RBX1]
CPX:2221	APPBP2‐Elongin C‐Elongin B E3 ubiquitin ligase complex	COMPLEXPORTAL_Complexes_07.06.2024	0.00	0.02	0.13	0.39	[−1]	Group31	80.00	4.00	[CUL2, ELOB, ELOC, RBX1]
CPX:2222	ZER1‐Elongin C‐Elongin B E3 ubiquitin ligase complex	COMPLEXPORTAL_Complexes_07.06.2024	0.00	0.02	0.13	0.39	[−1]	Group31	80.00	4.00	[CUL2, ELOB, ELOC, RBX1]
CPX:2223	KLHDC10‐Elongin C‐Elongin B E3 ubiquitin ligase complex	COMPLEXPORTAL_Complexes_07.06.2024	0.00	0.02	0.13	0.39	[−1]	Group31	80.00	4.00	[CUL2, ELOB, ELOC, RBX1]
CPX:2226	KLHDC2‐Elongin C‐Elongin B E3 ubiquitin ligase complex	COMPLEXPORTAL_Complexes_07.06.2024	0.00	0.02	0.13	0.39	[−1]	Group31	80.00	4.00	[CUL2, ELOB, ELOC, RBX1]
CPX:2228	KLHDC3‐Elongin C‐Elongin B E3 ubiquitin ligase complex	COMPLEXPORTAL_Complexes_07.06.2024	0.00	0.02	0.13	0.39	[−1]	Group31	80.00	4.00	[CUL2, ELOB, ELOC, RBX1]
CPX:2229	PRAME‐Elongin C‐Elongin B E3 ubiquitin ligase complex	COMPLEXPORTAL_Complexes_07.06.2024	0.00	0.02	0.13	0.39	[−1]	Group31	80.00	4.00	[CUL2, ELOB, ELOC, RBX1]
CPX:2250	VHL‐Elongin C‐Elongin B E3 ubiquitin ligase complex	COMPLEXPORTAL_Complexes_07.06.2024	0.00	0.02	0.13	0.39	[−1]	Group31	80.00	4.00	[CUL2, ELOB, ELOC, RBX1]
CPX:5993	26S proteasome complex	COMPLEXPORTAL_Complexes_07.06.2024	0.00	0.00	0.00	0.00	[−1]	Group32	96.97	32.00	[ADRM1, PSMA1, PSMA2, PSMA3, PSMA4, PSMA5, PSMA6, PSMA7, PSMB1, PSMB2, PSMB3, PSMB4, PSMB5, PSMB6, PSMB7, PSMC1, PSMC2, PSMC3, PSMC4, PSMC5, PSMC6, PSMD1, PSMD11, PSMD12, PSMD13, PSMD14, PSMD2, PSMD3, PSMD4, PSMD6, PSMD7, PSMD8]
CPX:8806	20S proteasome complex	COMPLEXPORTAL_Complexes_07.06.2024	0.00	0.00	0.00	0.00	[−1]	Group32	100.00	14.00	[PSMA1, PSMA2, PSMA3, PSMA4, PSMA5, PSMA6, PSMA7, PSMB1, PSMB2, PSMB3, PSMB4, PSMB5, PSMB6, PSMB7]
CPX:8841	PA200‐20S single‐capped proteasome	COMPLEXPORTAL_Complexes_07.06.2024	0.00	0.00	0.00	0.00	[−1]	Group32	93.33	14.00	[PSMA1, PSMA2, PSMA3, PSMA4, PSMA5, PSMA6, PSMA7, PSMB1, PSMB2, PSMB3, PSMB4, PSMB5, PSMB6, PSMB7]
CPX:8842	PA28‐alphabeta double‐capped 20S proteasome complex	COMPLEXPORTAL_Complexes_07.06.2024	0.00	0.00	0.00	0.00	[−1]	Group32	93.75	15.00	[PSMA1, PSMA2, PSMA3, PSMA4, PSMA5, PSMA6, PSMA7, PSMB1, PSMB2, PSMB3, PSMB4, PSMB5, PSMB6, PSMB7, PSME1]
CPX:8964	19S proteasome regulatory complex	COMPLEXPORTAL_Complexes_07.06.2024	0.00	0.00	0.00	0.00	[−1]	Group32	94.74	18.00	[ADRM1, PSMC1, PSMC2, PSMC3, PSMC4, PSMC5, PSMC6, PSMD1, PSMD11, PSMD12, PSMD13, PSMD14, PSMD2, PSMD3, PSMD4, PSMD6, PSMD7, PSMD8]
CPX:9001	PA28‐gamma single‐capped 20S proteasome complex	COMPLEXPORTAL_Complexes_07.06.2024	0.00	0.00	0.00	0.00	[−1]	Group32	93.33	14.00	[PSMA1, PSMA2, PSMA3, PSMA4, PSMA5, PSMA6, PSMA7, PSMB1, PSMB2, PSMB3, PSMB4, PSMB5, PSMB6, PSMB7]
CPX:9002	PA28‐alphabeta single‐capped 20S proteasome complex	COMPLEXPORTAL_Complexes_07.06.2024	0.00	0.00	0.00	0.00	[−1]	Group32	93.75	15.00	[PSMA1, PSMA2, PSMA3, PSMA4, PSMA5, PSMA6, PSMA7, PSMB1, PSMB2, PSMB3, PSMB4, PSMB5, PSMB6, PSMB7, PSME1]
CPX:9003	20S immunoproteasome complex	COMPLEXPORTAL_Complexes_07.06.2024	0.00	0.00	0.00	0.00	[−1]	Group32	78.57	11.00	[PSMA1, PSMA2, PSMA3, PSMA4, PSMA5, PSMA6, PSMA7, PSMB1, PSMB2, PSMB3, PSMB4]
CPX:9004	20S thymoproteasome complex	COMPLEXPORTAL_Complexes_07.06.2024	0.00	0.00	0.00	0.00	[−1]	Group32	78.57	11.00	[PSMA1, PSMA2, PSMA3, PSMA4, PSMA5, PSMA6, PSMA7, PSMB1, PSMB2, PSMB3, PSMB4]
CPX:9021	20S spermatoproteasome complex	COMPLEXPORTAL_Complexes_07.06.2024	0.00	0.00	0.00	0.00	[−1]	Group32	92.86	13.00	[PSMA1, PSMA2, PSMA3, PSMA4, PSMA5, PSMA6, PSMB1, PSMB2, PSMB3, PSMB4, PSMB5, PSMB6, PSMB7]
CPX:9022	PA28‐gamma double‐capped 20S proteasome complex	COMPLEXPORTAL_Complexes_07.06.2024	0.00	0.00	0.00	0.00	[−1]	Group32	93.33	14.00	[PSMA1, PSMA2, PSMA3, PSMA4, PSMA5, PSMA6, PSMA7, PSMB1, PSMB2, PSMB3, PSMB4, PSMB5, PSMB6, PSMB7]
CPX:9063	PA200‐20S‐PA200 double‐capped proteasome complex	COMPLEXPORTAL_Complexes_07.06.2024	0.00	0.00	0.00	0.00	[−1]	Group32	93.33	14.00	[PSMA1, PSMA2, PSMA3, PSMA4, PSMA5, PSMA6, PSMA7, PSMB1, PSMB2, PSMB3, PSMB4, PSMB5, PSMB6, PSMB7]
CPX:9082	19S‐20S‐PA28‐alphabeta hybrid proteasome complex	COMPLEXPORTAL_Complexes_07.06.2024	0.00	0.00	0.00	0.00	[−1]	Group32	94.29	33.00	[ADRM1, PSMA1, PSMA2, PSMA3, PSMA4, PSMA5, PSMA6, PSMA7, PSMB1, PSMB2, PSMB3, PSMB4, PSMB5, PSMB6, PSMB7, PSMC1, PSMC2, PSMC3, PSMC4, PSMC5, PSMC6, PSMD1, PSMD11, PSMD12, PSMD13, PSMD14, PSMD2, PSMD3, PSMD4, PSMD6, PSMD7, PSMD8, PSME1]
CPX:9085	19S‐20S‐PA28‐gamma hybrid proteasome complex	COMPLEXPORTAL_Complexes_07.06.2024	0.00	0.00	0.00	0.00	[−1]	Group32	94.12	32.00	[ADRM1, PSMA1, PSMA2, PSMA3, PSMA4, PSMA5, PSMA6, PSMA7, PSMB1, PSMB2, PSMB3, PSMB4, PSMB5, PSMB6, PSMB7, PSMC1, PSMC2, PSMC3, PSMC4, PSMC5, PSMC6, PSMD1, PSMD11, PSMD12, PSMD13, PSMD14, PSMD2, PSMD3, PSMD4, PSMD6, PSMD7, PSMD8]
CPX:9086	30S proteasome complex	COMPLEXPORTAL_Complexes_07.06.2024	0.00	0.00	0.00	0.00	[−1]	Group32	96.97	32.00	[ADRM1, PSMA1, PSMA2, PSMA3, PSMA4, PSMA5, PSMA6, PSMA7, PSMB1, PSMB2, PSMB3, PSMB4, PSMB5, PSMB6, PSMB7, PSMC1, PSMC2, PSMC3, PSMC4, PSMC5, PSMC6, PSMD1, PSMD11, PSMD12, PSMD13, PSMD14, PSMD2, PSMD3, PSMD4, PSMD6, PSMD7, PSMD8]
CPX:2239	SCF E3 ubiquitin ligase complex, FBXL5 variant	COMPLEXPORTAL_Complexes_07.06.2024	0.01	0.06	0.20	0.41	[−1]	Group33	75.00	3.00	[CUL1, RBX1, SKP1]
CPX:2241	SCF E3 ubiquitin ligase complex, FBXL13 variant	COMPLEXPORTAL_Complexes_07.06.2024	0.01	0.06	0.20	0.41	[−1]	Group33	75.00	3.00	[CUL1, RBX1, SKP1]
CPX:2319	SCF E3 ubiquitin ligase complex, FBXL14 variant	COMPLEXPORTAL_Complexes_07.06.2024	0.01	0.06	0.20	0.41	[−1]	Group33	75.00	3.00	[CUL1, RBX1, SKP1]
CPX:2343	SCF E3 ubiquitin ligase complex, FBXL16 variant	COMPLEXPORTAL_Complexes_07.06.2024	0.01	0.06	0.20	0.41	[−1]	Group33	75.00	3.00	[CUL1, RBX1, SKP1]
CPX:2365	SCF E3 ubiquitin ligase complex, BTRC variant	COMPLEXPORTAL_Complexes_07.06.2024	0.01	0.06	0.20	0.41	[−1]	Group33	75.00	3.00	[CUL1, RBX1, SKP1]
CPX:2438	SCF E3 ubiquitin ligase complex, FBXL8 variant	COMPLEXPORTAL_Complexes_07.06.2024	0.01	0.06	0.20	0.41	[−1]	Group33	75.00	3.00	[CUL1, RBX1, SKP1]
CPX:2489	SCF E3 ubiquitin ligase complex, FBXL22 variant	COMPLEXPORTAL_Complexes_07.06.2024	0.01	0.06	0.20	0.41	[−1]	Group33	75.00	3.00	[CUL1, RBX1, SKP1]
CPX:2492	SCF E3 ubiquitin ligase complex, FBXL15 variant	COMPLEXPORTAL_Complexes_07.06.2024	0.01	0.06	0.20	0.41	[−1]	Group33	75.00	3.00	[CUL1, RBX1, SKP1]
CPX:2512	SCF E3 ubiquitin ligase complex, FBXL4 variant	COMPLEXPORTAL_Complexes_07.06.2024	0.01	0.06	0.20	0.41	[−1]	Group33	75.00	3.00	[CUL1, RBX1, SKP1]
CPX:2516	SCF E3 ubiquitin ligase complex, FBXL6 variant	COMPLEXPORTAL_Complexes_07.06.2024	0.01	0.06	0.20	0.41	[−1]	Group33	75.00	3.00	[CUL1, RBX1, SKP1]
CPX:2538	SCF E3 ubiquitin ligase complex, KDM2A variant	COMPLEXPORTAL_Complexes_07.06.2024	0.01	0.06	0.20	0.41	[−1]	Group33	75.00	3.00	[CUL1, RBX1, SKP1]
CPX:2553	SCF E3 ubiquitin ligase complex, FBXL18 variant	COMPLEXPORTAL_Complexes_07.06.2024	0.00	0.03	0.20	0.41	[−1]	Group33	100.00	3.00	[CUL1, RBX1, SKP1]
CPX:2554	SCF E3 ubiquitin ligase complex, FBXL19 variant	COMPLEXPORTAL_Complexes_07.06.2024	0.01	0.06	0.20	0.41	[−1]	Group33	75.00	3.00	[CUL1, RBX1, SKP1]
CPX:2658	SCF E3 ubiquitin ligase complex, FBXL12 variant	COMPLEXPORTAL_Complexes_07.06.2024	0.01	0.06	0.20	0.41	[−1]	Group33	75.00	3.00	[CUL1, RBX1, SKP1]
CPX:2683	SCF E3 ubiquitin ligase complex, FBXL7 variant	COMPLEXPORTAL_Complexes_07.06.2024	0.01	0.06	0.20	0.41	[−1]	Group33	75.00	3.00	[CUL1, RBX1, SKP1]
CPX:2748	SCF E3 ubiquitin ligase complex, FBXL21 variant	COMPLEXPORTAL_Complexes_07.06.2024	0.00	0.03	0.20	0.41	[−1]	Group33	100.00	3.00	[CUL1, RBX1, SKP1]
CPX:2773	SCF E3 ubiquitin ligase complex, FBXL17 variant	COMPLEXPORTAL_Complexes_07.06.2024	0.01	0.06	0.20	0.41	[−1]	Group33	75.00	3.00	[CUL1, RBX1, SKP1]
CPX:2832	SCF E3 ubiquitin ligase complex, KDM2B variant	COMPLEXPORTAL_Complexes_07.06.2024	0.01	0.06	0.20	0.41	[−1]	Group33	75.00	3.00	[CUL1, RBX1, SKP1]
CPX:2873	SCF E3 ubiquitin ligase complex, FBXL20 variant	COMPLEXPORTAL_Complexes_07.06.2024	0.01	0.06	0.20	0.41	[−1]	Group33	75.00	3.00	[CUL1, RBX1, SKP1]
CPX:3291	SCF E3 ubiquitin ligase complex, FBXL3 variant	COMPLEXPORTAL_Complexes_07.06.2024	0.01	0.06	0.20	0.41	[−1]	Group33	75.00	3.00	[CUL1, RBX1, SKP1]
CPX:3292	SCF E3 ubiquitin ligase complex, FBXL2 variant	COMPLEXPORTAL_Complexes_07.06.2024	0.01	0.06	0.20	0.41	[−1]	Group33	75.00	3.00	[CUL1, RBX1, SKP1]
CPX:3295	SCF E3 ubiquitin ligase complex, SKP2 variant	COMPLEXPORTAL_Complexes_07.06.2024	0.01	0.06	0.20	0.41	[−1]	Group33	75.00	3.00	[CUL1, RBX1, SKP1]
CPX:7747	SCF E3 ubiquitin ligase complex, FBXW2 variant	COMPLEXPORTAL_Complexes_07.06.2024	0.01	0.06	0.20	0.41	[−1]	Group33	75.00	3.00	[CUL1, RBX1, SKP1]
CPX:7761	SCF E3 ubiquitin ligase complex, FBXW4 variant	COMPLEXPORTAL_Complexes_07.06.2024	0.01	0.06	0.20	0.41	[−1]	Group33	75.00	3.00	[CUL1, RBX1, SKP1]
CPX:7762	SCF E3 ubiquitin ligase complex, FBXW5 variant	COMPLEXPORTAL_Complexes_07.06.2024	0.01	0.06	0.20	0.41	[−1]	Group33	75.00	3.00	[CUL1, RBX1, SKP1]
CPX:7763	SCF E3 ubiquitin ligase complex, FBXW7 variant	COMPLEXPORTAL_Complexes_07.06.2024	0.01	0.06	0.20	0.41	[−1]	Group33	75.00	3.00	[CUL1, RBX1, SKP1]
CPX:7784	SCF E3 ubiquitin ligase complex, FBXW8‐CUL7 variant	COMPLEXPORTAL_Complexes_07.06.2024	0.02	0.13	0.20	0.41	[−1]	Group33	60.00	3.00	[CUL1, RBX1, SKP1]
CPX:7785	SCF E3 ubiquitin ligase complex, FBXW9 variant	COMPLEXPORTAL_Complexes_07.06.2024	0.01	0.06	0.20	0.41	[−1]	Group33	75.00	3.00	[CUL1, RBX1, SKP1]
CPX:7786	SCF E3 ubiquitin ligase complex, FBXW10 variant	COMPLEXPORTAL_Complexes_07.06.2024	0.01	0.06	0.20	0.41	[−1]	Group33	75.00	3.00	[CUL1, RBX1, SKP1]
CPX:7821	SCF E3 ubiquitin ligase complex, FBXW11 variant	COMPLEXPORTAL_Complexes_07.06.2024	0.01	0.06	0.20	0.41	[−1]	Group33	75.00	3.00	[CUL1, RBX1, SKP1]
CPX:7822	SCF E3 ubiquitin ligase complex, FBXW12 variant	COMPLEXPORTAL_Complexes_07.06.2024	0.01	0.06	0.20	0.41	[−1]	Group33	75.00	3.00	[CUL1, RBX1, SKP1]
CPX:7846	SCF E3 ubiquitin ligase complex, CCNF variant	COMPLEXPORTAL_Complexes_07.06.2024	0.01	0.06	0.20	0.41	[−1]	Group33	75.00	3.00	[CUL1, RBX1, SKP1]
CPX:7847	SCF E3 ubiquitin ligase complex, FBXO2 variant	COMPLEXPORTAL_Complexes_07.06.2024	0.00	0.00	0.20	0.41	[−1]	Group33	100.00	4.00	[CUL1, FBXO2, RBX1, SKP1]
CPX:7881	SCF E3 ubiquitin ligase complex, FBXO3 variant	COMPLEXPORTAL_Complexes_07.06.2024	0.01	0.06	0.20	0.41	[−1]	Group33	75.00	3.00	[CUL1, RBX1, SKP1]
CPX:7882	SCF E3 ubiquitin ligase complex, FBXO4 variant	COMPLEXPORTAL_Complexes_07.06.2024	0.01	0.06	0.20	0.41	[−1]	Group33	75.00	3.00	[CUL1, RBX1, SKP1]
CPX:7904	SCF E3 ubiquitin ligase complex, FBXO5 variant	COMPLEXPORTAL_Complexes_07.06.2024	0.01	0.06	0.20	0.41	[−1]	Group33	75.00	3.00	[CUL1, RBX1, SKP1]
CPX:7905	SCF E3 ubiquitin ligase complex, FBXO6 variant	COMPLEXPORTAL_Complexes_07.06.2024	0.01	0.06	0.20	0.41	[−1]	Group33	75.00	3.00	[CUL1, RBX1, SKP1]
CPX:7906	SCF E3 ubiquitin ligase complex, FBXO7 variant	COMPLEXPORTAL_Complexes_07.06.2024	0.01	0.06	0.20	0.41	[−1]	Group33	75.00	3.00	[CUL1, RBX1, SKP1]
CPX:7921	SCF E3 ubiquitin ligase complex, FBXO8 variant	COMPLEXPORTAL_Complexes_07.06.2024	0.01	0.06	0.20	0.41	[−1]	Group33	75.00	3.00	[CUL1, RBX1, SKP1]
CPX:7922	SCF E3 ubiquitin ligase complex, FBXO9 variant	COMPLEXPORTAL_Complexes_07.06.2024	0.01	0.06	0.20	0.41	[−1]	Group33	75.00	3.00	[CUL1, RBX1, SKP1]
CPX:7923	SCF E3 ubiquitin ligase complex, FBXO10 variant	COMPLEXPORTAL_Complexes_07.06.2024	0.01	0.06	0.20	0.41	[−1]	Group33	75.00	3.00	[CUL1, RBX1, SKP1]
CPX:7924	SCF E3 ubiquitin ligase complex, FBXO11 variant	COMPLEXPORTAL_Complexes_07.06.2024	0.01	0.06	0.20	0.41	[−1]	Group33	75.00	3.00	[CUL1, RBX1, SKP1]
CPX:7925	SCF E3 ubiquitin ligase complex, FBXO15 variant	COMPLEXPORTAL_Complexes_07.06.2024	0.01	0.06	0.20	0.41	[−1]	Group33	75.00	3.00	[CUL1, RBX1, SKP1]
CPX:7926	SCF E3 ubiquitin ligase complex, FBXO16 variant	COMPLEXPORTAL_Complexes_07.06.2024	0.01	0.06	0.20	0.41	[−1]	Group33	75.00	3.00	[CUL1, RBX1, SKP1]
CPX:7927	SCF E3 ubiquitin ligase complex, FBXO17 variant	COMPLEXPORTAL_Complexes_07.06.2024	0.01	0.06	0.20	0.41	[−1]	Group33	75.00	3.00	[CUL1, RBX1, SKP1]
CPX:7928	SCF E3 ubiquitin ligase complex, FBH1 variant	COMPLEXPORTAL_Complexes_07.06.2024	0.01	0.06	0.20	0.41	[−1]	Group33	75.00	3.00	[CUL1, RBX1, SKP1]
CPX:7929	SCF E3 ubiquitin ligase complex, LMO7 variant	COMPLEXPORTAL_Complexes_07.06.2024	0.01	0.06	0.20	0.41	[−1]	Group33	75.00	3.00	[CUL1, RBX1, SKP1]
CPX:7930	SCF E3 ubiquitin ligase complex, FBXO21 variant	COMPLEXPORTAL_Complexes_07.06.2024	0.00	0.00	0.20	0.41	[−1]	Group33	100.00	4.00	[CUL1, FBXO21, RBX1, SKP1]
CPX:7962	SCF E3 ubiquitin ligase complex, FBXO22 variant	COMPLEXPORTAL_Complexes_07.06.2024	0.01	0.06	0.20	0.41	[−1]	Group33	75.00	3.00	[CUL1, RBX1, SKP1]
CPX:7963	SCF E3 ubiquitin ligase complex, FBXO24 variant	COMPLEXPORTAL_Complexes_07.06.2024	0.01	0.06	0.20	0.41	[−1]	Group33	75.00	3.00	[CUL1, RBX1, SKP1]
CPX:7965	SCF E3 ubiquitin ligase complex, FBXO25 variant	COMPLEXPORTAL_Complexes_07.06.2024	0.01	0.06	0.20	0.41	[−1]	Group33	75.00	3.00	[CUL1, RBX1, SKP1]
CPX:7966	SCF E3 ubiquitin ligase complex, FBXO27 variant	COMPLEXPORTAL_Complexes_07.06.2024	0.01	0.06	0.20	0.41	[−1]	Group33	75.00	3.00	[CUL1, RBX1, SKP1]
CPX:7967	SCF E3 ubiquitin ligase complex, FBXO28 variant	COMPLEXPORTAL_Complexes_07.06.2024	0.01	0.06	0.20	0.41	[−1]	Group33	75.00	3.00	[CUL1, RBX1, SKP1]
CPX:7968	SCF E3 ubiquitin ligase complex, FBXO30 variant	COMPLEXPORTAL_Complexes_07.06.2024	0.01	0.06	0.20	0.41	[−1]	Group33	75.00	3.00	[CUL1, RBX1, SKP1]
CPX:7971	SCF E3 ubiquitin ligase complex, FBXO31 variant	COMPLEXPORTAL_Complexes_07.06.2024	0.01	0.06	0.20	0.41	[−1]	Group33	75.00	3.00	[CUL1, RBX1, SKP1]
CPX:7972	SCF E3 ubiquitin ligase complex, FBXO32 variant	COMPLEXPORTAL_Complexes_07.06.2024	0.01	0.06	0.20	0.41	[−1]	Group33	75.00	3.00	[CUL1, RBX1, SKP1]
CPX:7973	SCF E3 ubiquitin ligase complex, FBXO33 variant	COMPLEXPORTAL_Complexes_07.06.2024	0.01	0.06	0.20	0.41	[−1]	Group33	75.00	3.00	[CUL1, RBX1, SKP1]
CPX:7975	SCF E3 ubiquitin ligase complex, FBXO34 variant	COMPLEXPORTAL_Complexes_07.06.2024	0.01	0.06	0.20	0.41	[−1]	Group33	75.00	3.00	[CUL1, RBX1, SKP1]
CPX:7976	SCF E3 ubiquitin ligase complex, FBXO36 variant	COMPLEXPORTAL_Complexes_07.06.2024	0.01	0.06	0.20	0.41	[−1]	Group33	75.00	3.00	[CUL1, RBX1, SKP1]
CPX:7977	SCF E3 ubiquitin ligase complex, FBXO38 variant	COMPLEXPORTAL_Complexes_07.06.2024	0.01	0.06	0.20	0.41	[−1]	Group33	75.00	3.00	[CUL1, RBX1, SKP1]
CPX:7979	SCF E3 ubiquitin ligase complex, FBXO39 variant	COMPLEXPORTAL_Complexes_07.06.2024	0.01	0.06	0.20	0.41	[−1]	Group33	75.00	3.00	[CUL1, RBX1, SKP1]
CPX:7981	SCF E3 ubiquitin ligase complex, FBXO40 variant	COMPLEXPORTAL_Complexes_07.06.2024	0.01	0.06	0.20	0.41	[−1]	Group33	75.00	3.00	[CUL1, RBX1, SKP1]
CPX:7982	SCF E3 ubiquitin ligase complex, FBXO41 variant	COMPLEXPORTAL_Complexes_07.06.2024	0.01	0.06	0.20	0.41	[−1]	Group33	75.00	3.00	[CUL1, RBX1, SKP1]
CPX:7983	SCF E3 ubiquitin ligase complex, FBXO42 variant	COMPLEXPORTAL_Complexes_07.06.2024	0.01	0.06	0.20	0.41	[−1]	Group33	75.00	3.00	[CUL1, RBX1, SKP1]
CPX:8002	SCF E3 ubiquitin ligase complex, FBXO43 variant	COMPLEXPORTAL_Complexes_07.06.2024	0.01	0.06	0.20	0.41	[−1]	Group33	75.00	3.00	[CUL1, RBX1, SKP1]
CPX:8003	SCF E3 ubiquitin ligase complex, FBXO44 variant	COMPLEXPORTAL_Complexes_07.06.2024	0.01	0.06	0.20	0.41	[−1]	Group33	75.00	3.00	[CUL1, RBX1, SKP1]
CPX:8005	SCF E3 ubiquitin ligase complex, FBXO46 variant	COMPLEXPORTAL_Complexes_07.06.2024	0.01	0.06	0.20	0.41	[−1]	Group33	75.00	3.00	[CUL1, RBX1, SKP1]
CPX:8006	SCF E3 ubiquitin ligase complex, FBXO47 variant	COMPLEXPORTAL_Complexes_07.06.2024	0.01	0.06	0.20	0.41	[−1]	Group33	75.00	3.00	[CUL1, RBX1, SKP1]
CPX:8108	SCF E3 ubiquitin ligase complex, TSPAN17 variant	COMPLEXPORTAL_Complexes_07.06.2024	0.01	0.06	0.20	0.41	[−1]	Group33	75.00	3.00	[CUL1, RBX1, SKP1]

Unlike in the canonical Tau‐F network, *VCP‐NPL4‐UFD1‐UBXN1 AAA ATPase* complex (CPX‐8105) and *VCP‐NPL4‐UFD1‐FAF2 AAA ATPase* complex (CPX‐8104) are not expressed in the P301L network, despite their critical roles in the dislocation and extraction of substrate proteins into the cytosol for proteasomal degradation (Table 4). The significance of the UBXN1 variant (CPX‐8105) has been discussed above; it is important in degrading ubiquitinated proteins and managing proteotoxic stress.

**TABLE 4:** List of complexes that are not detected in the P301L network compared to the canonical Tau‐F.

Complex AC	Complex name	GO biological process
CPX‐8104	VCP‐NPL4‐UFD1‐FAF2 AAA ATPase complex	ER‐associated protein degradation (ERAD) pathway
CPX‐8105	VCP‐NPL4‐UFD1‐UBXN1 AAA ATPase complex	Ubiquitin‐dependent protein catabolic process
CPX‐5047	Ubiquitous AP‐1 adaptor complex, sigma1a variant	Vesicle‐mediated transport, platelet dense granule organisation, melanosome assembly
CPX‐5048	Ubiquitous AP‐1 adaptor complex, sigma1b variant	Vesicle‐mediated transport, platelet dense granule organisation, melanosome assembly
CPX‐5049	Ubiquitous AP‐1 adaptor complex, sigma1c variant	Vesicle‐mediated transport, platelet dense granule organisation, melanosome assembly
CPX‐2470	Vacuolar proton translocating ATPase complex, ATP6V0A1 variant	Lysosomal lumen acidification, endosomal lumen acidification, proton transmembrane transport
CPX‐6904	Vacuolar proton translocating ATPase complex, ATP6V0A2 variant	Golgi lumen acidification, proton transmembrane transport
CPX‐6905	Vacuolar proton translocating ATPase complex, ATP6V0A3 variant	Vacuolar acidification, lysosomal lumen acidification, proton transmembrane transport
CPX‐6912	Vacuolar proton translocating ATPase complex, ATP6V0A4 variant	Proton transmembrane transport, intracellular pH reduction
CPX‐329 ^a^	ESCRT‐III complex	Multivesicular body sorting pathway, autophagosome maturation, nuclear envelope reassembly, viral budding via host ESCRT complex
CPX‐696 ^a^	PRMT5 methylosome complex, CLNS1A variant	Positive regulation of mRNA splicing, via spliceosome
CPX‐8104 ^a^	VCP‐NPL4‐UFD1‐FAF2 AAA ATPase complex	Endoplasmic‐reticulum‐associated protein degradation (ERAD) pathway
CPX‐2556 ^a^	Nucleosome, variant H3.1‐H2A.2‐H2B.1	Chromatin organisation
CPX‐2564 ^a^	Nucleosome, variant H3.1t‐H2A.2‐H2B.1	Chromatin organisation
CPX‐5668 ^a^	Nucleosome, variant H3.2‐H2A.2‐H2B.1	Chromatin organisation
CPX‐5647 ^a^	CENP‐A nucleosome complex	Protein localisation to CENP‐A containing chromatin

^a^ Complexes that are not enriched in both the mutant networks.

The *VCP‐NPL4‐UFD1‐FAF2 variant* also plays a crucial role in cellular stress responses. Gwon et al. [31] showed that stress granules, which are dynamic cytoplasmic structures formed in response to stress via liquid–liquid phase separation, typically disassemble rapidly once the stress is relieved. Impaired disassembly of these granules has been associated with diseases such as amyotrophic lateral sclerosis, frontotemporal dementia and multisystem proteinopathy. The study reveals that ubiquitinated G3BP1 interacts with the ER‐associated protein FAF2, which then recruits the ubiquitin‐dependent segregase VCP. By targeting G3BP1, the interaction network for stress granules falls below the percolation threshold necessary for phase separation, resulting in granule disassembly.

The loss of VCP‐UBXN1 results in the inappropriate stabilisation of ubiquitylated BAG6 clients, leading to their accumulation in insoluble aggregates [32]. This accumulation sensitises cells to proteotoxic stress, underscoring the significance of the VCP‐NPL4‐UFD1‐UBXN1 complex in maintaining cellular protein homeostasis.

The *Ubiquitous AP‐1 Adaptor complex* variants—*sigma1a* (CPX‐5047), *sigma1b* (CPX‐5048) and *sigma1c* (CPX‐5049)—were observed in the canonical Tau‐F network and the V337M mutant network, but were absent in the P301L mutant network. These adaptor complexes play a critical role in forming membrane coats that mediate cargo selection and vesicle budding, such as linking clathrin to the membrane surface of trans‐Golgi network (TGN) vesicles. They also recruit proteins involved in downstream vesicle functions, including motility, vesicle tethering and fusion with the target organelle. These complexes are essential for the biogenesis of specialised organelles within the endosomal‐lysosomal system, such as melanosomes and platelet dense granules. Meyer et al. [33] demonstrated that knocking down AP1S1 is embryonically lethal. In cellular models, AP1S1 knockdown leads to a failure in recycling from the TGN to endosomes. Additionally, the work by Poirier et al. [34] showed that AP1S1 knockdown results in impaired targeting of low‐density lipoprotein‐derived cholesterol and glycosphingolipids to the late endosome/lysosome.

Upon activation by BLOC‐3 (CPX‐5043), RAB32 (UniProtKB: Q13637) and RAB38 (UniProtKB: P57729) interact with AP‐3 complexes (CPX‐5051 and CPX‐5052), AP‐1 and BLOC‐2 (CPX‐5044). This implies that the absence of functional AP‐1 disrupts AP‐3‐mediated clathrin‐coated vesicle cargo loading and vesicle‐mediated transport. This finding supports the work of Salminen et al. [35], which demonstrates that impaired APP protein processing contributes to the pathogenesis of AD.

The SIGNOR (Lo Surdo et al. [36]) database shows evidence (SIGNOR‐C248) that the Ubiquitous AP‐1 Adaptor complex, sigma1a variant upregulates SORT1 (UniProtKB: Q99523), a protein that plays a crucial role in neuronal apoptosis and lysosomal protein trafficking:

- Neuronal Apoptosis: SORT1 promotes neuronal apoptosis by mediating the endocytosis of proapoptotic precursor forms of brain‐derived neurotrophic factor (proBDNF) and nerve growth factor (proNGFB). These precursor forms are known to trigger apoptotic pathways in neurons, suggesting that upregulation of SORT1 by the AP‐1 adaptor complex may contribute to apoptosis in neurodegenerative conditions.
- Protein Transport to Lysosomes: SORT1 is essential for the transport of proteins from the Golgi apparatus to lysosomes via a pathway that does not involve the mannose‐6‐phosphate receptor (M6PR). Instead, lysosomal proteins bind specifically to SORT1 in the Golgi apparatus. The receptor–ligand complex is then transported to an acidic prelysosomal compartment, where the low pH environment facilitates the dissociation of the complex, allowing the lysosomal proteins to be delivered to their target locations.

The regulation of SORT1 by the AP‐1 adaptor complex highlights the intricate mechanisms involved in cellular transport and apoptosis, especially in the context of neuronal health and neurodegenerative diseases.

*Vacuolar proton translocating ATPase* complexes—*ATP6V0A1* variant (CPX‐2470), ATP6V0A2 variant (CPX‐6904), ATP6V0A3 variant (CPX‐6905) and ATP6V0A4 variant (CPX‐6912)—are not detected in the P301L mutant network, but are enriched in the Tau‐F network. These complexes are responsible for translocating protons across a lipid bilayer via an ATP‐driven rotary mechanism, which acidifies the lumen of their resident organelles. This acidification is crucial, as membrane‐bound ion transporters and proton exchangers use the pH gradient to sequester metal ions within vacuoles and other cellular organelles. The coordinated action of V‐ATPase and these transporters is essential for maintaining cellular homeostasis.

- ATP6V0A1 variant: localises to synaptic vesicles in presynaptic neurons and to late endo/lysosomal compartments in nonneuronal tissues.
- ATP6V0A2 variant: found in the Golgi apparatus, where it contributes to organelle acidification.
- ATP6V0A3 variant: primarily localises to endolysosomal compartments and can also traffic to the plasma membrane, particularly at the ruffled border of osteoclasts.
- ATP6V0A4 variant: trafficked to the plasma membrane, specifically at the apical membrane of Type A intercalated cells, where it plays a role in acid–base regulation.

These variants collectively support diverse roles in different cellular compartments, contributing to ion homeostasis, protein degradation and synaptic vesicle function, all of which are essential processes that may be disrupted in AD.

Higashida et al. [37] provided an overview of the 16‐kDa proteolipid mediatophore, specifically the transmembrane c‐subunit of the V0 sector of the vacuolar proton ATPase (ATP6V0C), which is involved in mediating the secretion of acetylcholine. They demonstrated that acetylcholine, serotonin and dopamine (DA) are released from the cell soma and/or dendrites when ATP6V0C is expressed in cultured cells. In Parkinson‐model mice, adeno‐associated viral vector‐mediated gene transfer of ATP6V0C into the caudate putamen enhanced the depolarisation‐induced overflow of endogenous DA. Additionally, motor impairment in hemiparkinsonian model mice was alleviated when ATP6V0C was expressed along with DA‐synthesising enzymes. The study suggests potential future applications of ATP6V0C as a tool for gene therapy, cell transplantation therapy and inducible pluripotent stem cell therapy in neurological diseases.

Kim et al. [38] found that the lumenal ATP6V0C and cytosolic ATP6V1B2 subunits of the V‐ATPase complex bind to internalised Aβ and cytosolic PHF‐1‐reactive MAPT/Tau, respectively. These interactions disrupt V‐ATPase activity and endolysosomal function in vitro, ultimately inducing neurodegeneration. The authors propose that the endolysosomal V‐ATPase acts as a proteotoxic receptor that binds to pathogenic proteins, impairing endolysosomal function in AD and contributing to neurodegeneration associated with proteopathy.

##### 4.1.1.3 Complexes V337M Mutant Tau‐F Network Failed to Enrich Compared to the Tau‐F Network

**FIGURE 3:**
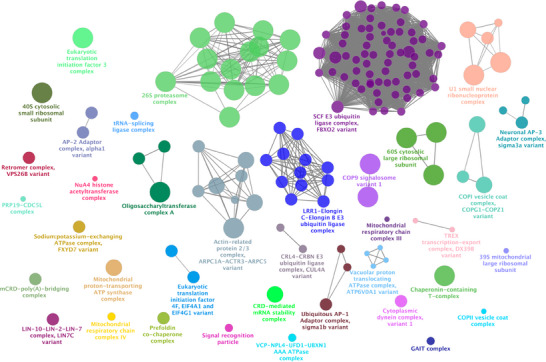
Enrichment analysis of high‐confident human proteins of V337M mutant Tau‐F for Complex Portal complexes.

In the Tau‐F network, both variants of the *sodium:potassium‐exchanging ATPase complexes* were detected, whereas the V337M mutant network did not generate the *FXYD6* variant CPX‐8144 (Figure 3, Table 5). Complete list of complexes enriched for V337M mutant Tau‐F network can be found in Table 6. Additionally, the *VCP‐NPL4‐UFD1‐FAF2 AAA ATPase* complex (CPX‐8104) was not detected in the V337M mutant network, while the *VCP‐NPL4‐UFD1‐UBXN1 AAA ATPase* complex (CPX‐8105) was present.

**TABLE 5:** Complexes not detected in the V337M Tau‐F network compared to the Tau‐F network.

Complex AC	Complex name	GO biological process
CPX‐8144	Sodium:potassium‐exchanging ATPase complex, FXYD6 variant	Cellular sodium ion homeostasis Cellular potassium ion homeostasis Establishment or maintenance of transmembrane electrochemical gradient Hydrogen ion transmembrane transport
CPX‐329 ^a^	ESCRT‐III complex	Multivesicular body sorting pathway, autophagosome maturation, nuclear envelope reassembly, viral budding via host ESCRT complex
CPX‐696 ^a^	PRMT5 methylosome complex, CLNS1A variant	Positive regulation of mRNA splicing, via spliceosome
CPX‐8104 ^a^	VCP‐NPL4‐UFD1‐FAF2 AAA ATPase complex	Endoplasmic‐reticulum‐associated protein degradation (ERAD) pathway
CPX‐2556 ^a^	Nucleosome, variant H3.1‐H2A.2‐H2B.1	Chromatin organisation
CPX‐2564 ^a^	Nucleosome, variant H3.1t‐H2A.2‐H2B.1	Chromatin organisation
CPX‐5668 ^a^	Nucleosome, variant H3.2‐H2A.2‐H2B.1	Chromatin organisation
CPX‐5647 ^a^	CENP‐A nucleosome complex	Protein localisation to CENP‐A containing chromatin

^a^ Complexes that are not enriched in both the mutant networks.

**TABLE 6:** Complex Portal complexes enriched for high‐confidence interacting proteins of mutant V337M Tau‐F.

ID	Term	Ontology source	Term *p* value	Term *p* value corrected with Bonferroni step down	Group *p* value	Group *p* value corrected with Bonferroni step down	GOLevels	GOGroups	% Associated genes	Nr. genes	Associated genes found
CPX:1076	mCRD‐poly(A)‐bridging complex	COMPLEXPORTAL_Complexes_07.06.2024	0.00	0.02	0.00	0.02	[−1]	Group00	80.00	4.00	[CSDE1, HNRNPD, PABPC1, SYNCRIP]
CPX:1080	CRD‐mediated mRNA stability complex	COMPLEXPORTAL_Complexes_07.06.2024	0.00	0.00	0.00	0.00	[−1]	Group01	100.00	5.00	[DHX9, HNRNPU, IGF2BP1, SYNCRIP, YBX1]
CPX:2360	COPII vesicle coat complex	COMPLEXPORTAL_Complexes_07.06.2024	0.10	0.67	0.10	0.67	[−1]	Group02	33.33	3.00	[SEC13, SEC24C, SEC31A]
CPX:2476	GAIT complex	COMPLEXPORTAL_Complexes_07.06.2024	0.01	0.08	0.01	0.08	[−1]	Group03	75.00	3.00	[GAPDH, RPL13A, SYNCRIP]
CPX:2652	Signal recognition particle	COMPLEXPORTAL_Complexes_07.06.2024	0.01	0.08	0.01	0.08	[−1]	Group04	57.14	4.00	[SRP14, SRP68, SRP72, SRP9]
CPX:2716	Eukaryotic translation initiation Factor 2 complex	COMPLEXPORTAL_Complexes_07.06.2024	0.00	0.03	0.00	0.03	[−1]	Group05	100.00	3.00	[EIF2S1, EIF2S2, EIF2S3]
CPX:5025	Cytoplasmic dynein complex, Variant 1	COMPLEXPORTAL_Complexes_07.06.2024	0.00	0.04	0.00	0.04	[−1]	Group06	66.67	4.00	[DYNC1H1, DYNC1I2, DYNLL1, DYNLRB1]
CPX:7843	Retromer complex, VPS26B variant	COMPLEXPORTAL_Complexes_07.06.2024	0.00	0.03	0.00	0.03	[−1]	Group07	100.00	3.00	[VPS26B, VPS29, VPS35]
CPX:5223	40S cytosolic small ribosomal subunit	COMPLEXPORTAL_Complexes_07.06.2024	0.00	0.00	0.00	0.00	[−1]	Group08	100.00	30.00	[RACK1, RPS10, RPS11, RPS12, RPS13, RPS14, RPS15, RPS15A, RPS16, RPS17, RPS18, RPS19, RPS2, RPS20, RPS21, RPS23, RPS24, RPS25, RPS26, RPS27A, RPS28, RPS29, RPS3, RPS3A, RPS5, RPS6, RPS7, RPS8, RPS9, RPSA]
CPX:5226	39S mitochondrial large ribosomal subunit	COMPLEXPORTAL_Complexes_07.06.2024	1.00	1.00	1.00	1.00	[−1]	Group09	11.32	6.00	[MRPL16, MRPL33, MRPL34, MRPL46, MRPL48, MRPL9]
CPX:560	Mitochondrial respiratory chain Complex III	COMPLEXPORTAL_Complexes_07.06.2024	0.15	0.62	0.15	0.77	[−1]	Group10	27.27	3.00	[UQCR10, UQCRC1, UQCRC2]
CPX:577	Mitochondrial respiratory chain Complex I	COMPLEXPORTAL_Complexes_07.06.2024	0.00	0.03	0.00	0.03	[−1]	Group11	29.55	13.00	[NDUFA10, NDUFA13, NDUFA5, NDUFA8, NDUFA9, NDUFAB1, NDUFB10, NDUFB3, NDUFB9, NDUFS1, NDUFS3, NDUFS4, NDUFS5]
CPX:5824	PRP19‐CDC5L complex	COMPLEXPORTAL_Complexes_07.06.2024	0.05	0.38	0.05	0.43	[−1]	Group12	42.86	3.00	[CDC5L, HSPA8, PRPF19]
CPX:6030	Chaperonin‐containing T‐complex	COMPLEXPORTAL_Complexes_07.06.2024	0.00	0.00	0.00	0.00	[−1]	Group13	100.00	8.00	[CCT2, CCT3, CCT4, CCT5, CCT6A, CCT7, CCT8, TCP1]
CPX:6036	Eukaryotic translation initiation Factor 3 complex	COMPLEXPORTAL_Complexes_07.06.2024	0.00	0.00	0.00	0.00	[−1]	Group14	100.00	13.00	[EIF3A, EIF3B, EIF3C, EIF3D, EIF3E, EIF3F, EIF3G, EIF3H, EIF3I, EIF3J, EIF3K, EIF3L, EIF3M]
CPX:6123	Mitochondrial respiratory chain Complex IV	COMPLEXPORTAL_Complexes_07.06.2024	0.00	0.06	0.00	0.06	[−1]	Group15	42.86	6.00	[COX2, COX4I1, COX5A, COX5B, COX6B1, COX6C]
CPX:6149	Prefoldin cochaperone complex	COMPLEXPORTAL_Complexes_07.06.2024	0.00	0.04	0.00	0.04	[−1]	Group16	66.67	4.00	[PFDN1, PFDN2, PFDN5, VBP1]
CPX:6151	Mitochondrial proton‐transporting ATP synthase complex	COMPLEXPORTAL_Complexes_07.06.2024	0.00	0.00	0.00	0.00	[−1]	Group17	50.00	10.00	[ATP5F1A, ATP5F1B, ATP5F1C, ATP5F1D, ATP5ME, ATP5MG, ATP5PB, ATP5PD, ATP5PF, ATP5PO]
CPX:6411	tRNA‐splicing ligase complex	COMPLEXPORTAL_Complexes_07.06.2024	0.01	0.08	0.01	0.08	[−1]	Group18	57.14	4.00	[DDX1, FAM98B, RTCB, RTRAF]
CPX:7745	LIN‐10‐LIN‐2‐LIN‐7 complex, LIN7C variant	COMPLEXPORTAL_Complexes_07.06.2024	0.00	0.03	0.00	0.03	[−1]	Group19	100.00	3.00	[APBA1, CASK, LIN7C]
CPX:8105	VCP‐NPL4‐UFD1‐UBXN1 AAA ATPase complex	COMPLEXPORTAL_Complexes_07.06.2024	0.01	0.08	0.01	0.08	[−1]	Group20	75.00	3.00	[UBXN1, UFD1, VCP]
CPX:8146	Sodium:potassium‐exchanging ATPase complex, FXYD7 variant	COMPLEXPORTAL_Complexes_07.06.2024	0.00	0.03	0.00	0.03	[−1]	Group21	100.00	3.00	[ATP1A1, ATP1B1, FXYD7]
CPX:978	NuA4 histone acetyltransferase complex	COMPLEXPORTAL_Complexes_07.06.2024	0.73	1.00	0.73	1.00	[−1]	Group22	15.79	3.00	[ACTB, RUVBL1, RUVBL2]
CPX:1870	COP9 signalosome Variant 1	COMPLEXPORTAL_Complexes_07.06.2024	0.00	0.00	0.00	0.00	[−1]	Group23	100.00	8.00	[COPS2, COPS3, COPS4, COPS5, COPS6, COPS7A, COPS8, GPS1]
CPX:1871	COP9 signalosome Variant 2	COMPLEXPORTAL_Complexes_07.06.2024	0.00	0.00	0.00	0.00	[−1]	Group23	100.00	8.00	[COPS2, COPS3, COPS4, COPS5, COPS6, COPS7B, COPS8, GPS1]
CPX:2759	CRL4‐CRBN E3 ubiquitin ligase complex, CUL4A variant	COMPLEXPORTAL_Complexes_07.06.2024	0.01	0.08	0.02	0.17	[−1]	Group24	75.00	3.00	[CRBN, DDB1, RBX1]
CPX:2762	CRL4‐CRBN E3 ubiquitin ligase complex, CUL4B variant	COMPLEXPORTAL_Complexes_07.06.2024	0.01	0.08	0.02	0.17	[−1]	Group24	75.00	3.00	[CRBN, DDB1, RBX1]
CPX:5149	AP‐2 adaptor complex, alpha1 variant	COMPLEXPORTAL_Complexes_07.06.2024	0.00	0.01	0.00	0.00	[−1]	Group25	100.00	4.00	[AP2A1, AP2B1, AP2M1, AP2S1]
CPX:5150	AP‐2 adaptor complex, alpha2 variant	COMPLEXPORTAL_Complexes_07.06.2024	0.00	0.01	0.00	0.00	[−1]	Group25	100.00	4.00	[AP2A2, AP2B1, AP2M1, AP2S1]
CPX:2666	Eukaryotic translation initiation Factor 4F, EIF4A1 and EIF4G1 variant	COMPLEXPORTAL_Complexes_07.06.2024	0.00	0.03	0.00	0.00	[−1]	Group26	100.00	3.00	[EIF4A1, EIF4E, EIF4G1]
CPX:5634	Eukaryotic translation initiation Factor 4F, EIF4A2 and EIF4G1 variant	COMPLEXPORTAL_Complexes_07.06.2024	0.00	0.03	0.00	0.00	[−1]	Group26	100.00	3.00	[EIF4A2, EIF4E, EIF4G1]
CPX:2488	TREX transcription‐export complex, DX39B variant	COMPLEXPORTAL_Complexes_07.06.2024	0.14	0.68	0.26	1.00	[−1]	Group27	25.00	4.00	[ALYREF, CHTOP, DDX39B, ERH]
CPX:7261	TREX transcription‐export complex, DX39A variant	COMPLEXPORTAL_Complexes_07.06.2024	0.45	1.00	0.26	1.00	[−1]	Group27	18.75	3.00	[ALYREF, CHTOP, ERH]
CPX:5047	Ubiquitous AP‐1 adaptor complex, sigma1a variant	COMPLEXPORTAL_Complexes_07.06.2024	0.01	0.08	0.00	0.04	[−1]	Group28	75.00	3.00	[AP1B1, AP1G1, AP1M1]
CPX:5048	Ubiquitous AP‐1 adaptor complex, sigma1b variant	COMPLEXPORTAL_Complexes_07.06.2024	0.00	0.01	0.00	0.04	[−1]	Group28	100.00	4.00	[AP1B1, AP1G1, AP1M1, AP1S2]
CPX:5049	Ubiquitous AP‐1 adaptor complex, sigma1c variant	COMPLEXPORTAL_Complexes_07.06.2024	0.01	0.08	0.00	0.04	[−1]	Group28	75.00	3.00	[AP1B1, AP1G1, AP1M1]
CPX:5051	Ubiquitous AP‐3 adaptor complex, sigma3a variant	COMPLEXPORTAL_Complexes_07.06.2024	0.01	0.08	0.00	0.01	[−1]	Group29	75.00	3.00	[AP3D1, AP3M1, AP3S1]
CPX:5053	Neuronal AP‐3 adaptor complex, sigma3b variant	COMPLEXPORTAL_Complexes_07.06.2024	0.01	0.08	0.00	0.01	[−1]	Group29	75.00	3.00	[AP3B2, AP3D1, AP3M2]
CPX:5055	Neuronal AP‐3 adaptor complex, sigma3a variant	COMPLEXPORTAL_Complexes_07.06.2024	0.00	0.01	0.00	0.01	[−1]	Group29	100.00	4.00	[AP3B2, AP3D1, AP3M2, AP3S1]
CPX:7803	COPI vesicle coat complex, COPG1‐COPZ1 variant	COMPLEXPORTAL_Complexes_07.06.2024	0.00	0.00	0.00	0.00	[−1]	Group30	85.71	6.00	[ARCN1, COPA, COPB1, COPB2, COPG1, COPZ1]
CPX:7969	COPI vesicle coat complex, COPG2‐COPZ1 variant	COMPLEXPORTAL_Complexes_07.06.2024	0.00	0.00	0.00	0.00	[−1]	Group30	85.71	6.00	[ARCN1, COPA, COPB1, COPB2, COPG2, COPZ1]
CPX:7970	COPI vesicle coat complex, COPG1‐COPZ2 variant	COMPLEXPORTAL_Complexes_07.06.2024	0.00	0.01	0.00	0.00	[−1]	Group30	71.43	5.00	[ARCN1, COPA, COPB1, COPB2, COPG1]
CPX:5183	60S cytosolic large ribosomal subunit	COMPLEXPORTAL_Complexes_07.06.2024	0.00	0.00	0.00	0.00	[−1]	Group31	65.22	30.00	[RPL11, RPL12, RPL13, RPL13A, RPL17, RPL18A, RPL21, RPL23, RPL23A, RPL24, RPL27, RPL27A, RPL28, RPL29, RPL30, RPL31, RPL32, RPL34, RPL35, RPL35A, RPL37A, RPL38, RPL5, RPL7A, RPL8, RPL9, RPLP0, RPLP1, RPLP2, UBA52]
CPX:7664	60S cytosolic large ribosomal subunit, testis‐specific variant	COMPLEXPORTAL_Complexes_07.06.2024	0.00	0.00	0.00	0.00	[−1]	Group31	65.22	30.00	[RPL11, RPL12, RPL13, RPL13A, RPL17, RPL18A, RPL21, RPL23, RPL23A, RPL24, RPL27, RPL27A, RPL28, RPL29, RPL30, RPL31, RPL32, RPL34, RPL35, RPL35A, RPL37A, RPL38, RPL5, RPL7A, RPL8, RPL9, RPLP0, RPLP1, RPLP2, UBA52]
CPX:7665	60S cytosolic large ribosomal subunit, striated muscle variant	COMPLEXPORTAL_Complexes_07.06.2024	0.00	0.00	0.00	0.00	[−1]	Group31	63.83	30.00	[RPL11, RPL12, RPL13, RPL13A, RPL17, RPL18A, RPL21, RPL23, RPL23A, RPL24, RPL27, RPL27A, RPL28, RPL29, RPL30, RPL31, RPL32, RPL34, RPL35, RPL35A, RPL37A, RPL38, RPL5, RPL7A, RPL8, RPL9, RPLP0, RPLP1, RPLP2, UBA52]
CPX:5621	Oligosaccharyltransferase Complex A	COMPLEXPORTAL_Complexes_07.06.2024	0.00	0.00	0.00	0.00	[−1]	Group32	77.78	7.00	[DAD1, DDOST, OSTC, RPN1, RPN2, STT3A, TMEM258]
CPX:5622	Oligosaccharyltransferase Complex B, MAGT1 variant	COMPLEXPORTAL_Complexes_07.06.2024	0.00	0.02	0.00	0.00	[−1]	Group32	62.50	5.00	[DAD1, DDOST, RPN1, RPN2, TMEM258]
CPX:8738	Oligosaccharyltransferase Complex B, TUCS3 variant	COMPLEXPORTAL_Complexes_07.06.2024	0.00	0.02	0.00	0.00	[−1]	Group32	62.50	5.00	[DAD1, DDOST, RPN1, RPN2, TMEM258]
CPX:2470	Vacuolar proton translocating ATPase complex, ATP6V0A1 variant	COMPLEXPORTAL_Complexes_07.06.2024	0.03	0.26	0.07	0.58	[−1]	Group33	40.00	4.00	[ATP6V0A1, ATP6V0C, ATP6V1A, ATP6V1D]
CPX:6904	Vacuolar proton translocating ATPase complex, ATP6V0A2 variant	COMPLEXPORTAL_Complexes_07.06.2024	0.12	0.74	0.07	0.58	[−1]	Group33	30.00	3.00	[ATP6V0C, ATP6V1A, ATP6V1D]
CPX:6905	Vacuolar proton translocating ATPase complex, ATP6V0A3 variant	COMPLEXPORTAL_Complexes_07.06.2024	0.12	0.74	0.07	0.58	[−1]	Group33	30.00	3.00	[ATP6V0C, ATP6V1A, ATP6V1D]
CPX:6912	Vacuolar proton translocating ATPase complex, ATP6V0A4 variant	COMPLEXPORTAL_Complexes_07.06.2024	0.12	0.74	0.07	0.58	[−1]	Group33	30.00	3.00	[ATP6V0C, ATP6V1A, ATP6V1D]
CPX:2391	U4/U6.U5 small nuclear ribonucleoprotein complex	COMPLEXPORTAL_Complexes_07.06.2024	0.00	0.01	0.00	0.00	[−1]	Group34	37.50	12.00	[LSM3, LSM4, PRPF31, SART1, SNRPA, SNRPD1, SNRPD2, SNRPD3, SNRPE, SNRPF, SNRPG, SNU13]
CPX:2392	U1 small nuclear ribonucleoprotein complex	COMPLEXPORTAL_Complexes_07.06.2024	0.00	0.00	0.00	0.00	[−1]	Group34	90.00	9.00	[SNRNP70, SNRPA, SNRPC, SNRPD1, SNRPD2, SNRPD3, SNRPE, SNRPF, SNRPG]
CPX:2539	U2 small nuclear ribonucleoprotein complex	COMPLEXPORTAL_Complexes_07.06.2024	0.00	0.00	0.00	0.00	[−1]	Group34	52.00	13.00	[DDX46, HTATSF1, SF3A1, SF3A2, SF3B3, SF3B6, SNRPB2, SNRPD1, SNRPD2, SNRPD3, SNRPE, SNRPF, SNRPG]
CPX:2705	U7 small nuclear ribonucleoprotein complex	COMPLEXPORTAL_Complexes_07.06.2024	0.01	0.08	0.00	0.00	[−1]	Group34	57.14	4.00	[SNRPD3, SNRPE, SNRPF, SNRPG]
CPX:6033	Sm complex	COMPLEXPORTAL_Complexes_07.06.2024	0.00	0.00	0.00	0.00	[−1]	Group34	85.71	6.00	[SNRPD1, SNRPD2, SNRPD3, SNRPE, SNRPF, SNRPG]
CPX:2490	Actin‐related protein 2/3 complex, ARPC1A‐ACTR3B‐ARPC5 variant	COMPLEXPORTAL_Complexes_07.06.2024	0.00	0.00	0.00	0.00	[−1]	Group35	85.71	6.00	[ACTR2, ARPC1A, ARPC2, ARPC3, ARPC4, ARPC5]
CPX:2579	Actin‐related protein 2/3 complex, ARPC1B‐ACTR3‐ARPC5 variant	COMPLEXPORTAL_Complexes_07.06.2024	0.00	0.00	0.00	0.00	[−1]	Group35	85.71	6.00	[ACTR2, ACTR3, ARPC2, ARPC3, ARPC4, ARPC5]
CPX:2580	Actin‐related protein 2/3 complex, ARPC1B‐ACTR3B‐ARPC5L variant	COMPLEXPORTAL_Complexes_07.06.2024	0.01	0.08	0.00	0.00	[−1]	Group35	57.14	4.00	[ACTR2, ARPC2, ARPC3, ARPC4]
CPX:2583	Actin‐related protein 2/3 complex, ARPC1B‐ACTR3B‐ARPC5 variant	COMPLEXPORTAL_Complexes_07.06.2024	0.00	0.01	0.00	0.00	[−1]	Group35	71.43	5.00	[ACTR2, ARPC2, ARPC3, ARPC4, ARPC5]
CPX:2586	Actin‐related protein 2/3 complex, ARPC1A‐ACTR3‐ARPC5 variant	COMPLEXPORTAL_Complexes_07.06.2024	0.00	0.00	0.00	0.00	[−1]	Group35	100.00	7.00	[ACTR2, ACTR3, ARPC1A, ARPC2, ARPC3, ARPC4, ARPC5]
CPX:2592	Actin‐related protein 2/3 complex, ARPC1A‐ACTR3‐ARPC5L variant	COMPLEXPORTAL_Complexes_07.06.2024	0.00	0.00	0.00	0.00	[−1]	Group35	85.71	6.00	[ACTR2, ACTR3, ARPC1A, ARPC2, ARPC3, ARPC4]
CPX:2663	Actin‐related protein 2/3 complex, ARPC1B‐ACTR3‐ARPC5L variant	COMPLEXPORTAL_Complexes_07.06.2024	0.00	0.01	0.00	0.00	[−1]	Group35	71.43	5.00	[ACTR2, ACTR3, ARPC2, ARPC3, ARPC4]
CPX:2668	Actin‐related protein 2/3 complex, ARPC1B‐ACTR3B‐ARPC5L variant	COMPLEXPORTAL_Complexes_07.06.2024	0.00	0.01	0.00	0.00	[−1]	Group35	71.43	5.00	[ACTR2, ARPC1A, ARPC2, ARPC3, ARPC4]
CPX:2214	LRR1‐Elongin C‐Elongin B E3 ubiquitin ligase complex	COMPLEXPORTAL_Complexes_07.06.2024	0.00	0.02	0.14	0.81	[−1]	Group36	80.00	4.00	[CUL2, ELOB, ELOC, RBX1]
CPX:2217	FEM1A‐Elongin C‐Elongin B E3 ubiquitin ligase complex	COMPLEXPORTAL_Complexes_07.06.2024	0.00	0.02	0.14	0.81	[−1]	Group36	80.00	4.00	[CUL2, ELOB, ELOC, RBX1]
CPX:2218	FEB1B‐Elongin C‐Elongin B E3 ubiquitin ligase complex	COMPLEXPORTAL_Complexes_07.06.2024	0.00	0.02	0.14	0.81	[−1]	Group36	80.00	4.00	[CUL2, ELOB, ELOC, RBX1]
CPX:2219	FEB1C‐Elongin C‐Elongin B E3 ubiquitin ligase complex	COMPLEXPORTAL_Complexes_07.06.2024	0.00	0.02	0.14	0.81	[−1]	Group36	80.00	4.00	[CUL2, ELOB, ELOC, RBX1]
CPX:2220	ZYG11B‐Elongin C‐Elongin B E3 ubiquitin ligase complex	COMPLEXPORTAL_Complexes_07.06.2024	0.00	0.02	0.14	0.81	[−1]	Group36	80.00	4.00	[CUL2, ELOB, ELOC, RBX1]
CPX:2221	APPBP2‐Elongin C‐Elongin B E3 ubiquitin ligase complex	COMPLEXPORTAL_Complexes_07.06.2024	0.00	0.02	0.14	0.81	[−1]	Group36	80.00	4.00	[CUL2, ELOB, ELOC, RBX1]
CPX:2222	ZER1‐Elongin C‐Elongin B E3 ubiquitin ligase complex	COMPLEXPORTAL_Complexes_07.06.2024	0.00	0.02	0.14	0.81	[−1]	Group36	80.00	4.00	[CUL2, ELOB, ELOC, RBX1]
CPX:2223	KLHDC10‐Elongin C‐Elongin B E3 ubiquitin ligase complex	COMPLEXPORTAL_Complexes_07.06.2024	0.00	0.02	0.14	0.81	[−1]	Group36	80.00	4.00	[CUL2, ELOB, ELOC, RBX1]
CPX:2226	KLHDC2‐Elongin C‐Elongin B E3 ubiquitin ligase complex	COMPLEXPORTAL_Complexes_07.06.2024	0.00	0.02	0.14	0.81	[−1]	Group36	80.00	4.00	[CUL2, ELOB, ELOC, RBX1]
CPX:2228	KLHDC3‐Elongin C‐Elongin B E3 ubiquitin ligase complex	COMPLEXPORTAL_Complexes_07.06.2024	0.00	0.02	0.14	0.81	[−1]	Group36	80.00	4.00	[CUL2, ELOB, ELOC, RBX1]
CPX:2229	PRAME‐Elongin C‐Elongin B E3 ubiquitin ligase complex	COMPLEXPORTAL_Complexes_07.06.2024	0.00	0.02	0.14	0.81	[−1]	Group36	80.00	4.00	[CUL2, ELOB, ELOC, RBX1]
CPX:2250	VHL‐Elongin C‐Elongin B E3 ubiquitin ligase complex	COMPLEXPORTAL_Complexes_07.06.2024	0.00	0.02	0.14	0.81	[−1]	Group36	80.00	4.00	[CUL2, ELOB, ELOC, RBX1]
CPX:5993	26S proteasome complex	COMPLEXPORTAL_Complexes_07.06.2024	0.00	0.00	0.00	0.00	[−1]	Group37	96.97	32.00	[ADRM1, PSMA1, PSMA2, PSMA3, PSMA4, PSMA5, PSMA6, PSMA7, PSMB1, PSMB2, PSMB3, PSMB4, PSMB5, PSMB6, PSMB7, PSMC1, PSMC2, PSMC3, PSMC4, PSMC5, PSMC6, PSMD1, PSMD11, PSMD12, PSMD13, PSMD14, PSMD2, PSMD3, PSMD4, PSMD6, PSMD7, PSMD8]
CPX:8806	20S proteasome complex	COMPLEXPORTAL_Complexes_07.06.2024	0.00	0.00	0.00	0.00	[−1]	Group37	100.00	14.00	[PSMA1, PSMA2, PSMA3, PSMA4, PSMA5, PSMA6, PSMA7, PSMB1, PSMB2, PSMB3, PSMB4, PSMB5, PSMB6, PSMB7]
CPX:8841	PA200‐20S single‐capped proteasome	COMPLEXPORTAL_Complexes_07.06.2024	0.00	0.00	0.00	0.00	[−1]	Group37	93.33	14.00	[PSMA1, PSMA2, PSMA3, PSMA4, PSMA5, PSMA6, PSMA7, PSMB1, PSMB2, PSMB3, PSMB4, PSMB5, PSMB6, PSMB7]
CPX:8842	PA28‐alphabeta double‐capped 20S proteasome complex	COMPLEXPORTAL_Complexes_07.06.2024	0.00	0.00	0.00	0.00	[−1]	Group37	93.75	15.00	[PSMA1, PSMA2, PSMA3, PSMA4, PSMA5, PSMA6, PSMA7, PSMB1, PSMB2, PSMB3, PSMB4, PSMB5, PSMB6, PSMB7, PSME1]
CPX:8964	19S proteasome regulatory complex	COMPLEXPORTAL_Complexes_07.06.2024	0.00	0.00	0.00	0.00	[−1]	Group37	94.74	18.00	[ADRM1, PSMC1, PSMC2, PSMC3, PSMC4, PSMC5, PSMC6, PSMD1, PSMD11, PSMD12, PSMD13, PSMD14, PSMD2, PSMD3, PSMD4, PSMD6, PSMD7, PSMD8]
CPX:9001	PA28‐gamma single‐capped 20S proteasome complex	COMPLEXPORTAL_Complexes_07.06.2024	0.00	0.00	0.00	0.00	[−1]	Group37	93.33	14.00	[PSMA1, PSMA2, PSMA3, PSMA4, PSMA5, PSMA6, PSMA7, PSMB1, PSMB2, PSMB3, PSMB4, PSMB5, PSMB6, PSMB7]
CPX:9002	PA28‐alphabeta single‐capped 20S proteasome complex	COMPLEXPORTAL_Complexes_07.06.2024	0.00	0.00	0.00	0.00	[−1]	Group37	93.75	15.00	[PSMA1, PSMA2, PSMA3, PSMA4, PSMA5, PSMA6, PSMA7, PSMB1, PSMB2, PSMB3, PSMB4, PSMB5, PSMB6, PSMB7, PSME1]
CPX:9003	20S immunoproteasome complex	COMPLEXPORTAL_Complexes_07.06.2024	0.00	0.00	0.00	0.00	[−1]	Group37	78.57	11.00	[PSMA1, PSMA2, PSMA3, PSMA4, PSMA5, PSMA6, PSMA7, PSMB1, PSMB2, PSMB3, PSMB4]
CPX:9004	20S thymoproteasome complex	COMPLEXPORTAL_Complexes_07.06.2024	0.00	0.00	0.00	0.00	[−1]	Group37	78.57	11.00	[PSMA1, PSMA2, PSMA3, PSMA4, PSMA5, PSMA6, PSMA7, PSMB1, PSMB2, PSMB3, PSMB4]
CPX:9021	20S spermatoproteasome complex	COMPLEXPORTAL_Complexes_07.06.2024	0.00	0.00	0.00	0.00	[−1]	Group37	92.86	13.00	[PSMA1, PSMA2, PSMA3, PSMA4, PSMA5, PSMA6, PSMB1, PSMB2, PSMB3, PSMB4, PSMB5, PSMB6, PSMB7]
CPX:9022	PA28‐gamma double‐capped 20S proteasome complex	COMPLEXPORTAL_Complexes_07.06.2024	0.00	0.00	0.00	0.00	[−1]	Group37	93.33	14.00	[PSMA1, PSMA2, PSMA3, PSMA4, PSMA5, PSMA6, PSMA7, PSMB1, PSMB2, PSMB3, PSMB4, PSMB5, PSMB6, PSMB7]
CPX:9063	PA200‐20S‐PA200 double‐capped proteasome complex	COMPLEXPORTAL_Complexes_07.06.2024	0.00	0.00	0.00	0.00	[−1]	Group37	93.33	14.00	[PSMA1, PSMA2, PSMA3, PSMA4, PSMA5, PSMA6, PSMA7, PSMB1, PSMB2, PSMB3, PSMB4, PSMB5, PSMB6, PSMB7]
CPX:9082	19S‐20S‐PA28‐alphabeta hybrid proteasome complex	COMPLEXPORTAL_Complexes_07.06.2024	0.00	0.00	0.00	0.00	[−1]	Group37	94.29	33.00	[ADRM1, PSMA1, PSMA2, PSMA3, PSMA4, PSMA5, PSMA6, PSMA7, PSMB1, PSMB2, PSMB3, PSMB4, PSMB5, PSMB6, PSMB7, PSMC1, PSMC2, PSMC3, PSMC4, PSMC5, PSMC6, PSMD1, PSMD11, PSMD12, PSMD13, PSMD14, PSMD2, PSMD3, PSMD4, PSMD6, PSMD7, PSMD8, PSME1]
CPX:9085	19S‐20S‐PA28‐gamma hybrid proteasome complex	COMPLEXPORTAL_Complexes_07.06.2024	0.00	0.00	0.00	0.00	[−1]	Group37	94.12	32.00	[ADRM1, PSMA1, PSMA2, PSMA3, PSMA4, PSMA5, PSMA6, PSMA7, PSMB1, PSMB2, PSMB3, PSMB4, PSMB5, PSMB6, PSMB7, PSMC1, PSMC2, PSMC3, PSMC4, PSMC5, PSMC6, PSMD1, PSMD11, PSMD12, PSMD13, PSMD14, PSMD2, PSMD3, PSMD4, PSMD6, PSMD7, PSMD8]
CPX:9086	30S proteasome complex	COMPLEXPORTAL_Complexes_07.06.2024	0.00	0.00	0.00	0.00	[−1]	Group37	96.97	32.00	[ADRM1, PSMA1, PSMA2, PSMA3, PSMA4, PSMA5, PSMA6, PSMA7, PSMB1, PSMB2, PSMB3, PSMB4, PSMB5, PSMB6, PSMB7, PSMC1, PSMC2, PSMC3, PSMC4, PSMC5, PSMC6, PSMD1, PSMD11, PSMD12, PSMD13, PSMD14, PSMD2, PSMD3, PSMD4, PSMD6, PSMD7, PSMD8]
CPX:2239	SCF E3 ubiquitin ligase complex, FBXL5 variant	COMPLEXPORTAL_Complexes_07.06.2024	0.01	0.08	0.37	1.00	[−1]	Group38	75.00	3.00	[CUL1, RBX1, SKP1]
CPX:2241	SCF E3 ubiquitin ligase complex, FBXL13 variant	COMPLEXPORTAL_Complexes_07.06.2024	0.01	0.08	0.37	1.00	[−1]	Group38	75.00	3.00	[CUL1, RBX1, SKP1]
CPX:2319	SCF E3 ubiquitin ligase complex, FBXL14 variant	COMPLEXPORTAL_Complexes_07.06.2024	0.01	0.08	0.37	1.00	[−1]	Group38	75.00	3.00	[CUL1, RBX1, SKP1]
CPX:2343	SCF E3 ubiquitin ligase complex, FBXL16 variant	COMPLEXPORTAL_Complexes_07.06.2024	0.01	0.08	0.37	1.00	[−1]	Group38	75.00	3.00	[CUL1, RBX1, SKP1]
CPX:2365	SCF E3 ubiquitin ligase complex, BTRC variant	COMPLEXPORTAL_Complexes_07.06.2024	0.01	0.08	0.37	1.00	[−1]	Group38	75.00	3.00	[CUL1, RBX1, SKP1]
CPX:2438	SCF E3 ubiquitin ligase complex, FBXL8 variant	COMPLEXPORTAL_Complexes_07.06.2024	0.01	0.08	0.37	1.00	[−1]	Group38	75.00	3.00	[CUL1, RBX1, SKP1]
CPX:2489	SCF E3 ubiquitin ligase complex, FBXL22 variant	COMPLEXPORTAL_Complexes_07.06.2024	0.01	0.08	0.37	1.00	[−1]	Group38	75.00	3.00	[CUL1, RBX1, SKP1]
CPX:2492	SCF E3 ubiquitin ligase complex, FBXL15 variant	COMPLEXPORTAL_Complexes_07.06.2024	0.01	0.08	0.37	1.00	[−1]	Group38	75.00	3.00	[CUL1, RBX1, SKP1]
CPX:2512	SCF E3 ubiquitin ligase complex, FBXL4 variant	COMPLEXPORTAL_Complexes_07.06.2024	0.01	0.08	0.37	1.00	[−1]	Group38	75.00	3.00	[CUL1, RBX1, SKP1]
CPX:2516	SCF E3 ubiquitin ligase complex, FBXL6 variant	COMPLEXPORTAL_Complexes_07.06.2024	0.01	0.08	0.37	1.00	[−1]	Group38	75.00	3.00	[CUL1, RBX1, SKP1]
CPX:2538	SCF E3 ubiquitin ligase complex, KDM2A variant	COMPLEXPORTAL_Complexes_07.06.2024	0.01	0.08	0.37	1.00	[−1]	Group38	75.00	3.00	[CUL1, RBX1, SKP1]
CPX:2553	SCF E3 ubiquitin ligase complex, FBXL18 variant	COMPLEXPORTAL_Complexes_07.06.2024	0.00	0.03	0.37	1.00	[−1]	Group38	100.00	3.00	[CUL1, RBX1, SKP1]
CPX:2554	SCF E3 ubiquitin ligase complex, FBXL19 variant	COMPLEXPORTAL_Complexes_07.06.2024	0.01	0.08	0.37	1.00	[−1]	Group38	75.00	3.00	[CUL1, RBX1, SKP1]
CPX:2658	SCF E3 ubiquitin ligase complex, FBXL12 variant	COMPLEXPORTAL_Complexes_07.06.2024	0.01	0.08	0.37	1.00	[−1]	Group38	75.00	3.00	[CUL1, RBX1, SKP1]
CPX:2683	SCF E3 ubiquitin ligase complex, FBXL7 variant	COMPLEXPORTAL_Complexes_07.06.2024	0.01	0.08	0.37	1.00	[−1]	Group38	75.00	3.00	[CUL1, RBX1, SKP1]
CPX:2748	SCF E3 ubiquitin ligase complex, FBXL21 variant	COMPLEXPORTAL_Complexes_07.06.2024	0.00	0.03	0.37	1.00	[−1]	Group38	100.00	3.00	[CUL1, RBX1, SKP1]
CPX:2773	SCF E3 ubiquitin ligase complex, FBXL17 variant	COMPLEXPORTAL_Complexes_07.06.2024	0.01	0.08	0.37	1.00	[−1]	Group38	75.00	3.00	[CUL1, RBX1, SKP1]
CPX:2832	SCF E3 ubiquitin ligase complex, KDM2B variant	COMPLEXPORTAL_Complexes_07.06.2024	0.01	0.08	0.37	1.00	[−1]	Group38	75.00	3.00	[CUL1, RBX1, SKP1]
CPX:2873	SCF E3 ubiquitin ligase complex, FBXL20 variant	COMPLEXPORTAL_Complexes_07.06.2024	0.01	0.08	0.37	1.00	[−1]	Group38	75.00	3.00	[CUL1, RBX1, SKP1]
CPX:3291	SCF E3 ubiquitin ligase complex, FBXL3 variant	COMPLEXPORTAL_Complexes_07.06.2024	0.01	0.08	0.37	1.00	[−1]	Group38	75.00	3.00	[CUL1, RBX1, SKP1]
CPX:3292	SCF E3 ubiquitin ligase complex, FBXL2 variant	COMPLEXPORTAL_Complexes_07.06.2024	0.01	0.08	0.37	1.00	[−1]	Group38	75.00	3.00	[CUL1, RBX1, SKP1]
CPX:3295	SCF E3 ubiquitin ligase complex, SKP2 variant	COMPLEXPORTAL_Complexes_07.06.2024	0.01	0.08	0.37	1.00	[−1]	Group38	75.00	3.00	[CUL1, RBX1, SKP1]
CPX:7747	SCF E3 ubiquitin ligase complex, FBXW2 variant	COMPLEXPORTAL_Complexes_07.06.2024	0.01	0.08	0.37	1.00	[−1]	Group38	75.00	3.00	[CUL1, RBX1, SKP1]
CPX:7761	SCF E3 ubiquitin ligase complex, FBXW4 variant	COMPLEXPORTAL_Complexes_07.06.2024	0.01	0.08	0.37	1.00	[−1]	Group38	75.00	3.00	[CUL1, RBX1, SKP1]
CPX:7762	SCF E3 ubiquitin ligase complex, FBXW5 variant	COMPLEXPORTAL_Complexes_07.06.2024	0.01	0.08	0.37	1.00	[−1]	Group38	75.00	3.00	[CUL1, RBX1, SKP1]
CPX:7763	SCF E3 ubiquitin ligase complex, FBXW7 variant	COMPLEXPORTAL_Complexes_07.06.2024	0.01	0.08	0.37	1.00	[−1]	Group38	75.00	3.00	[CUL1, RBX1, SKP1]
CPX:7784	SCF E3 ubiquitin ligase complex, FBXW8‐CUL7 variant	COMPLEXPORTAL_Complexes_07.06.2024	0.02	0.17	0.37	1.00	[−1]	Group38	60.00	3.00	[CUL1, RBX1, SKP1]
CPX:7785	SCF E3 ubiquitin ligase complex, FBXW9 variant	COMPLEXPORTAL_Complexes_07.06.2024	0.01	0.08	0.37	1.00	[−1]	Group38	75.00	3.00	[CUL1, RBX1, SKP1]
CPX:7786	SCF E3 ubiquitin ligase complex, FBXW10 variant	COMPLEXPORTAL_Complexes_07.06.2024	0.01	0.08	0.37	1.00	[−1]	Group38	75.00	3.00	[CUL1, RBX1, SKP1]
CPX:7821	SCF E3 ubiquitin ligase complex, FBXW11 variant	COMPLEXPORTAL_Complexes_07.06.2024	0.01	0.08	0.37	1.00	[−1]	Group38	75.00	3.00	[CUL1, RBX1, SKP1]
CPX:7822	SCF E3 ubiquitin ligase complex, FBXW12 variant	COMPLEXPORTAL_Complexes_07.06.2024	0.01	0.08	0.37	1.00	[−1]	Group38	75.00	3.00	[CUL1, RBX1, SKP1]
CPX:7846	SCF E3 ubiquitin ligase complex, CCNF variant	COMPLEXPORTAL_Complexes_07.06.2024	0.01	0.08	0.37	1.00	[−1]	Group38	75.00	3.00	[CUL1, RBX1, SKP1]
CPX:7847	SCF E3 ubiquitin ligase complex, FBXO2 variant	COMPLEXPORTAL_Complexes_07.06.2024	0.00	0.01	0.37	1.00	[−1]	Group38	100.00	4.00	[CUL1, FBXO2, RBX1, SKP1]
CPX:7881	SCF E3 ubiquitin ligase complex, FBXO3 variant	COMPLEXPORTAL_Complexes_07.06.2024	0.01	0.08	0.37	1.00	[−1]	Group38	75.00	3.00	[CUL1, RBX1, SKP1]
CPX:7882	SCF E3 ubiquitin ligase complex, FBXO4 variant	COMPLEXPORTAL_Complexes_07.06.2024	0.01	0.08	0.37	1.00	[−1]	Group38	75.00	3.00	[CUL1, RBX1, SKP1]
CPX:7904	SCF E3 ubiquitin ligase complex, FBXO5 variant	COMPLEXPORTAL_Complexes_07.06.2024	0.01	0.08	0.37	1.00	[−1]	Group38	75.00	3.00	[CUL1, RBX1, SKP1]
CPX:7905	SCF E3 ubiquitin ligase complex, FBXO6 variant	COMPLEXPORTAL_Complexes_07.06.2024	0.01	0.08	0.37	1.00	[−1]	Group38	75.00	3.00	[CUL1, RBX1, SKP1]
CPX:7906	SCF E3 ubiquitin ligase complex, FBXO7 variant	COMPLEXPORTAL_Complexes_07.06.2024	0.01	0.08	0.37	1.00	[−1]	Group38	75.00	3.00	[CUL1, RBX1, SKP1]
CPX:7921	SCF E3 ubiquitin ligase complex, FBXO8 variant	COMPLEXPORTAL_Complexes_07.06.2024	0.01	0.08	0.37	1.00	[−1]	Group38	75.00	3.00	[CUL1, RBX1, SKP1]
CPX:7922	SCF E3 ubiquitin ligase complex, FBXO9 variant	COMPLEXPORTAL_Complexes_07.06.2024	0.01	0.08	0.37	1.00	[−1]	Group38	75.00	3.00	[CUL1, RBX1, SKP1]
CPX:7923	SCF E3 ubiquitin ligase complex, FBXO10 variant	COMPLEXPORTAL_Complexes_07.06.2024	0.01	0.08	0.37	1.00	[−1]	Group38	75.00	3.00	[CUL1, RBX1, SKP1]
CPX:7924	SCF E3 ubiquitin ligase complex, FBXO11 variant	COMPLEXPORTAL_Complexes_07.06.2024	0.01	0.08	0.37	1.00	[−1]	Group38	75.00	3.00	[CUL1, RBX1, SKP1]
CPX:7925	SCF E3 ubiquitin ligase complex, FBXO15 variant	COMPLEXPORTAL_Complexes_07.06.2024	0.01	0.08	0.37	1.00	[−1]	Group38	75.00	3.00	[CUL1, RBX1, SKP1]
CPX:7926	SCF E3 ubiquitin ligase complex, FBXO16 variant	COMPLEXPORTAL_Complexes_07.06.2024	0.01	0.08	0.37	1.00	[−1]	Group38	75.00	3.00	[CUL1, RBX1, SKP1]
CPX:7927	SCF E3 ubiquitin ligase complex, FBXO17 variant	COMPLEXPORTAL_Complexes_07.06.2024	0.01	0.08	0.37	1.00	[−1]	Group38	75.00	3.00	[CUL1, RBX1, SKP1]
CPX:7928	SCF E3 ubiquitin ligase complex, FBH1 variant	COMPLEXPORTAL_Complexes_07.06.2024	0.01	0.08	0.37	1.00	[−1]	Group38	75.00	3.00	[CUL1, RBX1, SKP1]
CPX:7929	SCF E3 ubiquitin ligase complex, LMO7 variant	COMPLEXPORTAL_Complexes_07.06.2024	0.01	0.08	0.37	1.00	[−1]	Group38	75.00	3.00	[CUL1, RBX1, SKP1]
CPX:7930	SCF E3 ubiquitin ligase complex, FBXO21 variant	COMPLEXPORTAL_Complexes_07.06.2024	0.00	0.01	0.37	1.00	[−1]	Group38	100.00	4.00	[CUL1, FBXO21, RBX1, SKP1]
CPX:7962	SCF E3 ubiquitin ligase complex, FBXO22 variant	COMPLEXPORTAL_Complexes_07.06.2024	0.01	0.08	0.37	1.00	[−1]	Group38	75.00	3.00	[CUL1, RBX1, SKP1]
CPX:7963	SCF E3 ubiquitin ligase complex, FBXO24 variant	COMPLEXPORTAL_Complexes_07.06.2024	0.01	0.08	0.37	1.00	[−1]	Group38	75.00	3.00	[CUL1, RBX1, SKP1]
CPX:7965	SCF E3 ubiquitin ligase complex, FBXO25 variant	COMPLEXPORTAL_Complexes_07.06.2024	0.01	0.08	0.37	1.00	[−1]	Group38	75.00	3.00	[CUL1, RBX1, SKP1]
CPX:7966	SCF E3 ubiquitin ligase complex, FBXO27 variant	COMPLEXPORTAL_Complexes_07.06.2024	0.01	0.08	0.37	1.00	[−1]	Group38	75.00	3.00	[CUL1, RBX1, SKP1]
CPX:7967	SCF E3 ubiquitin ligase complex, FBXO28 variant	COMPLEXPORTAL_Complexes_07.06.2024	0.01	0.08	0.37	1.00	[−1]	Group38	75.00	3.00	[CUL1, RBX1, SKP1]
CPX:7968	SCF E3 ubiquitin ligase complex, FBXO30 variant	COMPLEXPORTAL_Complexes_07.06.2024	0.00	0.01	0.37	1.00	[−1]	Group38	100.00	4.00	[CUL1, FBXO30, RBX1, SKP1]
CPX:7971	SCF E3 ubiquitin ligase complex, FBXO31 variant	COMPLEXPORTAL_Complexes_07.06.2024	0.01	0.08	0.37	1.00	[−1]	Group38	75.00	3.00	[CUL1, RBX1, SKP1]
CPX:7972	SCF E3 ubiquitin ligase complex, FBXO32 variant	COMPLEXPORTAL_Complexes_07.06.2024	0.01	0.08	0.37	1.00	[−1]	Group38	75.00	3.00	[CUL1, RBX1, SKP1]
CPX:7973	SCF E3 ubiquitin ligase complex, FBXO33 variant	COMPLEXPORTAL_Complexes_07.06.2024	0.01	0.08	0.37	1.00	[−1]	Group38	75.00	3.00	[CUL1, RBX1, SKP1]
CPX:7975	SCF E3 ubiquitin ligase complex, FBXO34 variant	COMPLEXPORTAL_Complexes_07.06.2024	0.01	0.08	0.37	1.00	[−1]	Group38	75.00	3.00	[CUL1, RBX1, SKP1]
CPX:7976	SCF E3 ubiquitin ligase complex, FBXO36 variant	COMPLEXPORTAL_Complexes_07.06.2024	0.01	0.08	0.37	1.00	[−1]	Group38	75.00	3.00	[CUL1, RBX1, SKP1]
CPX:7977	SCF E3 ubiquitin ligase complex, FBXO38 variant	COMPLEXPORTAL_Complexes_07.06.2024	0.01	0.08	0.37	1.00	[−1]	Group38	75.00	3.00	[CUL1, RBX1, SKP1]
CPX:7979	SCF E3 ubiquitin ligase complex, FBXO39 variant	COMPLEXPORTAL_Complexes_07.06.2024	0.01	0.08	0.37	1.00	[−1]	Group38	75.00	3.00	[CUL1, RBX1, SKP1]
CPX:7981	SCF E3 ubiquitin ligase complex, FBXO40 variant	COMPLEXPORTAL_Complexes_07.06.2024	0.01	0.08	0.37	1.00	[−1]	Group38	75.00	3.00	[CUL1, RBX1, SKP1]
CPX:7982	SCF E3 ubiquitin ligase complex, FBXO41 variant	COMPLEXPORTAL_Complexes_07.06.2024	0.01	0.08	0.37	1.00	[−1]	Group38	75.00	3.00	[CUL1, RBX1, SKP1]
CPX:7983	SCF E3 ubiquitin ligase complex, FBXO42 variant	COMPLEXPORTAL_Complexes_07.06.2024	0.01	0.08	0.37	1.00	[−1]	Group38	75.00	3.00	[CUL1, RBX1, SKP1]
CPX:8002	SCF E3 ubiquitin ligase complex, FBXO43 variant	COMPLEXPORTAL_Complexes_07.06.2024	0.01	0.08	0.37	1.00	[−1]	Group38	75.00	3.00	[CUL1, RBX1, SKP1]
CPX:8003	SCF E3 ubiquitin ligase complex, FBXO44 variant	COMPLEXPORTAL_Complexes_07.06.2024	0.01	0.08	0.37	1.00	[−1]	Group38	75.00	3.00	[CUL1, RBX1, SKP1]
CPX:8005	SCF E3 ubiquitin ligase complex, FBXO46 variant	COMPLEXPORTAL_Complexes_07.06.2024	0.01	0.08	0.37	1.00	[−1]	Group38	75.00	3.00	[CUL1, RBX1, SKP1]
CPX:8006	SCF E3 ubiquitin ligase complex, FBXO47 variant	COMPLEXPORTAL_Complexes_07.06.2024	0.01	0.08	0.37	1.00	[−1]	Group38	75.00	3.00	[CUL1, RBX1, SKP1]
CPX:8108	SCF E3 ubiquitin ligase complex, TSPAN17 variant	COMPLEXPORTAL_Complexes_07.06.2024	0.01	0.08	0.37	1.00	[−1]	Group38	75.00	3.00	[CUL1, RBX1, SKP1]

##### 4.1.1.4 Complexes Enriched Only in V337M or P301L Mutant Tau‐F Networks

The *PRP19‐CDC5L* (CPX‐5824) complex stands out as it is exclusively detected in the *V337M* network, distinguishing it from both the tau‐F and P301L mutant networks (Table 7). This complex holds significance as an integral component of the spliceosome, playing a crucial role in the activation of pre‐mRNA splicing. Its involvement extends to participating in the rearrangement of snRNP particles, essential for the intricate process of splicing. Additionally, the PRP19‐CDC5L complex may have implications in the DNA damage response (DDR). This exclusive expression of the spliceosome complex in the mutant network raises intriguing questions about its potential contribution to the observed abnormal splicing patterns in AD.

**TABLE 7:** Summary of complexes detected either in V337M or P301L Tau‐F mutant networks.

Complexes detected only in V337M network		
Complex AC	Complex name	GO biological process
CPX‐5824	PRP19‐CDC5L complex	mRNA splicing, via spliceosome, spliceosomal snRNP assembly
CPX‐6411	tRNA‐splicing ligase complex	tRNA splicing, via endonucleolytic cleavage and ligation
CPX‐2759	CRL4‐CRBN E3 ubiquitin ligase complex, CUL4A variant	NA
CPX‐2762	CRL4‐CRBN E3 ubiquitin ligase complex, CUL4B variant	NA
CPX‐5053	Neuronal AP‐3 adaptor complex, sigma3b variant	Clathrin‐coated vesicle cargo loading, AP‐3‐mediated, intracellular transport, synaptic vesicle coating, synaptic vesicle recycling
Complexes detected only in P301L network		
CPX‐8149	PRMT5 methylosome complex, COPR5 variant	Positive regulation of rRNA processing

Recent evidence underscores the importance of splice modulators as potential therapeutic targets, especially in diseases characterised by aberrant splicing patterns. Unravelling the intricate dynamics of the PRP19‐CDC5L complex in the context of AD pathology could pave the way for innovative therapeutic interventions aimed at modulating splicing irregularities [39, 40, 41].

The V337M network reveals *tRNA‐splicing ligase complex* (CPX‐6411), which is essential for tRNA splicing and thereby regulates tRNA maturation. This complex is not enriched in either the Tau‐F network or the P301L Tau‐F network. Although a direct correlation between the tRNA‐splicing ligase complex and AD has not been established, Holzmann and Rossmanith [42] identified MRPP2/HSD17B10 (UniProtKB: Q99714), a component of mitochondrial RNase P, as a potential intracellular target in AD. Their study suggests that MRPP2 could link tRNA processing to mitochondrial dysfunction in AD, implying that disruptions in RNA processing machinery could contribute to AD pathogenesis.

The *V337M mutant* interactome reveals the exclusive presence of *CRL4‐CRBN E3 ubiquitin ligase family complexes* (CPX‐2759, CPX‐2762), signifying a distinctive regulatory profile in comparison to the Tau‐F and P301L mutant networks. This E3 ubiquitin ligase family catalyses the transfer of ubiquitin from an E2 enzyme to a substrate bound to the substrate receptor CRBN. The functional versatility of CRL4‐CRBN extends across a broad spectrum of biological processes, encompassing ion channel regulation, cancer development, immune regulation and energy metabolism regulation. Particularly noteworthy is its involvement in the ubiquitination and subsequent proteasomal degradation of a diverse array of target proteins, rendering it a crucial player in cellular homeostasis.

SIGNOR (Lo Surdo et al. [36]) shares evidence on the *CRL4‐CRBN E3 ubiquitin* (SIGNOR‐C119) complex downregulating several key regulators involved in cellular degradation and mitosis:

- MAGEA3 (UniProtKB: P43357): this protein is an activator of the ubiquitin ligase activity of RING‐type zinc finger‐containing E3 ubiquitin‐protein ligases, which act as repressors of autophagy. By downregulating MAGEA3, the Cullin4‐RBX1‐DDB1 complex can indirectly influence autophagy, potentially enhancing cellular degradation processes.
- WIPI2 (UniProtKB: Q9Y4P8): a component of the autophagy machinery, WIPI2 is crucial for the major intracellular degradation process where cytoplasmic materials are packaged into autophagosomes and delivered to lysosomes for degradation. Cullin4‐RBX1‐DDB1's downregulation of WIPI2 could negatively impact autophagosome formation and the overall autophagy process.
- CDT1 (UniProtKB: Q9H211): this protein is essential for the initiation of DNA replication and also plays a critical role in mitosis by promoting stable kinetochore‐MT attachments. Downregulation of CDT1 by Cullin4‐RBX1‐DDB1 may affect both DNA replication and mitotic progression, potentially compromising cell division and genomic stability.

These regulatory actions suggest that Cullin4‐RBX1‐DDB1 has a multifaceted role in controlling autophagy, protein degradation and cell cycle progression, which could be crucial in various cellular contexts, including cancer and neurodegenerative diseases.

Of therapeutic significance [43], CRL4‐CRBN (CHEMBL3833061, CPX‐2759/CPX‐2762) [44] is the target of thalidomide, lenalidomide and pomalidomide—clinically vital drugs employed in the treatment of multiple myeloma and other B‐cell malignancies.

Intriguingly, both mutant networks reveal the presence of the *COPII vesicle coat complex* (CPX‐2360). The assembly of this complex involves a series of intricately coordinated steps that culminate in the formation of COPII vesicles, instrumental in intracellular transport. The COPII coat's inner and outer layers adopt lattice structures, allowing flexibility in reshaping membranes. This adaptability enables the assembly of COPII vesicles with distinct sizes and shapes, facilitating the transport of a diverse range of cargoes. Chadwick et al. (2012) demonstrated that COPII vesicles are crucial for the transport of ATF6 to the Golgi compartment, where it undergoes proteolytic processing. This cleavage releases a 50 kDa leucine zipper transcription factor, which then translocates to the nucleus and binds to promoter elements of ER stress response genes. This process is an essential physiological function of the ER, and effective modulation of this pathway could potentially ameliorate or mitigate the detrimental cellular aspects associated with AD initiation and progression.

The presence of the *PRMT5 methylosome complex, COPR5 variant*, in the Tau‐F P301L mutant interactome suggests a potential regulatory mechanism in the context of AD. It is noteworthy that the Tau‐F network shows the *CLNS1A variant* of the *PRMT5 methylosome complex*, PRMT5, as a Type II arginine methyltransferase, plays a critical role in catalysing the dimethylation of substrate proteins by transferring methyl groups from S‐adenosyl methionine (SAM) cofactor molecules to arginine residues on the substrate.

The presence of the *PRMT5 methylosome complex*, specifically the *COPR5 variant* (CPX‐8149), in the Tau‐F P301L mutant interactome suggests a distinct regulatory mechanism relevant to AD. In contrast, the Tau‐F network shows the *CLNS1A variant* (CPX‐696) of the *PRMT5 methylosome complex*, which recruits the complex to subunits of the spliceosome and ribosomal proteins. This recruitment is crucial for methylating these subunits, thereby regulating cellular splicing activity and the formation of RNA processing bodies. On the other hand, the COPR5 variant directs PRMT5 to nucleosomes, promoting the methylation of arginine residues, such as H3R8 and H4R3, which is essential for chromatin remodelling [45]. This process can have significant implications for the regulation of gene expression, as histone methylation patterns influence the accessibility of DNA to transcription factors and other regulatory proteins. Moreover, the involvement of COPR5 in repressing the CCNE1 gene [45], which is essential for controlling the cell cycle at the G1/S transition [46], further underscores its regulatory role in cellular processes.

This differential recruitment highlights how specific PRMT5 complex variants might influence various cellular processes, including RNA processing and chromatin dynamics, potentially contributing to the altered molecular landscape observed in AD.

### 4.2 Amyloid Precursor Protein Network from IMEx Alzheimer's Dataset

We retrieved all interaction partners of the canonical APP protein, its isoforms, and its post‐processed chains from the dataset. This approach is informed by evidence linking APP alternative splicing to Alzheimer’s disease (Sandbrink, Masters, and Beyreuther 1996; Love, Hayden, and Rohn 2015). The APP network extracted from IMEx's AD‐specific dataset comprises over 2300 interactions (Table S5). These interactions are predominantly identified through two‐hybrid assays, resulting in more than 1900 true‐binary associations. Significantly, over 900 of these interactions are affected by mutations, underscoring their potential importance in deciphering the molecular mechanisms underlying AD.

**FIGURE 4:**
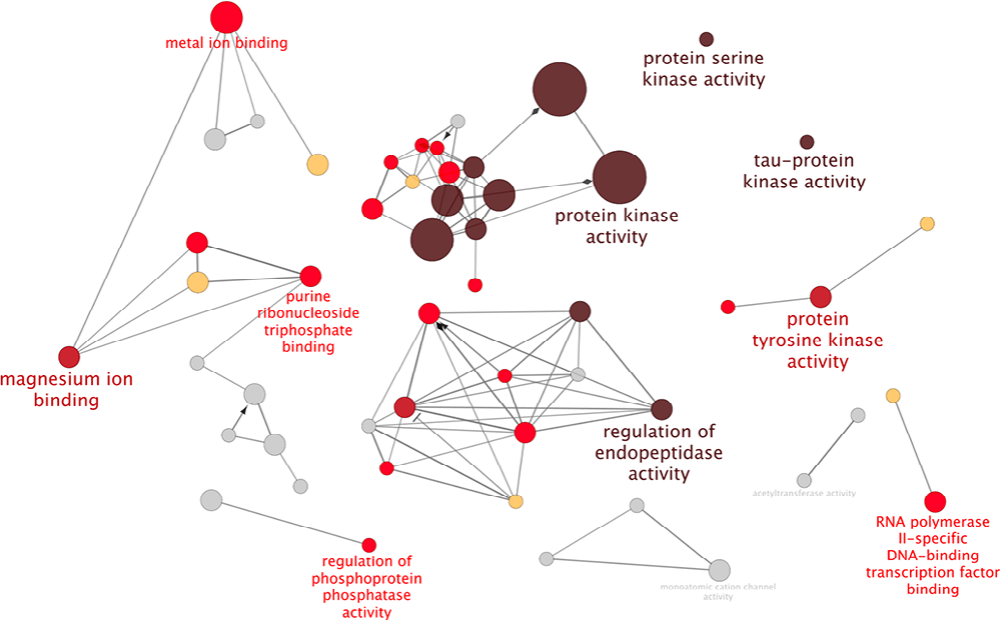
The molecular functional clusters enriched by high‐confidence amyloid precursor protein (APP) partners from the IMEx AD dataset illustrate key molecular functions associated with APP interactors.

As depicted in Figure 4, core molecular functions such as protein kinase activity, including tau protein kinase activity, stand out as particularly significant within the APP network. Other critical functions identified include the regulation of endopeptidase activity, endopeptidase inhibitor activity, as well as various binding activities like copper and magnesium ion binding, proton‐transporting activity, DNA binding and nuclear receptor binding (Table 8).

**TABLE 8:** Molecular Function clusters enriched by high‐confidence interacting partners of APP.

ID	Term	Ontology source	Term *p* value	Term *p* value corrected with Bonferroni step down	Group *p* value	Group *p* value corrected with Bonferroni step down	GOLevels	GOGroups	% Associated genes	Nr. genes	Associated genes found
GO:0050321	Tau‐protein kinase activity	GO_MolecularFunction‐EBI‐UniProt‐GOA‐ACAP‐ARAP_07.06.2024_00h00	0.00	0.00	0.00	0.00	[4, 6]	Group00	45.45	5.00	[CSNK1D, GSK3B, HSP90AA1, MARK1, MARK4]
GO:0106310	Protein serine kinase activity	GO_MolecularFunction‐EBI‐UniProt‐GOA‐ACAP‐ARAP_07.06.2024_00h00	0.00	0.00	0.00	0.00	[4, 6]	Group01	53.33	8.00	[CSNK1D, GSK3B, PIK3CB, PIK3CG, PINK1, PLK1, PRKACA, PRKCD]
GO:0016407	Acetyltransferase activity	GO_MolecularFunction‐EBI‐UniProt‐GOA‐ACAP‐ARAP_07.06.2024_00h00	0.01	0.12	0.01	0.02	[5]	Group02	8.33	6.00	[ARRB1, GTF2B, KAT5, NAA10, SAT1, TAF1]
GO:0016410	*N*‐acyltransferase activity	GO_MolecularFunction‐EBI‐UniProt‐GOA‐ACAP‐ARAP_07.06.2024_00h00	0.01	0.13	0.01	0.02	[5]	Group02	8.00	6.00	[ARRB1, KAT5, NAA10, SAT1, TAF1, TGM2]
GO:0016791	Phosphatase activity	GO_MolecularFunction‐EBI‐UniProt‐GOA‐ACAP‐ARAP_07.06.2024_00h00	0.01	0.14	0.01	0.03	[5]	Group03	5.21	11.00	[DUSP6, HSP90B1, HTT, PDGFRB, PLPP1, PPP1CA, PPP2CA, PPP2R5A, PPP3CC, ROCK2, TNF]
GO:0043666	Regulation of phosphoprotein phosphatase activity	GO_MolecularFunction‐EBI‐UniProt‐GOA‐ACAP‐ARAP_07.06.2024_00h00	0.00	0.04	0.01	0.03	[4, 5, 7]	Group03	13.89	5.00	[HSP90B1, HTT, PDGFRB, ROCK2, TNF]
GO:0061629	RNA polymerase II‐specific DNA‐binding transcription factor binding	GO_MolecularFunction‐EBI‐UniProt‐GOA‐ACAP‐ARAP_07.06.2024_00h00	0.00	0.04	0.00	0.01	[5]	Group04	5.69	16.00	[ACTB, CALR, ELK1, GSK3B, GTF2B, HMGB1, HSPB1, JUN, MBD4, PARK7, PKN1, SMAD3, TAF1, TBP, TP53BP2, ZNF366]
GO:0016922	Nuclear receptor binding	GO_MolecularFunction‐EBI‐UniProt‐GOA‐ACAP‐ARAP_07.06.2024_00h00	0.00	0.07	0.00	0.01	[6]	Group04	7.77	8.00	[CALR, GTF2B, MBD4, PARK7, PKN1, SMAD3, TAF1, ZNF366]
GO:0004713	Protein tyrosine kinase activity	GO_MolecularFunction‐EBI‐UniProt‐GOA‐ACAP‐ARAP_07.06.2024_00h00	0.00	0.00	0.00	0.00	[4, 6]	Group05	10.53	12.00	[ABL1, ADAM17, CAV1, DDR2, FYN, HYAL2, KDR, LYN, NTRK1, PDGFRB, PTK2B, SYK]
GO:0004714	Transmembrane receptor protein tyrosine kinase activity	GO_MolecularFunction‐EBI‐UniProt‐GOA‐ACAP‐ARAP_07.06.2024_00h00	0.00	0.09	0.00	0.00	[5, 7]	Group05	10.87	5.00	[ADAM17, DDR2, KDR, NTRK1, PDGFRB]
GO:0004715	Nonmembrane spanning protein tyrosine kinase activity	GO_MolecularFunction‐EBI‐UniProt‐GOA‐ACAP‐ARAP_07.06.2024_00h00	0.00	0.02	0.00	0.00	[5, 7]	Group05	17.86	5.00	[ABL1, FYN, LYN, PTK2B, SYK]
GO:0005261	Monoatomic cation channel activity	GO_MolecularFunction‐EBI‐UniProt‐GOA‐ACAP‐ARAP_07.06.2024_00h00	0.02	0.12	0.02	0.02	[5, 6]	Group06	4.74	12.00	[ATP5PO, CALHM1, CAV1, CHRNA4, CHRNA7, CLU, DRD4, GRIN1, HTT, PSEN1, TMSB4X, UBQLN1]
GO:0005262	Calcium channel activity	GO_MolecularFunction‐EBI‐UniProt‐GOA‐ACAP‐ARAP_07.06.2024_00h00	0.02	0.16	0.02	0.02	[6, 7]	Group06	6.98	6.00	[CLU, DRD4, GRIN1, HTT, PSEN1, UBQLN1]
GO:0099094	Ligand‐gated monoatomic cation channel activity	GO_MolecularFunction‐EBI‐UniProt‐GOA‐ACAP‐ARAP_07.06.2024_00h00	0.01	0.13	0.02	0.02	[6, 7, 8]	Group06	7.00	7.00	[CALHM1, CAV1, CHRNA4, CHRNA7, CLU, GRIN1, HTT]
GO:0046872	Metal ion binding	GO_MolecularFunction‐EBI‐UniProt‐GOA‐ACAP‐ARAP_07.06.2024_00h00	0.00	0.02	0.00	0.00	[5]	Group07	4.72	28.00	[ABL1, APCS, APOE, CALR, CAPN1, CLU, DDR2, GTF2B, IDE, ITGB1, MAPK12, MARK1, MME, NRG1, PARK7, PINK1, PLK1, PLSCR1, PRKACB, PRNP, S100B, SEPTIN4, SMAD3, SNCA, SNCB, SRPK2, TGM2, TK1]
GO:0000287	Magnesium ion binding	GO_MolecularFunction‐EBI‐UniProt‐GOA‐ACAP‐ARAP_07.06.2024_00h00	0.00	0.00	0.00	0.00	[6]	Group07	9.92	13.00	[ABL1, DDR2, ITGB1, MAPK12, MARK1, PINK1, PLK1, PLSCR1, PRKACB, PRNP, SEPTIN4, SNCA, SRPK2]
GO:0005509	Calcium ion binding	GO_MolecularFunction‐EBI‐UniProt‐GOA‐ACAP‐ARAP_07.06.2024_00h00	0.00	0.07	0.00	0.00	[6]	Group07	6.25	11.00	[APCS, CALR, CAPN1, CLU, DDR2, ITGB1, PLSCR1, S100B, SNCA, SNCB, TGM2]
GO:0046914	Transition metal ion binding	GO_MolecularFunction‐EBI‐UniProt‐GOA‐ACAP‐ARAP_07.06.2024_00h00	0.05	0.20	0.00	0.00	[6]	Group07	4.04	13.00	[ABL1, GTF2B, IDE, MME, NRG1, PARK7, PLSCR1, PRNP, S100B, SMAD3, SNCA, SNCB, TK1]
GO:0008270	Zinc ion binding	GO_MolecularFunction‐EBI‐UniProt‐GOA‐ACAP‐ARAP_07.06.2024_00h00	0.07	0.14	0.00	0.00	[7]	Group07	4.29	9.00	[GTF2B, IDE, MME, NRG1, PLSCR1, S100B, SMAD3, SNCA, TK1]
GO:0035639	Purine ribonucleoside triphosphate binding	GO_MolecularFunction‐EBI‐UniProt‐GOA‐ACAP‐ARAP_07.06.2024_00h00	0.00	0.02	0.00	0.00	[4, 5]	Group08	5.84	17.00	[ABL1, HSP90AA1, HSPA5, HSPA8, IDE, MARK1, PINK1, PLK1, PRKACB, PRNP, PRPS1, RAC1, RHOA, SEPTIN4, SRPK2, TGM2, TUBA1B]
GO:0032555	Purine ribonucleotide binding	GO_MolecularFunction‐EBI‐UniProt‐GOA‐ACAP‐ARAP_07.06.2024_00h00	0.00	0.05	0.00	0.00	[4, 6]	Group08	5.16	18.00	[ABL1, HSP90AA1, HSPA5, HSPA8, IDE, MARK1, PINK1, PLK1, PRKACB, PRNP, PRPS1, RAC1, RHOA, SEPTIN4, SRPK2, TAF1, TGM2, TUBA1B]
GO:0046872	Metal ion binding	GO_MolecularFunction‐EBI‐UniProt‐GOA‐ACAP‐ARAP_07.06.2024_00h00	0.00	0.02	0.00	0.00	[5]	Group08	4.72	28.00	[ABL1, APCS, APOE, CALR, CAPN1, CLU, DDR2, GTF2B, IDE, ITGB1, MAPK12, MARK1, MME, NRG1, PARK7, PINK1, PLK1, PLSCR1, PRKACB, PRNP, S100B, SEPTIN4, SMAD3, SNCA, SNCB, SRPK2, TGM2, TK1]
GO:0000287	Magnesium ion binding	GO_MolecularFunction‐EBI‐UniProt‐GOA‐ACAP‐ARAP_07.06.2024_00h00	0.00	0.00	0.00	0.00	[6]	Group08	9.92	13.00	[ABL1, DDR2, ITGB1, MAPK12, MARK1, PINK1, PLK1, PLSCR1, PRKACB, PRNP, SEPTIN4, SNCA, SRPK2]
GO:0032559	Adenyl ribonucleotide binding	GO_MolecularFunction‐EBI‐UniProt‐GOA‐ACAP‐ARAP_07.06.2024_00h00	0.00	0.09	0.00	0.00	[5, 7]	Group08	5.56	13.00	[ABL1, HSP90AA1, HSPA5, HSPA8, IDE, MARK1, PINK1, PLK1, PRKACB, PRNP, PRPS1, SRPK2, TAF1]
GO:0005525	GTP binding	GO_MolecularFunction‐EBI‐UniProt‐GOA‐ACAP‐ARAP_07.06.2024_00h00	0.10	0.10	0.00	0.00	[5, 6, 8]	Group08	4.90	5.00	[RAC1, RHOA, SEPTIN4, TGM2, TUBA1B]
GO:0016887	ATP hydrolysis activity	GO_MolecularFunction‐EBI‐UniProt‐GOA‐ACAP‐ARAP_07.06.2024_00h00	0.05	0.23	0.00	0.00	[2, 7]	Group09	6.17	5.00	[ACTB, CLU, HSP90AA1, HSPA5, TOR1A]
GO:0035639	Purine ribonucleoside triphosphate binding	GO_MolecularFunction‐EBI‐UniProt‐GOA‐ACAP‐ARAP_07.06.2024_00h00	0.00	0.02	0.00	0.00	[4, 5]	Group09	5.84	17.00	[ABL1, HSP90AA1, HSPA5, HSPA8, IDE, MARK1, PINK1, PLK1, PRKACB, PRNP, PRPS1, RAC1, RHOA, SEPTIN4, SRPK2, TGM2, TUBA1B]
GO:0017111	Ribonucleoside triphosphate phosphatase activity	GO_MolecularFunction‐EBI‐UniProt‐GOA‐ACAP‐ARAP_07.06.2024_00h00	0.01	0.12	0.00	0.00	[6]	Group09	4.65	16.00	[ACTB, ADCYAP1, CLU, DVL3, GSK3B, HSP90AA1, HSPA5, ITGB1, NF1, NTRK1, PIN1, RAC1, RHOA, SEPTIN4, TOR1A, TUBA1B]
GO:0005525	GTP binding	GO_MolecularFunction‐EBI‐UniProt‐GOA‐ACAP‐ARAP_07.06.2024_00h00	0.10	0.10	0.00	0.00	[5, 6, 8]	Group09	4.90	5.00	[RAC1, RHOA, SEPTIN4, TGM2, TUBA1B]
GO:0043547	Positive regulation of GTPase activity	GO_MolecularFunction‐EBI‐UniProt‐GOA‐ACAP‐ARAP_07.06.2024_00h00	0.02	0.13	0.00	0.00	[4, 5, 8, 9]	Group09	6.31	7.00	[ADCYAP1, DVL3, GSK3B, ITGB1, NF1, NTRK1, PIN1]
GO:0003924	GTPase activity	GO_MolecularFunction‐EBI‐UniProt‐GOA‐ACAP‐ARAP_07.06.2024_00h00	0.05	0.16	0.00	0.00	[7]	Group09	4.21	11.00	[ADCYAP1, DVL3, GSK3B, ITGB1, NF1, NTRK1, PIN1, RAC1, RHOA, SEPTIN4, TUBA1B]
GO:0010466	Negative regulation of peptidase activity	GO_MolecularFunction‐EBI‐UniProt‐GOA‐ACAP‐ARAP_07.06.2024_00h00	0.00	0.03	0.00	0.00	[4, 5]	Group10	9.30	8.00	[CSNK2A1, CST3, ECM1, GAPDH, PARK7, SNCA, THBS1, TNF]
GO:0004197	Cysteine‐type endopeptidase activity	GO_MolecularFunction‐EBI‐UniProt‐GOA‐ACAP‐ARAP_07.06.2024_00h00	0.00	0.00	0.00	0.00	[5]	Group10	7.32	15.00	[BAD, CAPN1, CASP3, CASP8, CSNK2A1, HMGB1, HSPD1, NGFR, PARK7, SMAD3, SNCA, SYK, THBS1, TNF, UCHL1]
GO:0052548	Regulation of endopeptidase activity	GO_MolecularFunction‐EBI‐UniProt‐GOA‐ACAP‐ARAP_07.06.2024_00h00	0.00	0.00	0.00	0.00	[5]	Group10	9.45	19.00	[AGER, APH1A, APH1B, BAD, CASP8, CSNK2A1, FURIN, GAPDH, HMGB1, HSPD1, LYN, NGFR, PARK7, PSENEN, SMAD3, SNCA, SYK, THBS1, TNF]
GO:0010950	Positive regulation of endopeptidase activity	GO_MolecularFunction‐EBI‐UniProt‐GOA‐ACAP‐ARAP_07.06.2024_00h00	0.00	0.00	0.00	0.00	[5, 6]	Group10	11.48	14.00	[AGER, APH1A, APH1B, BAD, CASP8, HMGB1, HSPD1, LYN, NGFR, PSENEN, SMAD3, SNCA, SYK, TNF]
GO:0010951	Negative regulation of endopeptidase activity	GO_MolecularFunction‐EBI‐UniProt‐GOA‐ACAP‐ARAP_07.06.2024_00h00	0.01	0.12	0.00	0.00	[5, 6]	Group10	7.79	6.00	[CSNK2A1, GAPDH, PARK7, SNCA, THBS1, TNF]
GO:0097153	Cysteine‐type endopeptidase activity involved in apoptotic process	GO_MolecularFunction‐EBI‐UniProt‐GOA‐ACAP‐ARAP_07.06.2024_00h00	0.00	0.00	0.00	0.00	[6]	Group10	9.09	13.00	[BAD, CASP3, CASP8, CSNK2A1, HMGB1, HSPD1, NGFR, PARK7, SMAD3, SNCA, SYK, THBS1, TNF]
GO:2000116	Regulation of cysteine‐type endopeptidase activity	GO_MolecularFunction‐EBI‐UniProt‐GOA‐ACAP‐ARAP_07.06.2024_00h00	0.00	0.02	0.00	0.00	[6]	Group10	7.55	12.00	[BAD, CASP8, CSNK2A1, HMGB1, HSPD1, NGFR, PARK7, SMAD3, SNCA, SYK, THBS1, TNF]
GO:2001056	Positive regulation of cysteine‐type endopeptidase activity	GO_MolecularFunction‐EBI‐UniProt‐GOA‐ACAP‐ARAP_07.06.2024_00h00	0.00	0.03	0.00	0.00	[6, 7]	Group10	8.74	9.00	[BAD, CASP8, HMGB1, HSPD1, NGFR, SMAD3, SNCA, SYK, TNF]
GO:0043154	Negative regulation of cysteine‐type endopeptidase activity involved in apoptotic process	GO_MolecularFunction‐EBI‐UniProt‐GOA‐ACAP‐ARAP_07.06.2024_00h00	0.01	0.10	0.00	0.00	[7, 8]	Group10	10.42	5.00	[CSNK2A1, PARK7, SNCA, THBS1, TNF]
GO:0006919	Activation of cysteine‐type endopeptidase activity involved in apoptotic process	GO_MolecularFunction‐EBI‐UniProt‐GOA‐ACAP‐ARAP_07.06.2024_00h00	0.01	0.10	0.00	0.00	[8, 9]	Group10	10.20	5.00	[HSPD1, NGFR, SMAD3, SNCA, TNF]
GO:0004672	Protein kinase activity	GO_MolecularFunction‐EBI‐UniProt‐GOA‐ACAP‐ARAP_07.06.2024_00h00	0.00	0.00	0.00	0.00	[3, 5]	Group11	8.70	57.00	[ABL1, ADAM17, ADCYAP1, APOE, CAMK2A, CAV1, CDK1, CDK5, CDKN2C, CSNK1D, CSNK2A1, DDR2, DRD4, FYN, GRK2, GRK6, GSK3A, GSK3B, HSP90AA1, HTT, HYAL2, KDR, LYN, MAP2K2, MAPK1, MAPK12, MARK1, MARK4, NRG1, NTRK1, PARK7, PDGFRB, PDK1, PIK3CB, PIK3CG, PINK1, PKN1, PLK1, PPIA, PPP2CA, PRKACA, PRKACB, PRKCD, PRKCE, PTK2B, RAF1, RHOA, ROCK2, RPS6KA2, SNCA, SRPK2, SYK, TAF1, TGFB2, THBS1, TNF, UCHL1]
GO:0043549	Regulation of kinase activity	GO_MolecularFunction‐EBI‐UniProt‐GOA‐ACAP‐ARAP_07.06.2024_00h00	0.00	0.00	0.00	0.00	[4, 5]	Group11	9.09	31.00	[ABL1, ADAM17, ADCYAP1, APOE, CAV1, CDKN2C, DDR2, DRD4, F2, HSP90AA1, HTT, HYAL2, MAP2K2, MAPT, NRG1, PARK7, PIK3CG, PKN1, PLK1, PPIA, PPP2CA, PPP2R5A, PRKCD, PTK2B, RHOA, SNCA, TGFB2, THBS1, TNF, TREM2, UCHL1]
GO:0004674	Protein serine/threonine kinase activity	GO_MolecularFunction‐EBI‐UniProt‐GOA‐ACAP‐ARAP_07.06.2024_00h00	0.00	0.00	0.00	0.00	[4, 6]	Group11	9.46	40.00	[ABL1, ADAM17, APOE, CAMK2A, CDK1, CDK5, CDKN2C, CSNK1D, CSNK2A1, DRD4, FYN, GRK2, GSK3A, GSK3B, HTT, HYAL2, MAP2K2, MAPK1, MAPK12, MARK1, MARK4, PIK3CG, PINK1, PKN1, PLK1, PPP2CA, PRKACA, PRKACB, PRKCD, PRKCE, RAF1, RHOA, ROCK2, SNCA, SRPK2, SYK, TAF1, THBS1, TNF, UCHL1]
GO:0033673	Negative regulation of kinase activity	GO_MolecularFunction‐EBI‐UniProt‐GOA‐ACAP‐ARAP_07.06.2024_00h00	0.00	0.01	0.00	0.00	[4, 5, 6]	Group11	9.02	11.00	[ABL1, APOE, CAV1, HYAL2, MAPT, PARK7, PLK1, PPIA, PPP2R5A, PRKCD, UCHL1]
GO:0033674	Positive regulation of kinase activity	GO_MolecularFunction‐EBI‐UniProt‐GOA‐ACAP‐ARAP_07.06.2024_00h00	0.00	0.00	0.00	0.00	[4, 5, 6]	Group11	9.91	21.00	[ABL1, ADAM17, ADCYAP1, DDR2, DRD4, F2, HSP90AA1, MAP2K2, NRG1, PIK3CG, PKN1, PPIA, PPP2CA, PRKCD, PTK2B, RHOA, SNCA, TGFB2, THBS1, TNF, TREM2]
GO:0045859	Regulation of protein kinase activity	GO_MolecularFunction‐EBI‐UniProt‐GOA‐ACAP‐ARAP_07.06.2024_00h00	0.00	0.00	0.00	0.00	[4, 5, 6]	Group11	8.63	27.00	[ABL1, ADAM17, ADCYAP1, APOE, CAV1, CDKN2C, DDR2, DRD4, HSP90AA1, HTT, HYAL2, MAP2K2, NRG1, PARK7, PIK3CG, PKN1, PLK1, PPIA, PPP2CA, PRKCD, PTK2B, RHOA, SNCA, TGFB2, THBS1, TNF, UCHL1]
GO:0006469	Negative regulation of protein kinase activity	GO_MolecularFunction‐EBI‐UniProt‐GOA‐ACAP‐ARAP_07.06.2024_00h00	0.00	0.04	0.00	0.00	[4, 5, 6, 7]	Group11	7.96	9.00	[ABL1, APOE, CAV1, HYAL2, PARK7, PLK1, PPIA, PRKCD, UCHL1]
GO:0045860	Positive regulation of protein kinase activity	GO_MolecularFunction‐EBI‐UniProt‐GOA‐ACAP‐ARAP_07.06.2024_00h00	0.00	0.00	0.00	0.00	[4, 5, 6, 7]	Group11	9.64	19.00	[ABL1, ADAM17, ADCYAP1, DDR2, DRD4, HSP90AA1, MAP2K2, NRG1, PIK3CG, PKN1, PPIA, PPP2CA, PRKCD, PTK2B, RHOA, SNCA, TGFB2, THBS1, TNF]
GO:0004707	MAP kinase activity	GO_MolecularFunction‐EBI‐UniProt‐GOA‐ACAP‐ARAP_07.06.2024_00h00	0.00	0.04	0.00	0.00	[5, 7]	Group11	8.18	9.00	[APOE, DRD4, HYAL2, PIK3CG, PKN1, PRKCD, THBS1, TNF, UCHL1]
GO:0071900	Regulation of protein serine/threonine kinase activity	GO_MolecularFunction‐EBI‐UniProt‐GOA‐ACAP‐ARAP_07.06.2024_00h00	0.00	0.00	0.00	0.00	[5, 6, 7]	Group11	10.23	18.00	[ABL1, ADAM17, APOE, CDKN2C, DRD4, HTT, HYAL2, MAP2K2, PIK3CG, PKN1, PLK1, PPP2CA, PRKCD, RHOA, SNCA, THBS1, TNF, UCHL1]
GO:0032147	Activation of protein kinase activity	GO_MolecularFunction‐EBI‐UniProt‐GOA‐ACAP‐ARAP_07.06.2024_00h00	0.00	0.04	0.00	0.00	[5, 6, 7, 8]	Group11	11.54	6.00	[ABL1, NRG1, PPIA, PRKCD, PTK2B, TGFB2]
GO:0071901	Negative regulation of protein serine/threonine kinase activity	GO_MolecularFunction‐EBI‐UniProt‐GOA‐ACAP‐ARAP_07.06.2024_00h00	0.00	0.08	0.00	0.00	[5, 6, 7, 8]	Group11	9.84	6.00	[ABL1, APOE, HYAL2, PLK1, PRKCD, UCHL1]
GO:0071902	Positive regulation of protein serine/threonine kinase activity	GO_MolecularFunction‐EBI‐UniProt‐GOA‐ACAP‐ARAP_07.06.2024_00h00	0.00	0.01	0.00	0.00	[5, 6, 7, 8]	Group11	10.10	10.00	[ADAM17, DRD4, MAP2K2, PIK3CG, PKN1, PPP2CA, RHOA, SNCA, THBS1, TNF]
GO:0043405	Regulation of MAP kinase activity	GO_MolecularFunction‐EBI‐UniProt‐GOA‐ACAP‐ARAP_07.06.2024_00h00	0.00	0.03	0.00	0.00	[6, 7, 8]	Group11	8.74	9.00	[APOE, DRD4, HYAL2, PIK3CG, PKN1, PRKCD, THBS1, TNF, UCHL1]
GO:0043406	Positive regulation of MAP kinase activity	GO_MolecularFunction‐EBI‐UniProt‐GOA‐ACAP‐ARAP_07.06.2024_00h00	0.02	0.14	0.00	0.00	[6, 7, 8, 9]	Group11	7.94	5.00	[DRD4, PIK3CG, PKN1, THBS1, TNF]

#### 4.2.1 APP Mutations Affecting Interactions

**FIGURE 5:**
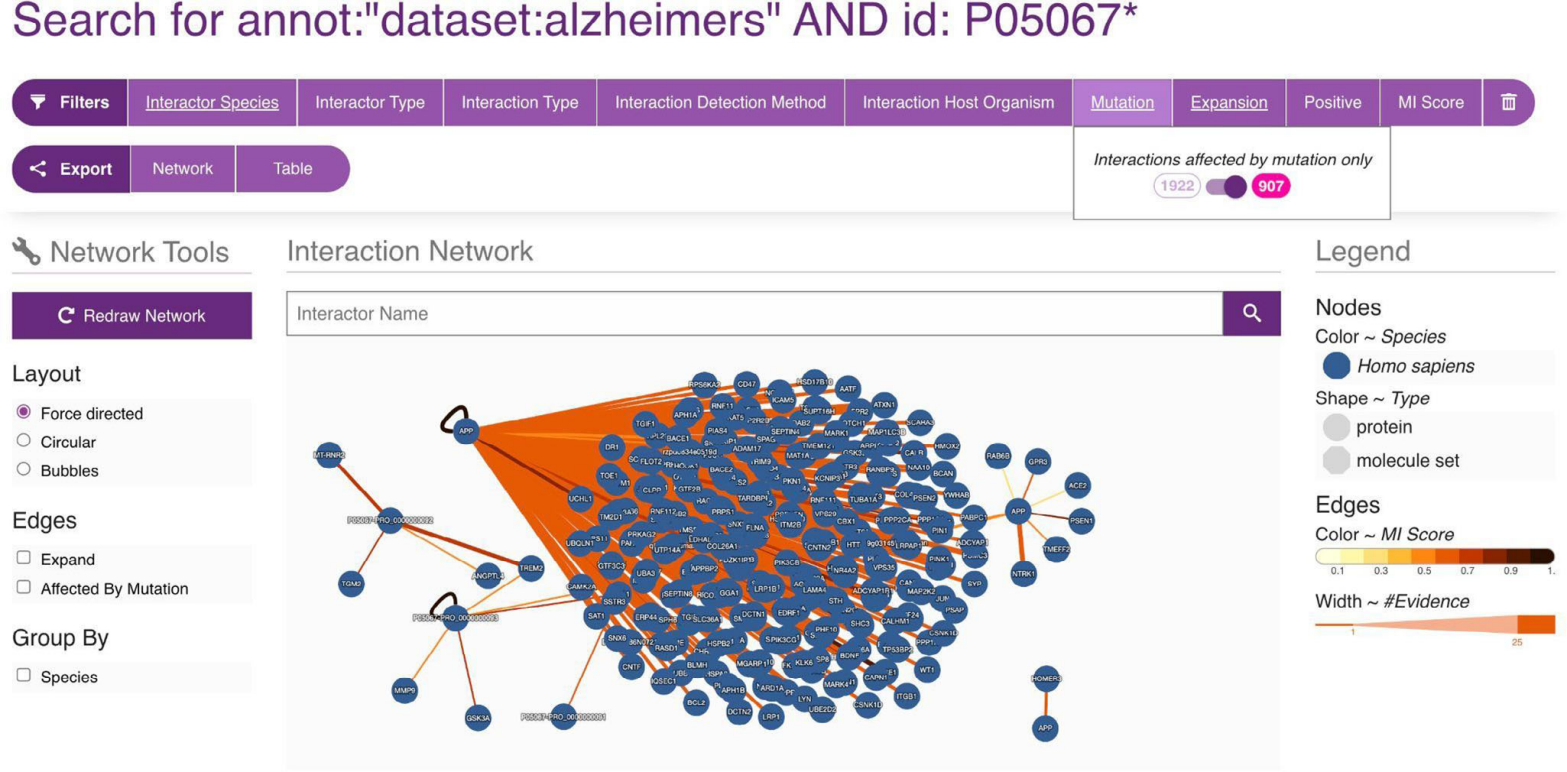
Human amyloid precursor protein (APP) physical interactions affected by mutation, selected by advanced filter options. This network illustrates the altered connections between APP and its binding partners, emphasising the significant changes in physical associations.

We further expanded our analysis to the APP mutation network to examine how genetic variations could disrupt APP's functional interactions, potentially revealing novel targets for therapeutic intervention. Given that most interactions are categorised as ‘physical associations,’ we excluded spoke‐expanded binaries to simplify the interpretation (Table S6). Figure 5 displays the interaction network affected by the APP mutation.

Remarkably, among all the physical associations affected by APP mutations, close to 880 interactions are specifically impacted by the APP Swedish mutation, characterised by the amino acid change at Lys‐670 and Met‐671 [*P05067:p.Lys670_Met671delinsAsnLeu* (rs281865161)]. This mutation is known to increase the production of Aβ peptides, a key factor in AD pathology, and the significant number of interactions affected by this mutation underscores its potential influence on APP's role in various cellular processes.

**FIGURE 6:**
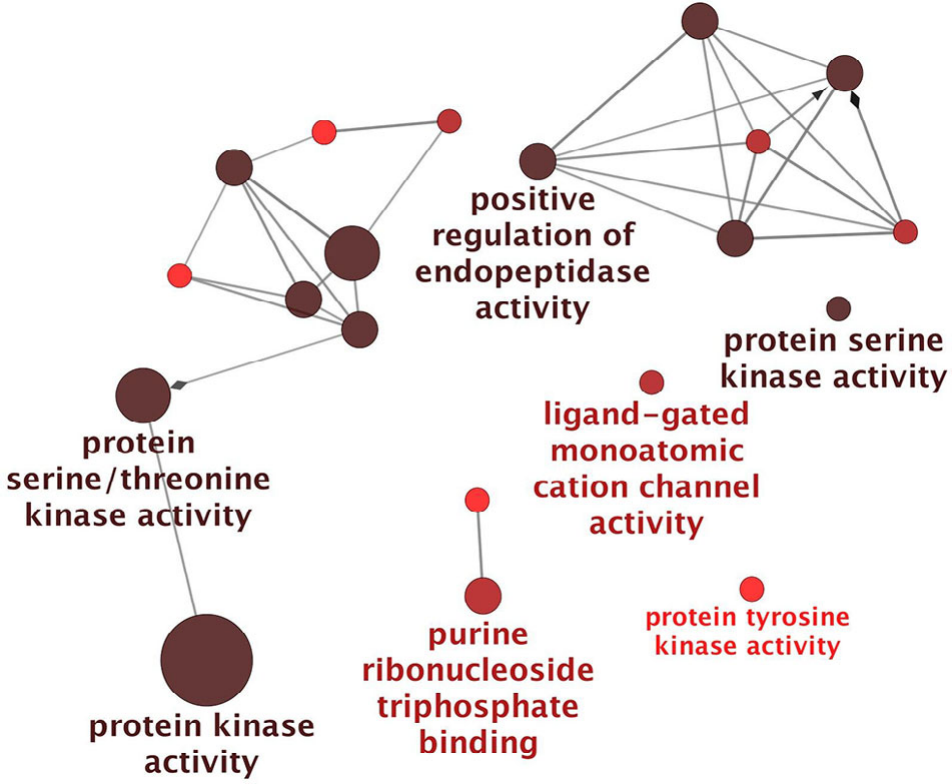
Enrichment analysis for Gene Ontology (GO) term Molecular Function using ClueGO was for amyloid precursor protein (APP) partners that are critically affected due to the Swedish mutation *P05067:p.Lys670_Met671delinsAsnLeu*.

**FIGURE 7:**
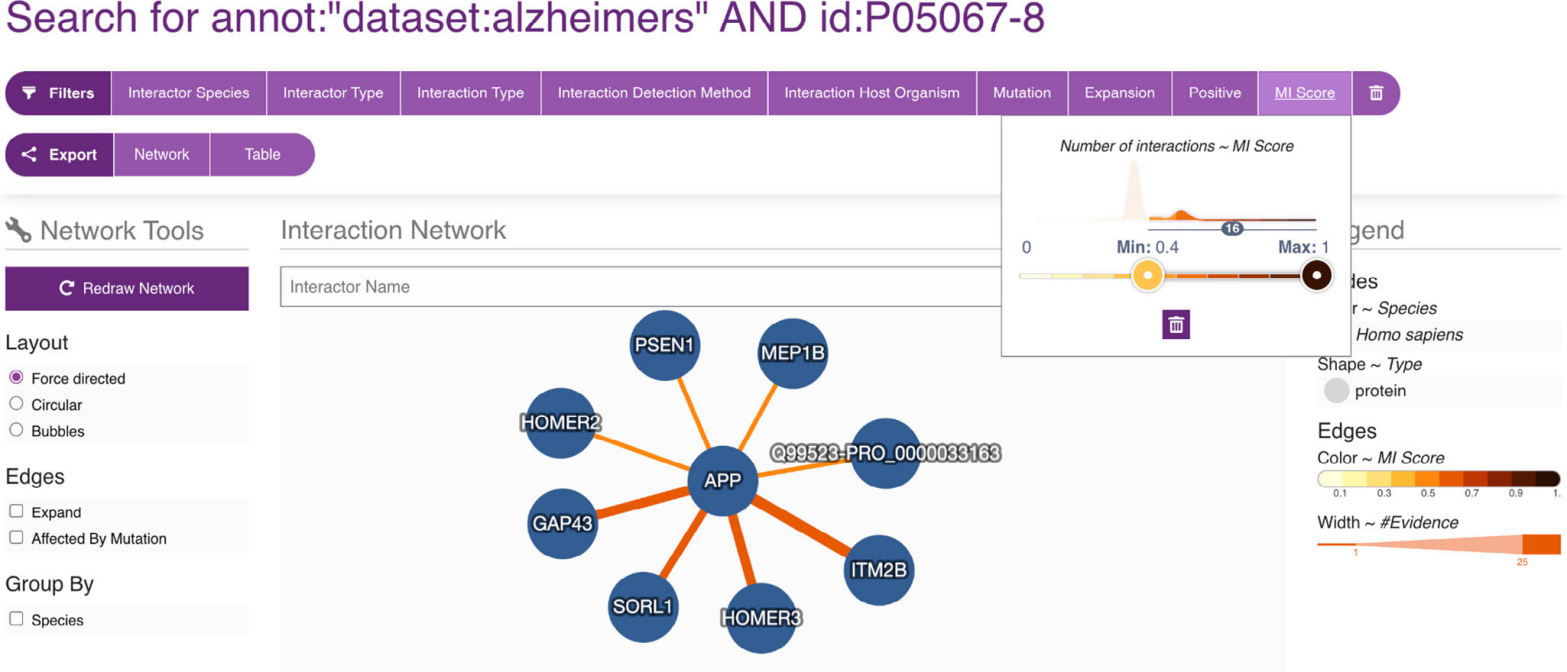
Amyloid precursor protein (APP)751 interactome of mid‐confidence, molecular interaction (MI) score of 0.4 and above generated using Molecular Interaction Query Language (MIQL) query.

**FIGURE 8:**
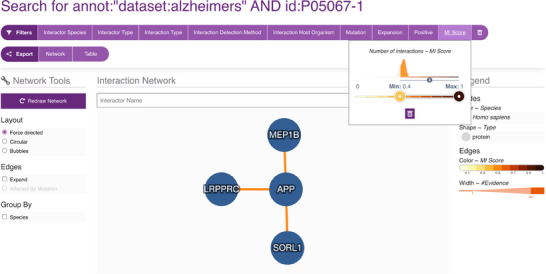
Amyloid precursor protein (APP)770 interactome of mid‐confidence score of 0.4 and above generated using Molecular Interaction Query Language (MIQL) query.

**FIGURE 9:**
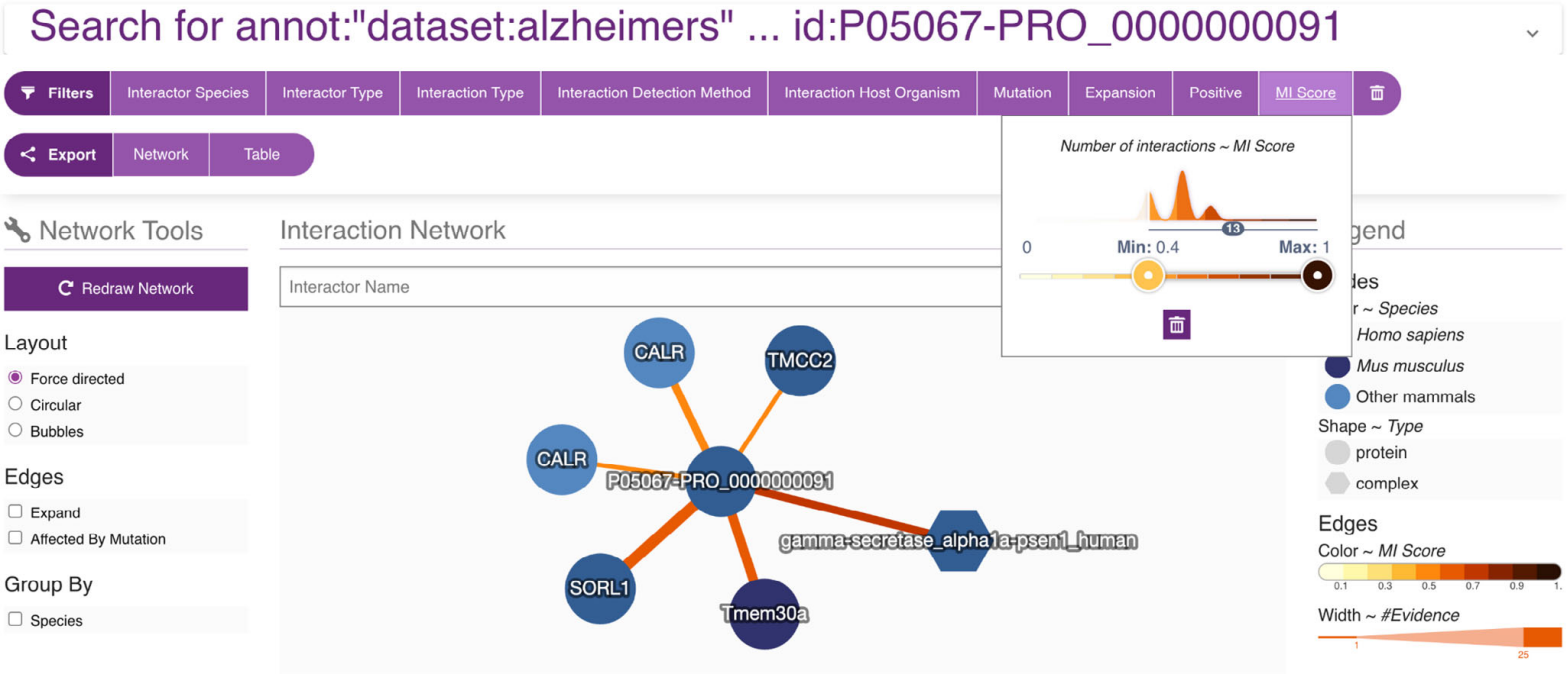
C99 interactome of mid‐confidence score of 0.4 and above using Molecular Interaction Query Language (MIQL) query.

**FIGURE 10:**
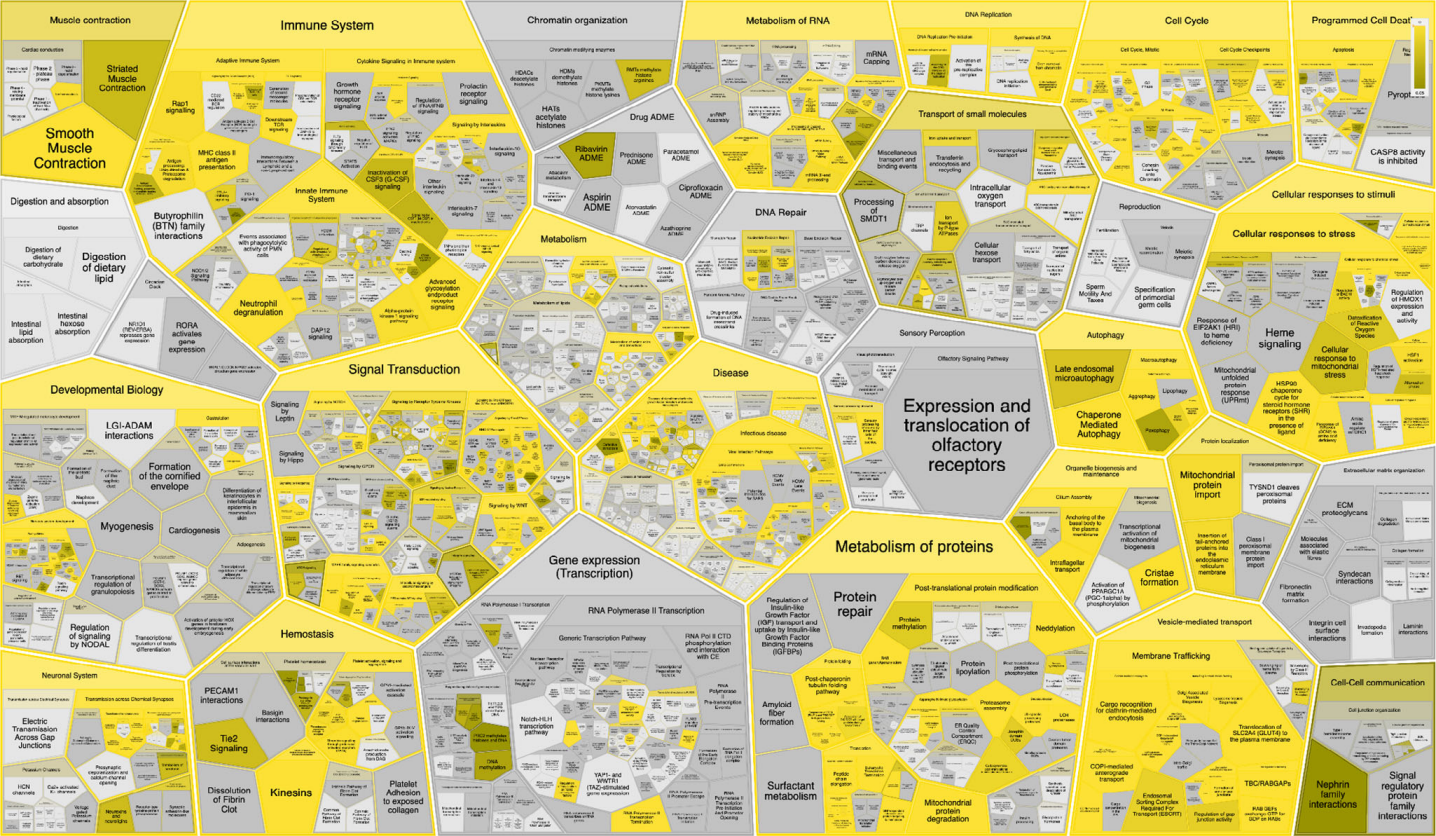
Reacfoam visualisation of all human pathways enriched for Tau‐F partners. The scale in the top right corner represents the *p* values from the over‐representation analysis for the molecules selected in each pathway. The top of the scale, shown in yellow, corresponds to *p* values close to zero, while the bottom, in greyish yellow, represents the 0.05 significance threshold.

**FIGURE 11:**
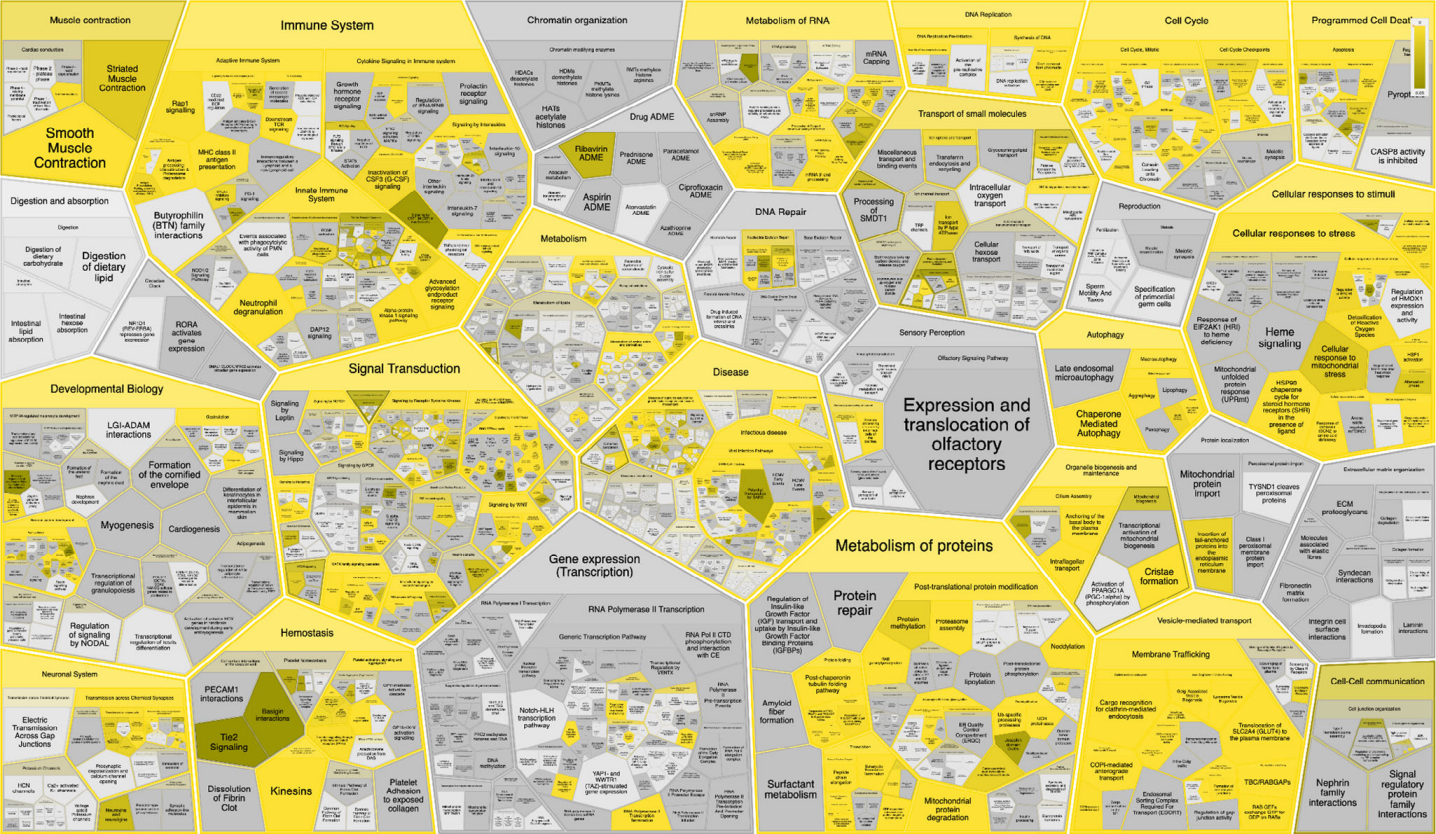
Reacfoam visualisation of all human pathways enriched for V337M Tau‐F partners.

**FIGURE 12:**
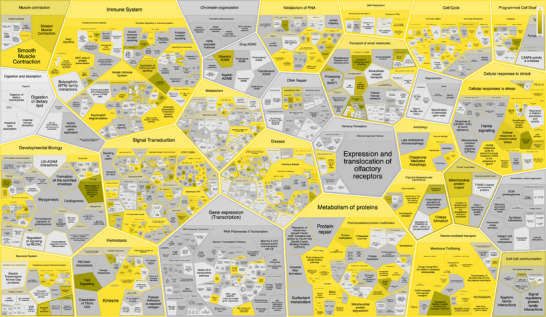
Reacfoam visualisation of all human pathways enriched for P301L mutant Tau‐F.

There are over 280 unique partners of APP whose interactions are affected by the APP Swedish mutation. Of these, nearly 190 partners experience a significant loss of interaction with APP due to this mutation. GO Molecular Functional enrichment analysis of these partners shows the critical functions impacted due to the APP mutation as depicted in Figure 6. Table 9 lists the results of the Molecular Function enrichment analysis of the partners affected by the APP mutation.

**TABLE 9:** Molecular function clusters enriched by high‐confidence interactors of Swedish mutant APP.

ID	Term	Ontology source	Term *p* value	Term *p* value corrected with Bonferroni step down	Group *p* value	Group *p* value corrected with Bonferroni step down	GOLevels	GOGroups	% Associated genes	Nr. genes	Associated genes found
GO:0099094	Ligand‐gated monoatomic cation channel activity	GO_MolecularFunction‐EBI‐UniProt‐GOA‐ACAP‐ARAP_07.06.2024_00h00	0.00	0.00	0.00	0.00	[6, 7, 8]	Group0	7.00	7.00	[CALHM1, CAV1, CHRNA4, CHRNA7, CLU, GRIN1, HTT]
GO:0106310	Protein serine kinase activity	GO_MolecularFunction‐EBI‐UniProt‐GOA‐ACAP‐ARAP_07.06.2024_00h00	0.00	0.00	0.00	0.00	[4, 6]	Group1	46.67	7.00	[CSNK1D, PIK3CB, PIK3CG, PINK1, PLK1, PRKACA, PRKCD]
GO:0004713	Protein tyrosine kinase activity	GO_MolecularFunction‐EBI‐UniProt‐GOA‐ACAP‐ARAP_07.06.2024_00h00	0.01	0.01	0.01	0.01	[4, 6]	Group2	5.26	6.00	[CAV1, FYN, LYN, PDGFRB, PTK2B, SYK]
GO:0035639	Purine ribonucleoside triphosphate binding	GO_MolecularFunction‐EBI‐UniProt‐GOA‐ACAP‐ARAP_07.06.2024_00h00	0.00	0.00	0.00	0.00	[4, 5]	Group3	4.47	13.00	[HSP90AA1, HSPA5, HSPA8, IDE, MARK1, PINK1, PLK1, PRKACB, PRPS1, RAC1, RHOA, SEPTIN4, TUBA1B]
GO:0005524	ATP binding	GO_MolecularFunction‐EBI‐UniProt‐GOA‐ACAP‐ARAP_07.06.2024_00h00	0.00	0.01	0.00	0.00	[5, 6, 8]	Group3	4.59	9.00	[HSP90AA1, HSPA5, HSPA8, IDE, MARK1, PINK1, PLK1, PRKACB, PRPS1]
GO:0004672	Protein kinase activity	GO_MolecularFunction‐EBI‐UniProt‐GOA‐ACAP‐ARAP_07.06.2024_00h00	0.00	0.00	0.00	0.00	[3, 5]	Group4	6.11	40.00	[ADCYAP1, CAMK2A, CAV1, CDK1, CDK5, CDKN2C, CSNK1D, CSNK2A1, FYN, GRK2, GRK6, HSP90AA1, HTT, LYN, MAP2K2, MAPK1, MAPK12, MARK1, MARK4, NRG1, PARK7, PDGFRB, PDK1, PIK3CB, PIK3CG, PINK1, PKN1, PLK1, PPIA, PPP2CA, PRKACA, PRKACB, PRKCD, PRKCE, PTK2B, RHOA, RPS6KA2, SNCA, SYK, UCHL1]
GO:0004674	Protein serine/threonine kinase activity	GO_MolecularFunction‐EBI‐UniProt‐GOA‐ACAP‐ARAP_07.06.2024_00h00	0.00	0.00	0.00	0.00	[4, 6]	Group4	6.38	27.00	[CAMK2A, CDK1, CDK5, CDKN2C, CSNK1D, CSNK2A1, FYN, GRK2, HTT, MAP2K2, MAPK1, MAPK12, MARK1, MARK4, PIK3CG, PINK1, PKN1, PLK1, PPP2CA, PRKACA, PRKACB, PRKCD, PRKCE, RHOA, SNCA, SYK, UCHL1]
GO:0071900	Regulation of protein serine/threonine kinase activity	GO_MolecularFunction‐EBI‐UniProt‐GOA‐ACAP‐ARAP_07.06.2024_00h00	0.00	0.00	0.00	0.00	[5, 6, 7]	Group4	6.25	11.00	[CDKN2C, HTT, MAP2K2, PIK3CG, PKN1, PLK1, PPP2CA, PRKCD, RHOA, SNCA, UCHL1]
GO:0004197	Cysteine‐type endopeptidase activity	GO_MolecularFunction‐EBI‐UniProt‐GOA‐ACAP‐ARAP_07.06.2024_00h00	0.00	0.00	0.00	0.00	[5]	Group5	5.85	12.00	[BAD, CAPN1, CASP3, CASP8, CSNK2A1, HMGB1, HSPD1, PARK7, SMAD3, SNCA, SYK, UCHL1]
GO:0052548	Regulation of endopeptidase activity	GO_MolecularFunction‐EBI‐UniProt‐GOA‐ACAP‐ARAP_07.06.2024_00h00	0.00	0.00	0.00	0.00	[5]	Group5	7.46	15.00	[AGER, APH1A, APH1B, BAD, CASP8, CSNK2A1, FURIN, HMGB1, HSPD1, LYN, PARK7, PSENEN, SMAD3, SNCA, SYK]
GO:0010950	Positive regulation of endopeptidase activity	GO_MolecularFunction‐EBI‐UniProt‐GOA‐ACAP‐ARAP_07.06.2024_00h00	0.00	0.00	0.00	0.00	[5, 6]	Group5	9.84	12.00	[AGER, APH1A, APH1B, BAD, CASP8, HMGB1, HSPD1, LYN, PSENEN, SMAD3, SNCA, SYK]
GO:0097153	Cysteine‐type endopeptidase activity involved in apoptotic process	GO_MolecularFunction‐EBI‐UniProt‐GOA‐ACAP‐ARAP_07.06.2024_00h00	0.00	0.00	0.00	0.00	[6]	Group5	6.99	10.00	[BAD, CASP3, CASP8, CSNK2A1, HMGB1, HSPD1, PARK7, SMAD3, SNCA, SYK]
GO:2000116	Regulation of cysteine‐type endopeptidase activity	GO_MolecularFunction‐EBI‐UniProt‐GOA‐ACAP‐ARAP_07.06.2024_00h00	0.00	0.00	0.00	0.00	[6]	Group5	5.66	9.00	[BAD, CASP8, CSNK2A1, HMGB1, HSPD1, PARK7, SMAD3, SNCA, SYK]
GO:2001056	Positive regulation of cysteine‐type endopeptidase activity	GO_MolecularFunction‐EBI‐UniProt‐GOA‐ACAP‐ARAP_07.06.2024_00h00	0.00	0.00	0.00	0.00	[6, 7]	Group5	6.80	7.00	[BAD, CASP8, HMGB1, HSPD1, SMAD3, SNCA, SYK]
GO:0043549	Regulation of kinase activity	GO_MolecularFunction‐EBI‐UniProt‐GOA‐ACAP‐ARAP_07.06.2024_00h00	0.00	0.00	0.00	0.00	[4, 5]	Group6	5.87	20.00	[ADCYAP1, CAV1, CDKN2C, HSP90AA1, HTT, MAP2K2, MAPT, NRG1, PARK7, PIK3CG, PKN1, PLK1, PPIA, PPP2CA, PPP2R5A, PRKCD, PTK2B, RHOA, SNCA, UCHL1]
GO:0004674	Protein serine/threonine kinase activity	GO_MolecularFunction‐EBI‐UniProt‐GOA‐ACAP‐ARAP_07.06.2024_00h00	0.00	0.00	0.00	0.00	[4, 6]	Group6	6.38	27.00	[CAMK2A, CDK1, CDK5, CDKN2C, CSNK1D, CSNK2A1, FYN, GRK2, HTT, MAP2K2, MAPK1, MAPK12, MARK1, MARK4, PIK3CG, PINK1, PKN1, PLK1, PPP2CA, PRKACA, PRKACB, PRKCD, PRKCE, RHOA, SNCA, SYK, UCHL1]
GO:0033673	Negative regulation of kinase activity	GO_MolecularFunction‐EBI‐UniProt‐GOA‐ACAP‐ARAP_07.06.2024_00h00	0.00	0.00	0.00	0.00	[4, 5, 6]	Group6	6.56	8.00	[CAV1, MAPT, PARK7, PLK1, PPIA, PPP2R5A, PRKCD, UCHL1]
GO:0045859	Regulation of protein kinase activity	GO_MolecularFunction‐EBI‐UniProt‐GOA‐ACAP‐ARAP_07.06.2024_00h00	0.00	0.00	0.00	0.00	[4, 5, 6]	Group6	5.75	18.00	[ADCYAP1, CAV1, CDKN2C, HSP90AA1, HTT, MAP2K2, NRG1, PARK7, PIK3CG, PKN1, PLK1, PPIA, PPP2CA, PRKCD, PTK2B, RHOA, SNCA, UCHL1]
GO:0006469	Negative regulation of protein kinase activity	GO_MolecularFunction‐EBI‐UniProt‐GOA‐ACAP‐ARAP_07.06.2024_00h00	0.01	0.01	0.00	0.00	[4, 5, 6, 7]	Group6	5.31	6.00	[CAV1, PARK7, PLK1, PPIA, PRKCD, UCHL1]
GO:0045860	Positive regulation of protein kinase activity	GO_MolecularFunction‐EBI‐UniProt‐GOA‐ACAP‐ARAP_07.06.2024_00h00	0.00	0.00	0.00	0.00	[4, 5, 6, 7]	Group6	6.09	12.00	[ADCYAP1, HSP90AA1, MAP2K2, NRG1, PIK3CG, PKN1, PPIA, PPP2CA, PRKCD, PTK2B, RHOA, SNCA]
GO:0071900	Regulation of protein serine/threonine kinase activity	GO_MolecularFunction‐EBI‐UniProt‐GOA‐ACAP‐ARAP_07.06.2024_00h00	0.00	0.00	0.00	0.00	[5, 6, 7]	Group6	6.25	11.00	[CDKN2C, HTT, MAP2K2, PIK3CG, PKN1, PLK1, PPP2CA, PRKCD, RHOA, SNCA, UCHL1]
GO:0071902	Positive regulation of protein serine/threonine kinase activity	GO_MolecularFunction‐EBI‐UniProt‐GOA‐ACAP‐ARAP_07.06.2024_00h00	0.00	0.01	0.00	0.00	[5, 6, 7, 8]	Group6	6.06	6.00	[MAP2K2, PIK3CG, PKN1, PPP2CA, RHOA, SNCA]

Notably, it disrupts functions related to protein kinase activity and protein serine/threonine kinase activity, which are critical for various signalling pathways and cellular processes. The mutation also impacts the positive regulation of endopeptidase activity, which can influence protein degradation and processing. Additionally, the ligand‐gated monoatomic cation channel activity is altered, which may affect ion transport and neuronal signalling. Lastly, the mutation affects purine ribonucleoside triphosphate binding, a crucial function for energy metabolism and signal transduction.

##### 4.2.1.1 Targeted miRNA Interaction With APP Mutation‐Affected Networks

In addition to analysing the molecular functions affected by the APP Swedish mutation, we investigated whether the partners of APP could be regulated by miRNA molecules. Several key proteins involved in protein kinase activity, such as CDK1, FYN, GRK6, LYN, MARK4, PARK7, PKN1, PLK1, PPIA, RHOA and UCHL1, were identified as potential targets of miRNAs. Similarly, proteins like BAD, LYN and SMAD3, which play roles in the positive regulation of endopeptidase activity, and proteins involved in purine ribonucleoside triphosphate binding activity, including PLK1, PRPS1, RAC1 and RHOA, were also identified as known miRNA regulatory targets. A comprehensive list of miRNA targets for these proteins can be found in Table S7. This analysis highlights the potential for miRNA‐based regulatory mechanisms to modulate pathways disrupted by the APP Swedish mutation in AD.

Key mRNA regulators for APP and MAPT (Tau) are highlighted in Table 10. Tau mRNA can be posttranscriptionally downregulated by miR‐34c‐5p, which has been implicated in cognitive impairment and neurodegenerative processes. On the other hand, APP mRNA can be downregulated by several miRNAs, including hsa‐miR‐20a, hsa‐miR‐106b‐5p, hsa‐miR‐17‐5p and hsa‐miR‐101‐3p. Several genes directly involved in AD or participating in macromolecular complexes are regulated by miRNAs. These miRNAs are involved in regulating gene expression linked to AD pathology, suggesting potential therapeutic strategies for targeting APP and Tau expression through miRNA modulation.

**TABLE 10:** Key miRNA regulators of APP and MAPT mRNA molecules.

Regulator	Regulator target	Causality
mir‐34c‐5p	MAPT	Downregulates quantity by destabilisation
hsa‐mir‐16‐5p	APP	NA
hsa‐mir‐20a	APP	Downregulates quantity by destabilisation
hsa‐mir‐106b‐5p	APP	Downregulates quantity by destabilisation
hsa‐mir‐17‐5p	APP	Downregulates quantity by destabilisation
hsa‐mir‐101‐3p	APP	Downregulates quantity by destabilisation

#### 4.2.2 APP Cleavage Products and Interacting Partners

APP undergoes alternative splicing, resulting in the generation of multiple isoforms with distinct structural and functional properties. Four major splice variants of APP have been identified, and the protein is divided into nine structural domains [47]. Alternative splicing of exons encoding the Kunitz protease inhibitor (KPI) domain and the MRC antigen OX‐2 homologous domain leads to the formation of three major isoforms: APP770 (UniProtKB: P05067‐1), APP751 (UniProtKB: P05067‐8) and APP695 (UniProtKB: P05067‐4) [47, 48]. APP770 and APP751 isoforms are expressed in most tissues and contain the KPI domain within their extracellular regions, whereas APP695 is predominantly expressed in neurons, and it lacks the KPI domain [49]. Studies by Matsui et al. [50] have provided insights into the relevance of alternative splicing of APP in AD. They observed a two‐fold increase in APP‐KPI mRNA levels in AD, which correlated positively with glial fibrillary acidic protein (GFAP) mRNA levels. Furthermore, soluble APPα‐KPI protein levels were found to correlate with soluble Aβ40 and Aβ42 levels in AD brain tissues.

In the amyloidogenic pathway of APP processing, β‐secretase, also known as beta‐site APP cleaving enzyme 1 (BACE1), initiates the cleavage of APP, leading to the formation of a soluble N‐terminal APP fragment (sAPPβ) and a C‐terminal fragment (C99; UniProtKB: P05067‐PRO_0000000091). Subsequently, C99 is further processed by the γ‐secretase complex (CPX‐2176/CPX‐4232/CPX‐4233/CPX‐4234), resulting in the release of Aβ peptides and the APP intracellular domain (AICD) [51]. Research by Pera et al. in 2017 [52] demonstrated that C99, apart from its presence in endosomes, can also be localised in the mitochondria‐associated ER membranes (MAM). In cell models of AD, the accumulation of unprocessed C99 in MAM regions leads to an increase in sphingolipid turnover and alters the lipid composition of both MAM and mitochondrial membranes. Consequently, this alteration in mitochondrial membrane composition disrupts the assembly and activity of mitochondrial respiratory supercomplexes, potentially contributing to the bioenergetic deficits observed in AD.

#### 4.2.3 Exploring Interacting Partners: APP770, APP751 and C99 Fragment

This section explores the interacting partners of the APP770 and APP751 isoforms, as well as the C99 fragment, elucidating their functions and investigating their potential therapeutic implications in AD. A mid‐confidence MI score threshold of 0.4 was applied to obtain a reasonably‐sized, medium confidence network.

##### 4.2.3.1 APP751 (UniProtKB: P05067‐8) Partners

- The APP751 interactome is shown in Figure 7, highlighting MEP1B and SORL1 as common interacting partners shared with APP770 and C99, and identifying unique interactors such as GAP43, HOMER3, ITM2B, PSEN1, and SORT1. *MEP1B (UniProtKB: Q16820)* is a membrane metallopeptidase responsible for shedding many membrane‐bound proteins. Gindorf et al. [53] demonstrated that overexpression of Mep1b in mouse brain endothelial cells led to decreased claudin‐5 expression levels, correlating with decreased TEER values and increased permeability to [C14]‐inulin in an in vitro transwell system, indicating impairment of the blood–brain barrier (BBB) in vitro. Additionally, Marengo et al. [54] found that loss of meprin β reduced brain Aβ levels, improved cognitive abilities and rescued learning behaviour impairments in APP/lon mice.
- *SORL1 (UniProtKB: Q92673)*: SORL1 is a sorting receptor for APP, regulating its intracellular trafficking and processing into amyloidogenic‐beta peptides. SORL1 commonly interacts with both the *APP751* and *APP770* isoforms, as well as with the *C99 fragment*. It retains APP in the TGN, preventing its transit through late endosomes where Aβ peptides Aβ40 and Aβ42 are generated. Mishra et al. [55] have demonstrated that SORL1 contributes to endosomal degradation and recycling pathways in neurons, a finding with both pathogenic and therapeutic implications.

These partners are found to be interacting with APP770, APP751 isoforms and C99 fragments.

- *GAP43 (UniProtKB: P17677)*: GAP43 is associated with nerve growth and is a major component of the motile ‘growth cones’ that form the tips of elongating axons. It plays a critical role in axonal and dendritic filopodia induction. Scientific studies [56] suggest that exosomal proteins, including GAP43, can predict AD 5–7 years before the onset of cognitive impairment. Moreover, GAP43 is a potential therapeutic drug target. Geniposidic acid (CHEBI:5301) has been shown to alleviate cognitive impairment in AD mice by upregulating GAP43 through the PI3K/AKT signalling pathway [57] in mouse models.
- *HOMER3 (UniProtKB: Q9NSC5) and HOMER2 (UniProtKB: Q9NSB8)*: Parisiadou et al. [58] have provided evidence that Homer2 and Homer3 proteins interact specifically with APP. Their expression inhibits APP processing, reduces secretion of Aβ peptides and decreases the levels of cell surface APP. Moreover, they inhibit the maturation of APP and β‐secretase (BACE1). Kyratzi and Efthimiopoulos [59] demonstrated that changes in Ca^(2+)^ homeostasis affect APP‐HOMER3 interactions.
- *ITM2B (UniProtKB: Q9Y287)*: ITM2B plays a regulatory role in the processing of Aβ A4 precursor protein (APP) and acts as an inhibitor of Aβ peptide aggregation and fibril deposition. Matsuda and Senda compiled evidence demonstrating the interaction between ITM2B and APP, which leads to the inhibition of all three pathways involved in APP metabolism [60]. This suggests that ITM2B may function as a protective gene against AD.
- *PSEN1 (UniProtKB: P49768)*: Presenilins were first discovered as sites of missense mutations responsible for early‐onset AD. The catalytic subunit of the gamma‐secretase complex is an endoprotease that catalyses the intramembrane cleavage of integral membrane proteins, including Notch receptors and APP. Given its crucial role in generating the Aβ peptide, significant efforts have been directed towards the discovery and development of small‐molecule inhibitors targeting the presenilin‐containing gamma‐secretase complex as potential therapeutics for AD [61].
- *SORT1 (UniProtKB: Q99523)*: SORT1 serves as a sorting receptor within the Golgi compartment and as a clearance receptor on the cell surface. Its role is essential for protein transport from the Golgi apparatus to the endosomes. Additionally, SORT1 promotes neuronal apoptosis by facilitating the endocytosis of proapoptotic precursor forms of BDNF (proBDNF) and NGFB (proNGFB). A study by Ruan et al. [62] revealed the involvement of the intracellular domain of sortilin in regulating the nonspecific degradation of APP in mice.

##### 4.2.3.2 APP770 Isoform (UniProtKB: P05067‐1): Interactors

In addition to SORL1 and MEP1B, the APP770 isoform also shows interaction evidence with the LRPPRC protein, as shown in Figure 8.

- *LRPPRC (UniProtKB: P42704)*, also known as leucine‐rich pentatricopeptide repeat‐containing protein, exhibits a significant reduction in expression in both AD and Parkinson's disease (PD) brain samples, according to Bennett Jr. and Keeney. [63]. This reduction in LRPPRC expression suggests a decrease in mitochondrial ATP production due to compromised mitobiogenesis and mtRNA stability. Increasing brain expression of PGC‐1α and/or LRPPRC may potentially enhance the bioenergetics of AD and PD and influence the course of neurodegeneration in both conditions. Moreover, findings from Shen et al. [64] revealed distinct expressions of Lrpprc, Nefl and Sirpa, confirmed through Western blot analysis. These findings hint at initial occurrences in AD, marked by diminished energy metabolism, compromised amino acid metabolism and neurotransmitter synthesis, compensatory fatty acid metabolism, heightened expression of cytoskeletal proteins and increased oxidative stress.

##### 4.2.3.3 C99 (UniProtKB: P05067‐PRO_0000000091) Fragment

The C‐terminal fragment (C99) is generated through the action of β‐secretase 1 (BACE1) on APP, initiating the amyloidogenic pathway of APP processing. In this context, our focus is on elucidating the interactors of C99, as shown in Figure 9, and exploring their roles in the pathogenesis and potential treatment of Alzheimer's disease (AD).

- *TMCC2 (UniProtKB: O75069)* was identified as a novel apoE‐binding protein by Hopkins et al. in 2011 [65]. They found that TMCC2 also forms a complex with APP (AβPP). Coexpression of TMCC2 with apoE modified the production of Aβ from AβPPswe and AβPP‐C99 in a human cell line, suggesting that TMCC2 may facilitate the interaction of AβPP‐C99 with γ‐secretase, either directly or indirectly. In their subsequent work in 2013 [66], the same group explored the potential role of TMCC2 in neurodegeneration. They examined the *Drosophila* member of this protein family, which they named Dementin due to its critical role in brain development. They found that Dementin genetically interacted with both human APP and its *Drosophila* orthologue, the APP‐like protein (APPL). Moreover, adult flies expressing only mutated Dementin in their neurons exhibited AD‐like pathology, neurodegeneration and shortened lifespans. These findings suggest that flies with mutated Dementin mimic pathological features of early‐onset AD.
- *Gamma‐secretase complex, APH1A‐PSEN1 variant (CPX‐2176)*—This complex functions as an integral membrane aspartyl protease involved in the intramembrane cleavage of integral membrane proteins like Notch receptors. This process clears the anchors of Type I membrane proteins that remain in the membrane after shedding their ectodomain. The complex cleaves proteins with a single hydrophobic transmembrane helix and a limited‐length ectodomain. Moreover, it is responsible for generating the carboxyl terminus of the Aβ‐protein (Abeta) from the amyloid protein precursor, APP (UniProtKB: P05067). Research by Lutgarde Serneels, et al. in 2023 [67] proposed selective inhibition of specific γ‐secretase complexes, which contain either PSEN1 or PSEN2 as the catalytic subunit and APH1A or APH1B as supporting subunits. They suggested that brain‐penetrant 2‐azabicyclo[2,2,2]octane sulphonamides could offer a feasible therapeutic window in preclinical models of these disorders. This proposal comes in light of the fact that clinical development of γ‐secretases as therapeutic targets for various disorders, including cancer and AD, was discontinued due to serious mechanism‐based side effects observed in phase III trials of unselective inhibitors.

Despite being of animal origin of the two other C99 partners (Calreticulin (CALR)—Chinese hamster; Tmem30a—Mouse), we are considering their functional relevance to their human counterparts and investigating their potential relevance to AD.

- *CALR (UniprotKB: Q8K3H7)*. CALR serves as a calcium‐binding chaperone responsible for facilitating folding, oligomeric assembly and quality control within the ER through the CALR/calnexin cycle. Research by Taguchi et al. in 2000 [68] observed a decreased number of CALR‐positive cells, reduced production of CRT and lower expression of CALR mRNA in AD grey matter. CALR is believed to be a significant reservoir of releasable Ca^2+^ within the ER lumen, as suggested by Milner et al. [69]. This function underscores its potential importance in regulating free cytoplasmic Ca^2+^ concentrations, suggesting that decreased levels of CRT may contribute to disruptions in Ca^2+^ homeostasis, thereby influencing AD pathology.
- TMEM30A (*UniProtKB: Q8VEK0*), functions as an accessory component of a P4‐ATPase flippase complex. This complex catalyses the hydrolysis of ATP, coupled with the transport of aminophospholipids from the outer to the inner leaflet of various membranes. This process ensures the maintenance of the asymmetric distribution of phospholipids. In a study by Kaneshiro et al. in 2022 [70], a hypothesis was proposed suggesting that the TMEM30A/βCTF complex may impair lipid flippase formation and its activity, leading to the development of endosomal anomalies. Additionally, they found that a T‐RAP peptide derived from the βCTF binding site of TMEM30A improved endosomal anomalies, possibly by restoring lipid flippase activity. These findings shed light on the mechanisms underlying vesicular traffic impairment and suggest a potential therapeutic target for AD.

### 4.3 Pathway Enrichment Analysis of Tau‐F and Mutant Tau‐F Interactors in Reactome

#### 4.3.1 Analysis of Tau‐F Interactors Pathway Enrichment

We selected the top 25 enriched pathways from the Tau‐F high‐confidence interactome based on entity ratio, FDR, and p‐values. A ReacFoam visualization of all human pathways enriched for Tau‐F interactors is shown in Figure 10. We achieved high enrichment mapping for multiple pathways related to Metabolism of Proteins (R‐HSA‐392499), particularly from the Translation (R‐HSA‐392499) associated pathway (*R‐HSA‐72649, R‐HSA‐72702, R‐HSA‐72695, R‐HSA‐72662, R‐HSA‐72706, R‐HSA‐156827, R‐HSA‐72689, R‐HSA‐72613, R‐HSA‐72737, R‐HSA‐156902, R‐HSA‐156842, R‐HSA‐1799339*). Tau is known to regulate translational machinery in Alzheimer pathology in multiple ways. In particular, tau interacts with ribosomal protein S6 (rpS6 or S6), which is a crucial regulator of translation, and as a consequence, reduced protein synthesis has been observed under tauopathy conditions in AD (Koren et al., 2019). Tau aggregates have been observed to sequester eukaryotic ribosomes by forming tau‐ribosome aggregates, resulting in suppression of translation (Banerjee et al. 2020). It has also been observed that expression of human tau (hTau) decreases both protein synthesis and biogenesis of the 60S ribosomal subunit. In the presence of FTD‐associated tau mutations, this effect gets further aggravated (Evans et al. 2021).

We also observed enrichment mapping of the pathway related to the Metabolism of RNA (R‐HSA‐8953854) pathways regulating nonsense‐mediated decay (NMD) of mRNA (*R‐HSA‐975956, R‐HSA‐927802,R‐HSA‐975957*). The role of NMD in the pathology of neurodegenerative disease is observed in multiple recent studies (Howe et al. 2023 and Patani et al. 2023), and a recent study by Zuniga et al. (2022) highlights the tau induced deficit in nonsense mediated mRNA decay that contributes to neurodegeneration. Additionally the *PRMT5 methylosome complex* (*CLNS1A variant* CPX‐696) is enriched exclusively in the Tau‐F interactome, compared to both mutant Tau‐F networks. This complex is known to play a crucial role in regulating mRNA splicing and NMD of mRNAs (Radzisheuskaya et al. 2019; Rengasamy et al. 2017).

Further, we obtained enrichment mapping of the pathway related to the nervous system development (R‐HSA‐9675108) including Axon guidance (R‐HSA‐422475) regulated by ROBO signalling (R‐HSA‐376176). The role of Axon guidance and ROBO signalling in AD pathology is very well studied and discussed in Zhang et al. (2021) and Wang et al. (2017).

We also identified pathways uniquely enriched in Tau‐F compared to the V337M and P301L mutants, with the corresponding ReacFoam visualizations shown in Figures 11 and 12. Again, only pathways with *p* values lower than 0.05 are considered for this analysis. Table 11 contains the list of unique WT‐enriched pathways compared to each mutant type.

**TABLE 11:** Pathways exclusively enriched in Tau‐F relative to V337 and P301L mutant forms.

Unique Tau‐F pathways compared to V337M	Unique Tau‐F pathways compared to P301L
Complex III assembly	Mitotic prophase
Initiation of nuclear envelope (NE) reformation	Ca‐dependent events
Mitochondrial protein import	RAC2 GTPase cycle
Protein localisation	Late endosomal microautophagy
Endosomal sorting complex required for transport (ESCRT)	RHOD GTPase cycle
Mitotic prophase	CaM pathway
Negative regulation of MET activity	Calmodulin‐induced events
Regulation of gap junction activity	DAG and IP3 signalling
RAC3 GTPase cycle	Signalling by nuclear receptors
RAC2 GTPase cycle	Unfolded protein response (UPR)
Late endosomal microautophagy	SARS‐CoV‐2 modulates autophagy
Protein‐protein interactions at synapses	Complex IV assembly
RHOD GTPase cycle	RMTs methylate histone arginines
Axonal growth inhibition (RHOA activation)	Budding and maturation of HIV virion
Signalling by nuclear receptors	Long‐term potentiation
Biogenic amines are oxidatively deaminated to aldehydes by MAOA and MAOB	RNA polymerase I promoter opening
Metabolism of serotonin	Signalling by ERBB4
Unfolded protein response (UPR)	Assembly of the ORC complex at the origin of replication
Complex IV assembly	G beta:gamma signalling through PLC beta
RMTs methylate histone arginines	PKA‐mediated phosphorylation of CREB
Budding and maturation of HIV virion	G beta:gamma signalling through CDC42
EGFR downregulation	Prostacyclin signalling through prostacyclin receptor
RNA polymerase I promoter opening	CaMK IV‐mediated phosphorylation of CREB
Signalling by ERBB4	Integration of provirus
Assembly of the ORC complex at the origin of replication	IRE1alpha activates chaperones
G beta:gamma signalling through PLC beta	ERCC6 (CSB) and EHMT2 (G9a) positively regulate rRNA expression
G beta:gamma signalling through CDC42	Unblocking of NMDA receptors, glutamate binding and activation
Prostacyclin signalling through prostacyclin receptor	Negative regulation of NMDA receptor‐mediated neuronal transmission
p75NTR regulates axonogenesis	Presynaptic function of Kainate receptors
CaMK IV‐mediated phosphorylation of CREB	Signalling by PDGFR in disease
Integration of provirus	DNA methylation
IRE1alpha activates chaperones	Regulation of pyruvate metabolism
ERCC6 (CSB) and EHMT2 (G9a) positively regulate rRNA expression	Nef mediated downregulation of MHC class I complex cell surface expression
Unblocking of NMDA receptors, glutamate binding and activation	Pexophagy
Negative regulation of NMDA receptor‐mediated neuronal transmission	B‐WICH complex positively regulates rRNA expression
Presynaptic function of kainate receptors	Nuclear events stimulated by ALK signalling in cancer
Signalling by PDGFR in disease	Integrin signalling
Signalling by SCF‐KIT	Adrenaline, noradrenaline inhibits insulin secretion
DNA methylation	Positive epigenetic regulation of rRNA expression
Regulation of pyruvate metabolism	PRC2 methylates histones and DNA
Pexophagy	Signalling by FGFR1 in disease
Apoptotic cleavage of cell adhesion proteins	Defective pyroptosis
B‐WICH complex positively regulates rRNA expression	Activated PKN1 stimulates transcription of AR (androgen receptor) regulated genes KLK2 and KLK3
Integrin signalling	Nephrin family interactions
Adrenaline, noradrenaline inhibits insulin secretion	
Serotonin clearance from the synaptic cleft	
Positive epigenetic regulation of rRNA expression	
PRC2 methylates histones and DNA	
Signalling by FGFR1 in disease	
Defective pyroptosis	
Activated PKN1 stimulates transcription of AR (androgen receptor) regulated genes KLK2 and KLK3	
Nephrin family interactions	

Many of these Tau‐F specific pathways align well with the protein complexes identified using interactome analysis. For example:

*RMTs methylate histone arginines (R‐HSA‐3214858)*. This pathway is involved in the maintenance of chromatin organisation and is not detected in the mutant Tau‐F specific pathways. The *RMTs methylate histone arginines pathway* (R‐HSA‐3214858) plays a crucial role in maintaining chromatin organisation through PTMs carried out by arginine methyltransferases (PRMTs). This pathway, which is not detected in the mutant Tau‐F‐specific pathways, is vital for regulating gene expression and chromatin dynamics. PRMT activity can be modulated through various mechanisms, including PTMs, association with regulatory proteins, subcellular localisation and factors that influence enzyme‐substrate interactions. The methylation sites targeted by PRMTs are also affected by the presence of other PTMs on their substrates, highlighting the complex regulation of chromatin architecture and gene expression.

Basso and Pennuto [71] demonstrate that both loss and gain of PRMT function are implicated in the pathogenesis of neurodegenerative diseases, underscoring a significant role for arginine methylation in neurodegeneration. This observation aligns with other complexes that are not detected in the mutant Tau interactomes. For example, complexes involved in chromatin organisation, such as nucleosomes (CPX‐2556, CPX‐2564, CPX‐5668) and CENP‐A Nucleosome (CPX‐5647), were identified only in the Tau‐F interaction network. Furthermore, the PRMT5 methylosome complex, CLNS1A variant (CPX‐696) is also absent, suggesting a broader disruption of chromatin‐related processes in the mutant Tau networks.

*Budding and maturation of HIV virion (R‐HSA‐162588)* is part of the infectious disease family. This process is critical in the life cycle of HIV, where viral components are assembled at the inner leaflet of the host cell's plasma membrane. Once these viral components are assembled, the host's cellular machinery facilitates viral budding. The virus takes advantage of the host ESCRT pathway to terminate Gag polymerisation and catalyse release. The ESCRT pathway is normally responsible for membrane fission that creates cytoplasm‐filled vesicular bodies. In this case, HIV (and other viruses) take advantage of the ESCRT cellular machinery to facilitate virion budding from the host. It is important to note that the ESCRT complex (CPX‐329) was enriched exclusively in the Tau‐F interaction network.

*RHOD (R‐HSA‐9013405) and RAC2 GTPase (R‐HSA‐9013404) cycle pathways*. These pathways belong to Rho GTPases family proteins involved in the intricate machinery of intracellular membrane trafficking by localising to the membranes of different organelles and interacting with effectors such as sorting adaptors, tethering factors, kinases, phosphatases and tubular‐vesicular cargo. Dysregulation of RhoGTPases has been implicated in the pathogenesis of AD. Studies have observed alterations in the expression and activity of specific RAB proteins in the brains of individuals with AD (Bolognon et al. 2014; Cai et al. 2021). Modulators of Rho GTPases represent potential targets for drug development aimed at mitigating the aberrant cellular processes associated with AD (Aguilar et al. 2017).

#### 4.3.2 Analysis of V337M Tau‐F Mutant Interactome Specific Pathway Enrichment

Using the V337M Tau‐F mutant specific interactome, we got a detailed pathway enrichment report containing pathways, which were common between Tau‐F and V337M Tau‐F mutant along with pathway that got enriched only in V337M Tau‐F mutant compared to WT enriched pathway (https://drive.google.com/drive/folders/1najDB9Kya5fAvy4jbCUBldBzZNO0QXra). We will focus mainly on the pathways specific to the V337M Tau‐F mutant.

Several distinct pathways enriched within the V337M Tau‐F interactome closely correspond with the complexes enriched in the same interactome.

*Transcription‐coupled nucleotide excision repair (R‐HSA‐6781827)*: The V337M network is enriched with components involved in transcription‐coupled nucleotide excision repair (TC‐NER), a critical pathway for repairing DNA damage in actively transcribed genes. Key components of the TC‐NER preincision complex, such as XPA and the XAB2 complex, are involved in this network. The XAB2 complex not only contributes to pre‐mRNA splicing but also modulates the structure of nascent mRNA hybrids with template DNA through its RNA‐DNA helicase activity, which is essential for proper DNA damage processing. Studies like those by Jensen et al. [72] have highlighted that alterations in the expression of NER components between different brain regions are associated with AD, suggesting a unique role for NER in brain function and AD pathogenesis. Additionally, research by Miller et al. [73] using single‐cell whole‐genome sequencing (scWGS) in neurons from AD and control brains reveals that genes actively expressed in neurons show signature‐specific damage and transcriptional strand bias.

In the analysis of V337M‐specific complex enrichment, we found that PRP19‐CDC5L (CPX‐5824) complex stands out, which plays a role in regulating DDR. Thus, the interactome and pathway enrichment data supports the possible role of PRP19‐CDC5L and DDR in the pathogenesis of AD and tau biology.

*Immune system pathways and Metabolic pathways*: We got specific enrichment of multiple *Immune system pathways* including Nef‐mediated downregulation of MHC Class I complex cell surface expression (R‐HSA‐164940), Toll Like receptor 4 (TLR4) Cascade (R‐HSA‐166016), respiratory syncytial virus infection pathway (R‐HSA‐9820952), MyD88 cascade initiated on plasma membrane (R‐HSA‐975871), Toll like receptor 5 (TLR5) Cascade (R‐HSA‐168176), Toll like receptor 10 (TLR10) Cascade (R‐HSA‐168142), MyD88:MAL(TIRAP) cascade initiated on plasma membrane (R‐HSA‐166058), Toll like receptor TLR6:TLR2 Cascade (R‐HSA‐168188), Toll like receptor 2 (TLR2) Cascade (TLR2 Cascade), Toll‐like receptor cascades (R‐HSA‐168898), MyD88 dependent cascade initiated on endosome (R‐HSA‐975155), ERKs are inactivated (R‐HSA‐202670) and maturation of hRSV A proteins (R‐HSA‐9828806), *metabolism related pathways* such as HuR (ELAVL1) binds and stabilises mRNA (R‐HSA‐450520), PKA‐mediated phosphorylation of key metabolic factors (R‐HSA‐163358), triglyceride catabolism (R‐HSA‐163560) and plasma lipoprotein assembly (R‐HSA‐8963898).

The V337M mutant interactome reveals the exclusive presence of *CRL4‐CRBN E3 ubiquitin ligase* family *complexes* (CPX‐2759, CPX‐2762) regulating a broad spectrum of biological processes, encompassing ion channel regulation, cancer development, immune regulation and energy metabolism regulation, which can be correlated well with the enriched pathways for the V337 Tau‐F specific interactome.

#### 4.3.3 Analysis of P301L Tau‐F Mutant Interactome Specific Pathway Enrichment

Similar to V337M enrichment analysis, using the P301L Tau‐F mutant specific interactome, we generated a detailed pathway enrichment report containing pathways that were common between Tau‐F and P301L mutant along with pathway that got enriched only in P301L Tau‐F mutant compared to WT enriched pathway (Table 11). We will focus mainly on the pathways specific to the P301L Tau‐F mutant.

Similar to V337M analysis, we have specific enrichment of multiple *Immune system pathways* such as MyD88 cascade initiated on plasma membrane (R‐HSA‐975871), TLR10 Cascade (R‐HSA‐168142), TLR5 Cascade (R‐HSA‐168176), TLR4 Cascade (R‐HSA‐166016), TRAF6‐mediated induction of NFkB and MAP kinases upon TLR7/8 or 9 activation (R‐HSA‐975138), interleukin‐17 signalling (R‐HSA‐448424), ISG15 antiviral mechanism (R‐HSA‐1169408), MyD88 dependent cascade initiated on endosome (R‐HSA‐975155), ERKs are inactivated (R‐HSA‐202670) Toll like receptor 7/8 (TLR7/8) Cascade and *Disease pathways* including RAS signalling downstream of NF1 loss‐of‐function variants (R‐HSA‐6802953), FCGR3A‐mediated phagocytosis (R‐HSA‐9664422), respiratory syncytial virus infection pathway (R‐HSA‐9820952), parasite infection (R‐HSA‐9664407) and Leishmania phagocytosis (R‐HSA‐9664417).

The Top 10 pathways which got enriched specifically with P301L pathway are: Sema4D in semaphorin signalling (R‐HSA‐400685), Transferrin endocytosis and recycling (R‐HSA‐917977), Regulation of CDH11 function (R‐HSA‐9762292), Sema4D induced cell migration and growth‐cone collapse (R‐HSA‐416572), EPH‐ephrin mediated repulsion of cells (R‐HSA‐3928665), SUMOylation of transcription cofactors (R‐HSA‐3899300), Transport of Mature mRNA derived from an Intron‐Containing Transcript (R‐HSA‐159236), Nucleotide‐binding domain, leucine‐rich repeat containing receptor (NLR) signalling pathways (R‐HSA‐168643), Transport of Mature Transcript to Cytoplasm (R‐HSA‐72202) and Regulation of CDH11 Expression and Function (R‐HSA‐9759475).

Based on interactome analysis, the P301L mutation of Tau‐F shows the presence of PRMT5 methylosome complex, COPR5 variant (CPX‐8149), which is known to play a role in the methylation of arginine residues, including Histone 4 R8 and Histone 4 R3, regulating chromatin remodelling and repression of the CCNE1 gene. Since chromatin remodelling is known to regulate multiple genes/proteins in a context‐specific manner, suggesting there is a strong possibility of pathway enrichment being driven by this mechanism.

## 5 Conclusion

Our differential interactome analysis, centred on clinical variants in AD, demonstrates how specific mutations disrupt protein networks and cellular processes, offering insights for variant‐guided therapeutic strategies. By leveraging AP‐MS‐derived contextual interactomes, we created a reference framework to assess the functional consequences of disease‐associated variants.

Both P301L and V337M variants show disruptions in chromatin organisation, supported by Reactome pathway enrichment analysis. In particular, the ‘RMTs methylate histone arginines’ pathway—driven by protein arginine methyltransferases (PRMTs)—was affected in both networks, highlighting a shared perturbation in epigenetic regulation via posttranslational histone modification.

Additionally, autophagy‐related disruption was observed, particularly through loss of ESCRT complexes, which are essential for endosomal sorting and membrane fission processes. Enrichment of the ‘HIV life cycle—virion budding’ pathway further reflects how the hijacking of ESCRT machinery by viruses parallels neurodegenerative mechanisms in AD. Dysregulation of Rho GTPases (e.g., RHOD and RAC2), involved in vesicle trafficking and cytoskeletal dynamics, also emerged, linking cytoskeletal integrity and endosomal dysfunction to AD pathogenesis.

In the P301L variant network, key deficiencies were identified in:

- Chromatin remodelling (loss of nucleosome complexes),
- Protein homeostasis (loss of VCP‐NPL4 AAA ATPase and vacuolar proton‐translocating ATPase complexes) and
- Synaptic vesicle function and ion balance.

Splicing regulation was compromised due to the absence of the PRMT5 methylosome complex, although expression of the COPR5 complex might offer partial compensation. The functional consequences of this compensation require further validation.

In contrast, the V337M variant preserved greater network integrity. Disrupted processes included:

- Protein turnover (via VCP‐NPL4),
- Ion homeostasis (via sodium:potassium‐exchanging ATPases) and
- Chromosomal maintenance.

Importantly, splicing regulation remained relatively intact, likely due to the presence of the PRP19‐CDC5L complex. Furthermore, the enrichment of the CRL4‐CRBN E3 ubiquitin ligase complex suggests a buffering role, potentially offering a protective mechanism in the V337M context.

Together, these findings underscore that P301L elicits more severe and immediate perturbations than V337M, reinforcing the need for variant‐specific network analysis in developing precision therapies for AD.

### 5.1 APP Interaction Network: Therapeutic Insights

Analysis of APP physical interactors revealed several actionable targets with therapeutic potential:

- MEP1B cleaves APP to release neurotoxic Aβ peptides—a critical node for intervention.
- SORL1 retains APP in the TGN, preventing amyloidogenic cleavage; its upregulation is considered protective.
- GAP43, which binds the C99 fragment of APP, is neuroprotective. Geniposidic acid has been shown to upregulate GAP43 via PI3K/AKT signalling and improve cognitive function in AD models.
- HOMER2 and HOMER3 inhibit APP processing and reduce Aβ secretion.
- ITM2B interacts with APP to inhibit all three processing pathways, suggesting it acts as a protective gene.
- PSEN1, a catalytic component of the γ‐secretase complex, continues to be a focal point for small‐molecule therapeutic development.
- LRPPRC, downregulated in AD, is linked to mitochondrial dysfunction and may influence APP metabolism.

In addition to protein‐based interactions, miRNA‐mediated regulation adds another important dimension to AD‐related molecular networks:

- Tau mRNA is negatively regulated by miR‐34c‐5p
- APP mRNA is subject to posttranscriptional downregulation by several miRNAs, including hsa‐miR‐20a, hsa‐miR‐106b‐5p, hsa‐miR‐17‐5p and hsa‐miR‐101‐3p, suggesting possible therapeutic avenues to reduce APP expression and Aβ production.

Beyond Tau and APP, multiple genes directly involved in AD or participating in disease‐relevant macromolecular complexes are regulated by miRNAs, highlighting the broader impact of posttranscriptional gene regulation in AD pathogenesis.

Collectively, these insights reinforce a multilayered therapeutic landscape, where targeting physical protein interactions, modulating complex assembly and regulating mRNA via miRNAs could converge to slow or alter AD progression.

## Conflicts of Interest

The authors declare no conflicts of interest.

## Supporting information




**Supporting File 1**: pmic70018‐sup‐0007‐tableS1.txt.

**Supporting File 2**: pmic70018‐sup‐0001‐tableS2.txt.

**Supporting File 3**: pmic70018‐sup‐0001‐tableS3.txt.

**Supporting File 4**: pmic70018‐sup‐0001‐tableS4.xlsx.

**Supporting File 5**: pmic70018‐sup‐0001‐tableS5.xlsx.

**Supporting File 6**: pmic70018‐sup‐0001‐tableS6.xlsx.

**Supporting File 7**: pmic70018‐sup‐0001‐tableS7.xlsx.

## Data Availability

The data that support the findings of this study are available in [IntAct at [https://www.ebi.ac.uk/intact/home], reference number [reference number]. These data were derived from the following resources available in the public domain: [https://www.ebi.ac.uk/intact/search?query=annot:%22dataset:alzheimers%22]

## References

[1] P. Porras , E. Barrera , A. Bridge , et al., “Towards a Unified Open Access Dataset of Molecular Interactions,” Nature Communications 11, no. 1 (2020): 6144. 10.1038/s41467-020-19942-z PMC7708836 33262342

[2] N. Del Toro , A. Shrivastava , E. Ragueneau , et al., “The IntAct Database: Efficient Access to Fine‐Grained Molecular Interaction Data,” Nucleic Acids Research 50, no. D1 (2022): D648–D653. 34761267 10.1093/nar/gkab1006 PMC8728211

[3] L. Licata , L. Briganti , D. Peluso , et al., “MINT, the Molecular Interaction Database: 2012 Update,” Nucleic Acids Research 40, no. Database issue (2012): D857–D861. 22096227 10.1093/nar/gkr930 PMC3244991

[4] O. Clerc , M. Deniaud , S. D. Vallet , et al., “MatrixDB: Integration of New Data With a Focus on Glycosaminoglycan Interactions,” Nucleic Acids Research 47, no. D1 (2019): D376–D381. 30371822 10.1093/nar/gky1035 PMC6324007

[5] UniProt Consortium . “UniProt: The Universal Protein Knowledgebase in 2023,” Nucleic Acids Research 51, no. D1 (2023): D523–D531. 36408920 10.1093/nar/gkac1052 PMC9825514

[6] L. Salwinski , “The Database of Interacting Proteins: 2004 Update,” Nucleic Acids Research 32, no. Database issue (2004): 449D–451D. 10.1093/nar/gkh086 PMC308820 14681454

[7] V. M. Perreau , S. Orchard , P. A. Adlard , et al., “A Domain Level Interaction Network of Amyloid Precursor Protein and Aβ of Alzheimer's Disease,” Proteomics 10, no. 12 (2010): 2377–2395. 20391539 10.1002/pmic.200900773

[8] P. Porras , M. Duesbury , A. Fabregat , et al., “A Visual Review of the Interactome of LRRK2: Using Deep‐Curated Molecular Interaction Data to Represent Biology,” Proteomics 15, no. 8 (2015): 1390–1404. 25648416 10.1002/pmic.201400390 PMC4415485

[9] L. Breuza , C. N. Arighi , G. Argoud‐Puy , et al., “A Coordinated Approach by Public Domain Bioinformatics Resources to Aid the Fight Against Alzheimer's Disease Through Expert Curation of Key Protein Targets,” Journal of Alzheimer's Disease 77, no. 1 (2020): 257–273. 10.3233/JAD-200206 PMC7592670 32716361

[10] Y. Li , “The Tandem Affinity Purification Technology: An Overview,” Biotechnology Letters 33, no. 8 (2011): 1487–1499. 21424840 10.1007/s10529-011-0592-x

[11] J. E. Habel , “Biotin Proximity Labeling for Protein‐Protein Interaction Discovery: The BioID Method,” Methods in Molecular Biology 2261 (2021): 357–379. 33421001 10.1007/978-1-0716-1186-9_22

[12] IMEx Consortium Curators . N. Del‐Toro , M. Duesbury , et al., “Capturing Variation Impact on Molecular Interactions in the IMEx Consortium Mutations Data Set,” Nature Communications 10, no. 1 (2019): 10. 10.1038/s41467-018-07709-6 PMC6315030 30602777

[13] B. H. M. Meldal , L. Perfetto , C. Combe , et al., “Complex Portal 2022: New Curation Frontiers,” Nucleic Acids Research 50, no. D1 (2022): D578–D586. 34718729 10.1093/nar/gkab991 PMC8689886

[14] G. Bindea , B. Mlecnik , H. Hackl , et al., “ClueGO: A Cytoscape Plug‐in to Decipher Functionally Grouped Gene Ontology and Pathway Annotation Networks,” Bioinformatics 25, no. 8 (2009): 1091–1093. 19237447 10.1093/bioinformatics/btp101 PMC2666812

[15] E. Drummond , G. Pires , C. MacMurray , et al., “Phosphorylated Tau Interactome in the Human Alzheimer's Disease Brain,” Brain 143, no. 9 (2020): 2803–2817. 32812023 10.1093/brain/awaa223 PMC7526722

[16] S. Meier , M. Bell , D. N. Lyons , et al., “Identification of Novel Tau Interactions With Endoplasmic Reticulum Proteins in Alzheimer's Disease Brain,” Journal of Alzheimer's Disease 48, no. 3 (2015): 687–702. 10.3233/JAD-150298 PMC4881838 26402096

[17] T. E. Tracy , J. Madero‐Pérez , D. L. Swaney , et al., “Tau Interactome Maps Synaptic and Mitochondrial Processes Associated with Neurodegeneration,” Cell 185, no. 4 (2022): 712–728.e14. 35063084 10.1016/j.cell.2021.12.041 PMC8857049

[18] B. A. Cottrell , V. Galvan , S. Banwait , et al., “A Pilot Proteomic Study of Amyloid Precursor Interactors in Alzheimer's Disease,” Annals of Neurology 58, no. 2 (2005): 277–289. 16049941 10.1002/ana.20554 PMC1847583

[19] K. H. Strang , T. E. Golde , and B. I. Giasson , “MAPT Mutations, Tauopathy, and Mechanisms of Neurodegeneration,” Laboratory Investigation 99, no. 7 (2019): 912–928. 30742061 10.1038/s41374-019-0197-x PMC7289372

[20] M. Goedert , D. S. Eisenberg , and R. A. Crowther , “Propagation of Tau Aggregates and Neurodegeneration,” Annual Review of Neuroscience 40, no. July (2017): 189–210. 10.1146/annurev-neuro-072116-031153 28772101

[21] D. Panda , J. C. Samuel , M. Massie , S. C. Feinstein , and L. Wilson , “Differential Regulation of Microtubule Dynamics by Three‐ and Four‐Repeat Tau: Implications for the Onset of Neurodegenerative Disease,” Proceedings of the National Academy of Sciences of the United States of America 100, no. 16 (2003): 9548–9553. 12886013 10.1073/pnas.1633508100 PMC170955

[22] I. Grundke‐Iqbal , K. Iqbal , Y. C. Tung , M. Quinlan , H. M. Wisniewski , and L. I. Binder , “Abnormal Phosphorylation of the Microtubule‐Associated Protein Tau (tau) in Alzheimer Cytoskeletal Pathology,” Proceedings of the National Academy of Sciences of the United States of America 83, no. 13 (1986): 4913–4917. 3088567 10.1073/pnas.83.13.4913 PMC323854

[23] L. Gil , S. A. Niño , C. Guerrero , and M. E. Jiménez‐Capdeville , “Phospho‐Tau and Chromatin Landscapes in Early and Late Alzheimer's Disease,” International Journal of Molecular Sciences 22, no. 19 (2021): 10283. 34638632 10.3390/ijms221910283 PMC8509045

[24] T. Suganuma , S. K. Swanson , M. Gogol , et al., “MPTAC Determines APP Fragmentation via Sensing Sulfur Amino Acid Catabolism,” Cell Reports 24, no. 6 (2018): 1585–1596. 30089268 10.1016/j.celrep.2018.07.013

[25] J.‐A. Lee , A. Beigneux , S. T. Ahmad , S. G. Young , and F.‐B. Gao , “ESCRT‐III Dysfunction Causes Autophagosome Accumulation and Neurodegeneration,” Current Biology 17, no. 18 (2007): 1561–1567. 17683935 10.1016/j.cub.2007.07.029

[26] M. M. Esiri , S. C. Biddolph , and C. S. Morris , “Prevalence of Alzheimer Plaques in AIDS,” Journal of Neurology, Neurosurgery, and Psychiatry 65, no. 1 (1998): 29–33. 9667557 10.1136/jnnp.65.1.29 PMC2170157

[27] M. F. Sathler , M. J. Doolittle , J. A. Cockrell , et al., “HIV and FIV Glycoproteins Increase Cellular Tau Pathology via cGMP‐Dependent Kinase II Activation,” Journal of Cell Science 135, no. 12 (2022): jcs259764, 10.1242/jcs.259764. PMC9270957 35638570

[28] M. Vijayan , L. Yin , P. H. Reddy , and K. Benamar , “Behavioral Evidence for a Tau and HIV‐gp120 Interaction,” International Journal of Molecular Sciences 23, no. 10 (2022): 5514, 10.3390/ijms23105514. 35628323 PMC9146203

[29] C. Mengus , M. Neutzner , A. C. P. F. Bento , et al., “VCP/p97 Cofactor UBXN1/SAKS1 Regulates Mitophagy by Modulating MFN2 Removal from Mitochondria,” Autophagy 18, no. 1 (2022): 171–190. 33966597 10.1080/15548627.2021.1922982 PMC8865314

[30] C. Xie , Y. Aman , B. A. Adriaanse , et al., “Culprit or Bystander: Defective Mitophagy in Alzheimer's Disease,” Frontiers in Cell and Developmental Biology 7 (2019): 391. 32010698 10.3389/fcell.2019.00391 PMC6978796

[31] Y. Gwon , B. A. Maxwell , R.‐M. Kolaitis , P. Zhang , H. J. Kim , and J. P. Taylor , “Ubiquitination of G3BP1 Mediates Stress Granule Disassembly in a Context‐Specific Manner,” Science 372, no. 6549 (2021): abf6548. 10.1126/science.abf6548 PMC8574224 34739333

[32] R. Ganji , S. Mukkavalli , F. Somanji , and M. Raman , “The VCP‐UBXN1 Complex Mediates Triage of Ubiquitylated Cytosolic Proteins Bound to the BAG6 Complex,” Molecular and Cellular Biology 38, no. 13 (2018): e00154‐18, 10.1128/MCB.00154-18. PMC6002697 29685906

[33] C. Meyer , D. Zizioli , S. Lausmann , et al., “μ1A‐Adaptin‐Deficient Mice: Lethality, Loss of AP‐1 Binding and Rerouting of Mannose 6‐Phosphate Receptors,” The EMBO Journal 19, no. 10 (2000): 2193–2203. 10811610 10.1093/emboj/19.10.2193 PMC384363

[34] S. Poirier , G. Mayer , S. R. Murphy , et al., “The Cytosolic Adaptor AP‐1A Is Essential for the Trafficking and Function of Niemann‐Pick Type C Proteins,” Traffic (Copenhagen, Denmark) 14, no. 4 (2013): 458–469. 23350547 10.1111/tra.12046 PMC3607445

[35] A. Salminen , K. Kaarniranta , A. Kauppinen , et al., “Impaired Autophagy and APP Processing in Alzheimer's Disease: The Potential Role of Beclin 1 Interactome,” Progress in Neurobiology 106–107, no. July (2013): 33–54. 10.1016/j.pneurobio.2013.06.002 23827971

[36] P. Lo Surdo , M. Iannuccelli , S. Contino , et al., “SIGNOR 3.0, the SIGnaling Network Open Resource 3.0: 2022 Update,” Nucleic Acids Research 51, no. D1 (2023): D631–D637. 36243968 10.1093/nar/gkac883 PMC9825604

[37] H. Higashida , S. Yokoyama , C. Tsuji , and S.‐I. Muramatsu , “Neurotransmitter Release: Vacuolar ATPase V0 Sector c‐Subunits in Possible Gene or Cell Therapies for Parkinson's, Alzheimer's, and Psychiatric Diseases,” The Journal of Physiological Sciences 67, no. 1 (2017): 11–17. 27289535 10.1007/s12576-016-0462-3 PMC10717279

[38] S.‐H. Kim , Y.‐S. Cho , Y. Kim , et al., “Endolysosomal Impairment by Binding of Amyloid Beta or MAPT/Tau to V‐ATPase and Rescue via the HYAL‐CD44 Axis in Alzheimer Disease,” Autophagy 19, no. 8 (2023): 2318–2337. 36843263 10.1080/15548627.2023.2181614 PMC10351450

[39] S. Bonnal , L. Vigevani , and J. Valcárcel , “The Spliceosome as a Target of Novel Antitumour Drugs,” Nature Reviews Drug Discovery 11, no. 11 (2012): 847–859. 23123942 10.1038/nrd3823

[40] A. Sidarovich , C. L. Will , M. M. Anokhina , et al., “Identification of a Small Molecule Inhibitor That Stalls Splicing at an Early Step of Spliceosome Activation,” eLife 6, no. March (2017): e23533, 10.7554/eLife.23533. PMC5354520 28300534

[41] S. E. Swalley , “Expanding Therapeutic Opportunities for Neurodegenerative Diseases: A Perspective on the Important Role of Phenotypic Screening,” Bioorganic & Medicinal Chemistry 28, no. 3 (2020): 115239. 31889605 10.1016/j.bmc.2019.115239

[42] J. Holzmann and W. Rossmanith , “tRNA Recognition, Processing, and Disease: Hypotheses around an Unorthodox Type of RNase P in Human Mitochondria,” Mitochondrion 9, no. 4 (2009): 284–288. 19376274 10.1016/j.mito.2009.03.008

[43] D. Del Prete , R. C. Rice , A. M. Rajadhyaksha , and L. D'adamio , “Amyloid Precursor Protein (APP) May Act as a Substrate and a Recognition Unit for CRL4CRBN and Stub1 E3 Ligases Facilitating Ubiquitination of Proteins Involved in Presynaptic Functions and Neurodegeneration,” The Journal of Biological Chemistry 291, no. 33 (2016): 17209–17227. 27325702 10.1074/jbc.M116.733626 PMC5016122

[44] B. Zdrazil , E. Felix , F. Hunter , et al., “The ChEMBL Database in 2023: A Drug Discovery Platform Spanning Multiple Bioactivity Data Types and Time Periods,” Nucleic Acids Research 52, no. D1 (2024): D1180–D1192. 37933841 10.1093/nar/gkad1004 PMC10767899

[45] M. Lacroix , S. El Messaoudi , G. Rodier , A. Le Cam , C. Sardet , and E. Fabbrizio , “The Histone‐Binding Protein COPR5 Is Required for Nuclear Functions of the Protein Arginine Methyltransferase PRMT5,” EMBO Reports 9, no. 5 (2008): 452–458. 18404153 10.1038/embor.2008.45 PMC2373370

[46] A. Koff , F. Cross , A. Fisher , et al., “Human Cyclin E, a New Cyclin That Interacts with Two Members of the CDC2 Gene Family,” Cell 66, no. 6 (1991): 1217–1228. 1833068 10.1016/0092-8674(91)90044-y

[47] H. A. Rohan De Silva , A. Jen , C. Wickenden , L.‐S. Jen , S. L. Wilkinson , and A. J. Patel , “Cell‐Specific Expression of β‐Amyloid Precursor Protein Isoform mRNAs and Proteins in Neurons and Astrocytes,” Molecular Brain Research 47, no. 1‐2 (1997): 147–156. 9221912 10.1016/s0169-328x(97)00045-4

[48] F. De Sauvage and J.‐N. Octave , “A Novel mRNA of the A4 Amyloid Precursor Gene Coding for a Possibly Secreted Protein,” Science 245, no. 4918 (1989): 651–653. 2569763 10.1126/science.2569763

[49] Y.‐W. Zhang , R. Thompson , H. Zhang , and H. Xu , “APP Processing in Alzheimer's Disease,” Molecular Brain 4, no. January (2011): 3. 21214928 10.1186/1756-6606-4-3 PMC3022812

[50] T. Matsui , M. Ingelsson , H. Fukumoto , et al., “Expression of APP Pathway mRNAs and Proteins in Alzheimer's Disease,” Brain Research 1161, no. August (2007): 116–123. 17586478 10.1016/j.brainres.2007.05.050

[51] Y. Cho , H.‐G. Bae , E. Okun , T. V. Arumugam , and D.‐G. Jo , “Physiology and Pharmacology of Amyloid Precursor Protein,” Pharmacology & Therapeutics 235, no. July (2022): 108122. 35114285 10.1016/j.pharmthera.2022.108122

[52] M. Pera , D. Larrea , C. Guardia‐Laguarta , et al., “Increased Localization of APP‐C99 in Mitochondria‐Associated ER Membranes Causes Mitochondrial Dysfunction in Alzheimer Disease,” The EMBO Journal 36, no. 22 (2017): 3356–3371. 29018038 10.15252/embj.201796797 PMC5731665

[53] M. Gindorf , S. E. Storck , A. Ohler , F. Scharfenberg , C. Becker‐Pauly , and C. U. Pietrzik , “Meprin β: A Novel Regulator of Blood–Brain Barrier Integrity,” Journal of Cerebral Blood Flow and Metabolism 41, no. 1 (2021): 31–44. 32065075 10.1177/0271678X20905206 PMC7747169

[54] L. Marengo , F. Armbrust , C. Schoenherr , et al., “Meprin β Knockout Reduces Brain Aβ Levels and Rescues Learning and Memory Impairments in the APP/Lon Mouse Model for Alzheimer's Disease,” Cellular and Molecular Life Sciences 79, no. 3 (2022): 168. 35235058 10.1007/s00018-022-04205-5 PMC8891209

[55] S. Mishra , A. Knupp , M. P. Szabo , et al., “The Alzheimer's Gene SORL1 Is a Regulator of Endosomal Traffic and Recycling in Human Neurons,” Cellular and Molecular Life Sciences 79, no. 3 (2022): 162. 35226190 10.1007/s00018-022-04182-9 PMC8885486

[56] L. Jia , M. Zhu , C. Kong , et al., “Blood Neuro‐Exosomal Synaptic Proteins Predict Alzheimer's Disease at the Asymptomatic Stage,” Alzheimer's & Dementia 17, no. 1 (2021): 49–60. 10.1002/alz.12166 PMC7984076 32776690

[57] Q. Y. Chen , Y. Yin , L. Li , Y. J. Zhang , W. He , and Y. Shi , “Geniposidic Acid Confers Neuroprotective Effects in a Mouse Model of Alzheimer's Disease Through Activation of a PI3K/AKT/GAP43 Regulatory Axis,” The Journal of Prevention of Alzheimer's Disease 9, no. 1 (2022): 158–171. 10.14283/jpad.2021.60 35098987

[58] L. Parisiadou , I. Bethani , V. Michaki , K. Krousti , G. Rapti , and S. Efthimiopoulos , “Homer2 and Homer3 Interact with Amyloid Precursor Protein and Inhibit Aβ Production,” Neurobiology of Disease 30, no. 3 (2008): 353–364. 18387811 10.1016/j.nbd.2008.02.004

[59] E. Kyratzi and S. Efthimiopoulos , “Calcium Regulates the Interaction of Amyloid Precursor Protein with Homer3 Protein,” Neurobiology of Aging 35, no. 9 (2014): 2053–2063. 24792907 10.1016/j.neurobiolaging.2014.03.019

[60] S. Matsuda and T. Senda , “BRI2 as an Anti‐Alzheimer Gene,” Medical Molecular Morphology 52, no. 1 (2019): 1–7. 29687167 10.1007/s00795-018-0191-1

[61] B. De Strooper , T. Iwatsubo , and M. S. Wolfe , “Presenilins and ‐Secretase: Structure, Function, and Role in Alzheimer Disease,” Cold Spring Harbor Perspectives in Medicine 2, no. 1 (2012): a006304–a006304. 22315713 10.1101/cshperspect.a006304 PMC3253024

[62] C.‐S. Ruan , J. Liu , M. Yang , et al., “Sortilin Inhibits Amyloid Pathology by Regulating Non‐Specific Degradation of APP,” Experimental Neurology 299, no. pt. A (2018): 75–85. 29056359 10.1016/j.expneurol.2017.10.018

[63] J. P. Bennett Jr. and P. M. Keeney , “Alzheimer's and Parkinson's Brain Tissues Have Reduced Expression of Genes for mtDNA OXPHOS Proteins, Mitobiogenesis Regulator PGC‐1α Protein and mtRNA Stabilizing Protein LRPPRC (LRP130),” Mitochondrion 53, no. July (2020): 154–157. 32497722 10.1016/j.mito.2020.05.012

[64] L. Shen , A. Yang , X. Chen , et al., “Proteomic Profiling of Cerebrum Mitochondria, Myelin Sheath, and Synaptosome Revealed Mitochondrial Damage and Synaptic Impairments in Association With 3 × Tg‐AD Mice Model,” Cellular and Molecular Neurobiology 42, no. 6 (2022): 1745–1763. 33560469 10.1007/s10571-021-01052-z PMC11421756

[65] P. C. R. Hopkins , R. Sáinz‐Fuertes , and S. Lovestone , “The Impact of a Novel Apolipoprotein E and Amyloid‐β Protein Precursor‐Interacting Protein on the Production of Amyloid‐β,” Journal of Alzheimer's Disease 26, no. 2 (2011): 239–253. 10.3233/JAD-2011-102115 21593558

[66] P. C. R. Hopkins , “Neurodegeneration in a *Drosophila* Model for the Function of TMCC2, an Amyloid Protein Precursor‐Interacting and Apolipoprotein E‐Binding Protein,” PLoS ONE 8, no. 2 (2013): 55810. 10.1371/journal.pone.0055810 PMC3567013 23409049

[67] L. Serneels , R. Narlawar , L. Perez‐Benito , et al., “Selective Inhibitors of the PSEN1–Gamma‐Secretase Complex,” The Journal of Biological Chemistry 299, no. 6 (2023): 104794. 37164155 10.1016/j.jbc.2023.104794 PMC10318456

[68] J. Taguchi , A. Fujii , Y. Fujino , et al., “Different Expression of Calreticulin and Immunoglobulin Binding Protein in Alzheimer's Disease Brain,” Acta Neuropathologica 100, no. 2 (2000): 153–160. 10963362 10.1007/s004019900165

[69] R. E. Milner , S. Baksh , C. Shemanko , et al., “Calreticulin, and Not Calsequestrin, Is the Major Calcium Binding Protein of Smooth Muscle Sarcoplasmic Reticulum and Liver Endoplasmic Reticulum,” The Journal of Biological Chemistry 266, no. 11 (1991): 7155–7165. 2016321

[70] N. Kaneshiro , M. Komai , R. Imaoka , et al., “Lipid Flippase Dysfunction as a Therapeutic Target for Endosomal Anomalies in Alzheimer's Disease,” iScience 25, no. 3 (2022): 103869. 35243232 10.1016/j.isci.2022.103869 PMC8857600

[71] M. Basso and M. Pennuto , “Serine Phosphorylation and Arginine Methylation at the Crossroads to Neurodegeneration,” Experimental Neurology 271, no. September (2015): 77–83. 25979114 10.1016/j.expneurol.2015.05.003

[72] H. L. B. Jensen , M. S. Lillenes , A. Rabano , et al., “Expression of Nucleotide Excision Repair in Alzheimer's Disease Is Higher in Brain Tissue than in Blood,” Neuroscience Letters 672, no. April (2018): 53–58. 29474873 10.1016/j.neulet.2018.02.043

[73] M. B. Miller , A. Y. Huang , J. Kim , et al., “Somatic Genomic Changes in Single Alzheimer's Disease Neurons,” Nature 604, no. 7907 (2022): 714–722. 35444284 10.1038/s41586-022-04640-1 PMC9357465

[74] J. E. Love , E. J. Hayden , and T. T. Rohn , “Alternative Splicing in Alzheimer's Disease,” Journal of Parkinson's Disease and Alzheimer's Disease 2, no. 2 (2015): 6, 10.13188/2376-922X.1000010. PMC4772657 26942228

[75] E. Ragueneau , A. Shrivastava , J. H. Morris , N. Del‐Toro , H. Hermjakob , and P. Porras , “IntAct App: a Cytoscape Application for Molecular Interaction Network Visualization and Analysis” (2021), https://academic.oup.com/bioinformatics/article‐abstract/37/20/3684/6271410. 10.1093/bioinformatics/btab319 PMC8545338 33961020

[76] R. Sandbrink , C. L. Masters , and K. Beyreuther , “APP Gene Family Alternative Splicing Generates Functionally Related Isoformsa,” Annals of the New York Academy of Sciences 777, no. January (1996): 281–287. 8624099 10.1111/j.1749-6632.1996.tb34433.x

[77] X. Wu , M. A. Hasan , and J. Y. Chen , “Pathway and Network Analysis in Proteomics,” Journal of Theoretical Biology 362 (2014): 44–52, 10.1016/j.jtbi.2014.05.031. 24911777 PMC4253643

[78] S. A. Koren , M. J. Hamm , and S. E. Meier , et al., “Tau Drives Translational Selectivity by Interacting With Ribosomal Proteins,” Acta Neuropathologica 137, no. 4 (2019): 571–583. 30759285 10.1007/s00401-019-01970-9 PMC6426815

[79] W. Chadwick , N. Mitchell , B. Martin , and S. Maudsley , “Therapeutic Targeting of the Endoplasmic Reticulum in Alzheimers Disease,” Current Alzheimer Research 9, no. 1 (2012): 110–119. 22329655 10.2174/156720512799015055 PMC4682200

[80] S. Banerjee , S. Ferdosh , A. N. Ghosh , and C. Barat , “Tau Protein‐ Induced Sequestration of the Eukaryotic Ribosome: Implications in Neurodegenerative Disease,” Scientific Reports 10, no. 1 (2020): 5225. 32251304 10.1038/s41598-020-61777-7 PMC7090008

[81] H. T. Evans , D. Taylor , A. Kneynsberg , L.‐G. Bodea , and J. Götz , “Altered Ribosomal Function and Protein Synthesis Caused by Tau,” Acta Neuropathologica Communications 9, no. 1 (2021): 110. 34147135 10.1186/s40478-021-01208-4 PMC8214309

[82] M. Petric Howe and R. Patani , “Nonsense‐Mediated mRNA Decay in Neuronal Physiology and Neurodegeneration,” Trends in Neurosciences 46, no. 10 (2023): 879–892. 37543480 10.1016/j.tins.2023.07.001

[83] G. Zuniga , S. Levy , P. Ramirez , et al., “Tau‐Induced Deficits in Nonsense‐Mediated mRNA Decay Contribute to Neurodegeneration,” Alzheimer's & Dementia: The Journal of the Alzheimer's Association 19, no. 2 (2023): 405–420. 10.1002/alz.12653 PMC9673995 35416419

[84] A. Radzisheuskaya , P. V. Shliaha , V. Grinev , et al., “PRMT5 Methylome Profiling Uncovers a Direct Link to Splicing Regulation in Acute Myeloid Leukemia,” Nature Structural & Molecular Biology 26, no. 11 (2019): 999–1012. 10.1038/s41594-019-0313-z PMC6858565 31611688

[85] M. Rengasamy , F. Zhang , and A. Vashisht , et al., “The PRMT5/WDR77 Complex Regulates Alternative Splicing Through ZNF326 in Breast Cancer,” Nucleic Acids Research 45, no. 19 (2017): 11106–11120. 28977470 10.1093/nar/gkx727 PMC5737218

[86] L. Zhang , Z. Qi , and J. Li , et al., “Roles and Mechanisms of Axon‐Guidance Molecules in Alzheimer's Disease,” Molecular Neurobiology 58, no. 7 (2021): 3290–3307. 33675023 10.1007/s12035-021-02311-2

[87] B. Wang , H. Li , S. A. Mutlu , et al., “The Amyloid Precursor Protein Is a Conserved Receptor for Slit to Mediate Axon Guidance.” eNeuro 4 no. 3 (2017), 10.1523/ENEURO.0185-17.2017. PMC5534435 28785723

[88] S. Bolognin , E. Lorenzetto , G. Diana , and M. Buffelli , “The Potential Role of Rho GTPases in Alzheimer's Disease Pathogenesis,” Molecular Neurobiology 50, no. 2 (2014): 406–422. 24452387 10.1007/s12035-014-8637-5

[89] R. Cai , Y. Wang , and Z. Huang , et al., “Role of RhoA/ROCK Signaling in Alzheimer's Disease,” Behavioural Brain Research 414, no. 113481 (2021): 113481. 34302876 10.1016/j.bbr.2021.113481

[90] B. J. Aguilar , Y.i Zhu , and Q. Lu , “Rho GTPases as Therapeutic Targets in Alzheimer's Disease,” Alzheimer's Research & Therapy 9, no. 1 (2017): 97. 10.1186/s13195-017-0320-4 PMC5732365 29246246

